# Using affinity propagation for identifying subspecies among clonal organisms: lessons from *M. tuberculosis*

**DOI:** 10.1186/1471-2105-12-224

**Published:** 2011-06-02

**Authors:** Claudio Borile, Mathieu Labarre, Silvio Franz, Christophe Sola, Guislaine Refrégier

**Affiliations:** 1 LPTMS, CNRS and Univ. Paris-Sud, UMR8626, Bat. 100, 91405 Orsay, France; 2 Dipartimento di Fisica "G. Galilei", Università di Padova, via Marzolo 8, I-35131 Padova, Italy; 3 IGM, CNRS and Univ. Paris-Sud, UMR8621, Bat. 400, F-91405 Orsay cedex, France; 4 Unité de Génétique Mycobactérienne, Institut Pasteur, Paris, France

**Keywords:** asexual organisms, species delineation, epidemiology, DR locus, Clustered Regularly Interspaced Short Palindromic Repeats (CRISPR)

## Abstract

**Background:**

Classification and naming is a key step in the analysis, understanding and adequate management of living organisms. However, where to set limits between groups can be puzzling especially in clonal organisms. Within the *Mycobacterium tuberculosis *complex (MTC), the etiological agent of tuberculosis (TB), experts have first identified several groups according to their pattern at repetitive sequences, especially at the CRISPR locus (spoligotyping), and to their epidemiological relevance. Most groups such as "Beijing" found good support when tested with other loci. However, other groups such as T family and T1 subfamily (belonging to the "Euro-American" lineage) correspond to non-monophyletic groups and still need to be refined. Here, we propose to use a method called Affinity Propagation that has been successfully used in image categorization to identify relevant patterns at the CRISPR locus in MTC.

**Results:**

To adequately infer the relative divergence time between strains, we used a distance method inspired by the recent evolutionary model by Reyes *et al*. We first confirm that this method performs better than the Jaccard index commonly used to compare spoligotype patterns. Second, we document the support of each spoligotype family among the previous classification using affinity propagation on the international spoligotyping database SpolDB4. This allowed us to propose a consensus assignation for all SpolDB4 spoligotypes. Third, we propose new signatures to subclassify the T family.

**Conclusion:**

Altogether, this study shows how the new clustering algorithm Affinity Propagation can help building or refining clonal organims classifications. It also describes well-supported families and subfamilies among *M. tuberculosis *complex, especially inside the modern "Euro-American" lineage.

## Background

The advent of powerful genotyping methods, either by global sequencing or by high-throughput analysis of variation at specific loci (mini- or micro-satellites [1]; CRISPR (Clustered Regularly Interspaced Short Palindromic Repeats) loci [2,3]; SNPs [4]), provides masses of genetic data that need to be compared and clustered. Most widely used comparison methods are phylogenetic methods *i.e. *hierarchical clustering, building tree-like structures to display the diversity. These methods consider that each individual forms a cluster and repeatedly merge the most similar clusters based on pairwise distances (Phenetics such as Neighbour-Joining), or try to infer the tree that most fits the data (Cladistics such as Maximum Likelihood, Bayesian analysis) using an appropriate evolutionary model of the compared characters. This provides a continuous pattern of how divergent organisms are. Other comparison methods consist in finding relevant clusters, a process referred to as partitioning. A method made popular by the software Structure [5], and referred to as model-based clustering, consists in using Bayesian methods to assign individuals in a pre-determined number of populations. The assumption underlying this software is that the population conforms Hardy-Weinberg hypotheses *i.e. *refers to organisms reproducing sexually, with random pairing inside the population. This assumption is theoretically very problematic for clonal organisms, although practice has shown that it can provide meaningful results [6], partly because some parameters can be set to mimic poor mixture inside populations. Other methods have been developed outside biology, for instance to categorize images [7,8]. They use similarity to group data in spherical clusters well represented by their centroid (also called representative or exemplars), and have already been tentatively used to classify human genetic data [9]. This method awaits further experimental validation on large datasets.

Clustering methods can be applied to different types of loci, ranging from repetitive sequences such as insertion sequences, micro-, mini-satellites or the CRISPR loci to single nucleotide polymorphisms (SNPs), provided an appropriate method is available to calculate the distance between individuals. Such methods usually rely on a model of the mutation process. Which loci should be targeted depends on the mean divergence time between individuals, as repetitive sequences mutate faster than SNP loci. Several mutation models have been developed for DNA sequence with point mutations [10]. For repetitive sequences (micro- and mini -satellites), categorical distance or the Stepwise Mutation Model (SMM [11]) are mostly used.

CRISPR loci (Clustered Regularly Interspaced Short Palindromic Repeats) form a new family of repetitive sequences [12,13]. They consist in the repetition of 24 to 47 bp sequences called Direct Repeats (DR) separated by unique sequences called spacers (from 26 to 72 bp). The constitutive unit is therefore the combination of one DR and one spacer, and presently described CRISPR loci have from 2 to 249 units [13]. These repetitions are surrounded by protein-encoding sequences called *cas *genes (derived from *C*RISPR-*as*sociated genes). The whole locus confers resistance to bacteriophages and plasmids in *Streptococcus thermophilus *[14,15] and in *Escherichia coli *when overexpressed [16]. Resistance to the corresponding organisms is under investigation in species where spacers are homologous to foreign DNA [17]. They exhibit a quite high mutation rate so that they have proven useful for epidemiological studies. Describing the presence or absence of 43 spacers of *M. tuberculosis *CRISPR locus has become a routine technique in any tuberculosis reference center and is referred to as spoligotyping for *sp*acer *oligo*nucleotide *typing *[18]. Pairwise comparisons of binary profiles can provide a distance matrix that has been used to infer phylogenetic relationships. The most common approach to infer relationships using spoligotype patterns uses the Jaccard index (same principle as Hamming distance or Dice coefficient) as distance [19], counting the proportion of spacers that are present in both profiles. The distance matrix, either made of pure spoligotyping data or combining them with minisatellite data, is usually processed using UPGMA or NJ algorithm to build a dendrogram or a phylogenetic tree [20]. A more elaborate approach using the *Zipf *distribution and the evolutionary dynamics of CRISPR loci has proven more relevant to infer phylogenetic relationships for the *M. tuberculosis *complex [21] but is not implemented in a user-friendly software yet and does not propose assignations for all currently described spoligotype patterns.

The worldwide database of spoligotyping in *M. tuberculosis *complex is called SpolDB (the latest public version being SpolDB4), and has helped identifying recurrent signatures in CRISPR profiles [22-24]. These signatures, mainly based on the absence of adjacent spacers, led to the naming of large clonal families, the monophyly of which has been confirmed through other markers such as minisatellites (referred to as MIRU-VNTR for Mycobacterial-Interspersed-Repetitive-Units-Variable Number of Tandem Repeats), Region of Deletions (RDs) and SNPs [6,25,26]. Main acknowledged families are EAI for East-African-Indian (later referred to as "Indo-Oceanic" by Gagneux *et al.*), *M. africanum 1 *and *2 *(later *"West-african 2" *and *"West-African 1"*), animal strains (*M. bovis*, *M. caprae*, *M. pinnipedii, M. microtii*), CAS for "Central-Asia" (later "East-African-Indian"), Beijing, X, Haarlem, LAM for "Latino-American and Mediterranean", T and MANU (also designated as T ancestor) [23,27]. Monophyly of each of the LAM, T and Haarlem families has been partly invalidated. However, the larger lineage to which they belong and that is characterized by the 33-36 spacers deletion at the CRISPR profile (merging T, LAM, X, Haarlem families and S subfamily) has been confirmed and designated as the "Euro-American" lineage [27]. It corresponds to the Principal Genetic Groups (PGG) 2 and 3 as defined by Sreevatsan *et al. *[28]. Altogether deletions in spoligotype patterns have proven to contain phylogenetic information and allow most strains be assigned to the families described above. Assignations performed by experts are reported in SpolDB4 database, patterns carrying no or contradictory signatures been labeled as "U" for "Unknown or Unclassified". To circumvent the dependence on experts' analysis, the Bennett's laboratory proposed automatized classification of spoligotype patterns using Bayesian algorithms and a distance method taking into account the deletion process by which spoligotype patterns evolve. They provide an on-line tool called Spotclust [29] to assign each spoligotype to a family, either one described in SpolDB3 [30] or one of the 6 large families proposed by Gagneux and coworkers [31].

Here we wanted to take advantage of a recently developed algorithm, Affinity Propagation, to confirm and extend these methods. This algorithm identifies references for every data point so that data are grouped and centered on these references while a specific cost function is minimized. The cost of adding a new reference point, assigned by the user, determines the final number of clusters. Prior to the use of this algorithm, we tested different distances to calculate pairwise distances between spoligotype patterns. We took advantage of previously identified references and expert assignation to rank these distances, some of which are derived from previously proposed evolutionary models [21,31]. The distance that best identified the appropriate reference for each spoligotype pattern was implemented in the Affinity Propagation algorithm to identify relevant subfamilies among *M. tuberculosis *complex (MTC). These families partly correlated with previously identified subfamilies.

Altogether, this approach allowed us to assess the robustness of previously identified sublineages among MTC, to identify new relevant sublineages and to provide re-assignations of the spoligotype patterns described in SpolDB4. These re-assignations interestingly matched those of studies using VNTR and/or SNP data.

## Results

### Comparison of classifications based on new distances or on Jaccard index to expert classification of SpolDB4

**Figure 1:**
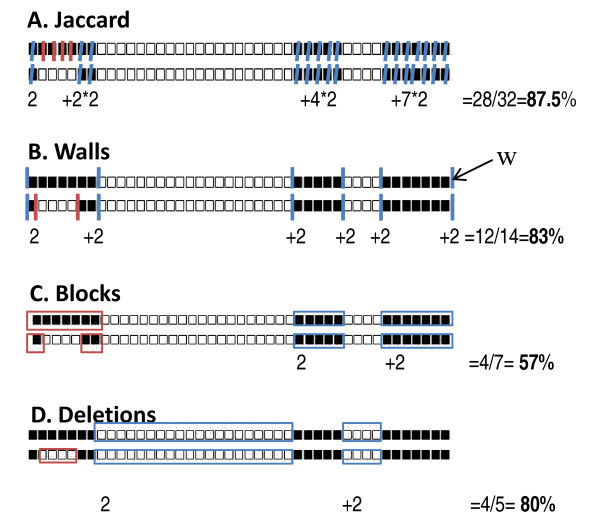
**Distance methods**. **A: **classically implemented Jaccard index. **B-D**: newly proposed distance methods. w = Domain Walls also referred to as walls. Numbers below the spoligotype patterns count the number of their common features: either the number of common spacers (A), common walls (B), common blocks (C), or common deletions (D). These numbers are summed and divided by the total number of features in the two spoligotype patterns to obtain the similarity between the two spoligotypes.

**Figure 2:**
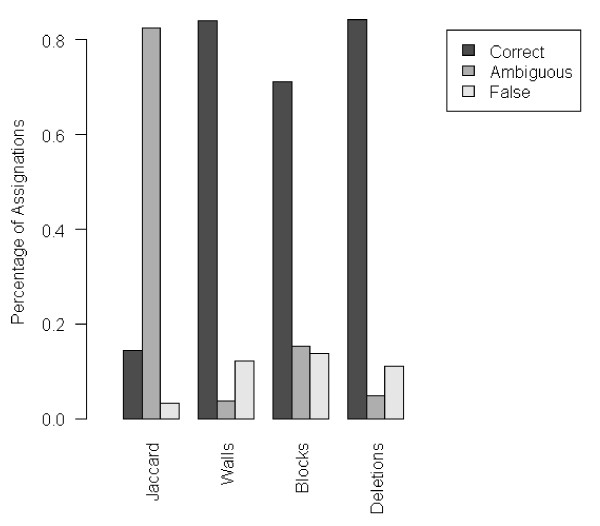
**Assignations matches between SpolDB4 and the different distance methods on whole SpolDB4 database (n = 1937 SIT)**. References are those described in Table 1. Assignations were performed according to the reference for which the distance was the lowest. The patterns for which the most similar reference is the same as that indicated by its SpolDB4 assignations, were scored as "Correct". Note that "Domain Walls" and "Deletions" have equally high values of assignations agreeing with the expert classification. When the method identified two identically similar references for a pattern, this pattern was scored as Unassigned and described as Ambiguous assignation. Ambiguity was the lowest with "Domain Walls" method.

Clustering of CRISPR patterns (spoligotypes) of *M. tuberculosis complex *is commonly done using the Jaccard index as distance [32]. This index counts the shared spacers without taking into account their spatial organization. However, it has been shown that adjacent spacers have a higher probability to be simultaneously deleted [21], and this feature has been used by experts to define *M. tuberculosis *complex families and subfamilies [22,23] in the international database SpolDB [33]. We wanted here to identify a distance conducing to the best concordance of spoligotype assignations at the family level, as available in SpolDB4 database [23]. We retained the ten widely acknowledged families: *M. africanum*, Animal strains (grouping *M. bovis*, *M. pinnipedii*, *M microtii*, and *M caprae*), Beijing (herein also referred to as Beij), CAS, EAI, Haarlem (also referred to as H), LAM, MANU, T and X [25]. Each is characterized by a different spoligotype signature and thus a different reference profile [22,33] (Table 1). In addition to Jaccard index, we set up three methods to compute the distance between pairs of patterns: "Domain Walls" measuring the proportion of shared limits of blocks of spacers in the CRISPR locus, "Blocks" measuring the proportion of shared blocks of spacers, and "Deletions" measuring the proportion of shared blocks of deleted spacers (see Methods and Figure 1). We implemented these four methods to compute the distances of each spoligotype of SpolDB4 database [33] to the reference profiles of the ten families (see Table 1). For each method, depending on the reference to which it was most similar, each spoligotype was assigned to one of the ten families. Spoligotype patterns for which two references were equally similar were coined as Unassignable. These assignations were compared to the SpolDB4 classification. The Jaccard method rarely associated the spoligotype patterns to their reference (only 12.3% of correctly assigned spoligotypes, see Figure 2). The methods that best fitted the expert classification were the "Deletions" (84.2% of correctly assigned spoligotype patterns, n_correct _= 1402), and the "Domain Walls" method (84.0%, n_correct _= 1399). These methods also provided the smallest amount of assignations that differed from those of the experts ("Deletions": 11.0%, n _false _= 183; "Domain Walls": 12.3%, n _false _= 204). These methods thus appear to be the best for fitting the expert classification out of the four methods we tested.

**Table 1:** References of the ten best acknowledged *M. tuberculosis *complex families

SIT	SpolDB4 classification		Reference Spoligotype pattern
		
	family	subfamily	
1	BEIJ	BEIJ	
26	CAS	CAS1	
42	LAM	LAM9	
50	H	H3	
53	T	T1	
100	MANU	MANU1	
119	X	X1	
236	EAI	EAI5	
181	AFRI	AFRI1	
482	animal	BOV1	

BEIJ = Beijing also referred to as East Asia; CAS = Central Asia also referred to as East Africa and India; LAM = Latino-American and Mediterranean; H = Haarlem; EAI = East African Indian.

### "Deletions" method succeeds in correcting false SpolDB4 assignations

**Figure 3:**
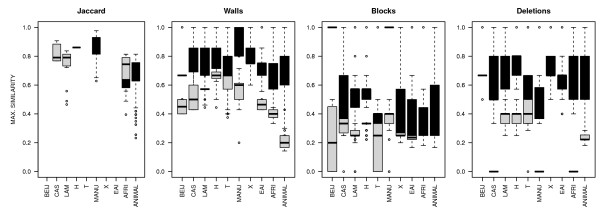
**Plot of similarity to their reference for patterns assigned as the expert classification (Black) and differently than the expert classification (Gray)**. *Black boxes*: box plots of the similarity to their reference for patterns with congruent classification between distance-based method and expert-defined. Boxes extend from 0.25 to 0.75 quartiles, and whiskers to the most extreme values. Median is highlighted by a thick bar. *Grey boxes*: box plots of the similarity to their reference for patterns with incongruent classification between distance-based method and expert-defined. Families for which no spoligotype patterns gave ambiguity show a single (black) box, corresponding to the mean similarity of congruent assignations (Beij, X and EAI with "Deletions" method; X for "Domain Walls"). Families for which no spoligotype patterns were found similar to the reference show no data (Beij, T, X, EAI with "Jaccard" method). BEIJ = Beijing; afri=*M. africanum*; CAS = Central Asia; LAM = Latino-American and Mediterranean; H = Haarlem; EAI = East African Indian.

Some families' assignations provided by SpolDB4 have been debated. For instance, patterns classified as LAM7-TUR [32] have been found not to be related to the LAM family as strains carrying that pattern do not share the ligB^1212 ^mutation that defines the LAM family [34]. Such strains were instead related to T-strains [35] and were renamed TUR. All methods tested here except the "Deletions" method still assigned them to LAM family, including Spotclust. The "Deletions" method assigned them all (n_LAM7-TUR _= 8) to the T family as did methods using VNTRs [35] or SNPs [34] (Table 2). Similarly, all spoligotypes assigned to the H4 subfamily (n_H4 _= 34) in SpolDB4 were recently excluded from the Haarlem family based on them not carrying the mgtC^545 ^mutation [34]. They were renamed "Ural" and appropriately assigned to the T family by the "Deletions" method only (Table 2). Hence, part of the assignations suspected to be wrong with the "Deletions" method as compared to expert classification may in fact correct previous classification errors. The "Deletions" method thus recognizes phylogenetic lineages better than "Domain Walls" method and likely at least as well as the expert eye and Spotclust. This is further supported by the clear gap between the similarity of correctly assigned spoligotype patterns to their reference (Figure 3D, black boxes in the Deletions plot) and the highest similarity to any reference of patterns assigned differently than by the expert (light gray boxes) specifically with the "Deletions" method.

**Table 2:** Assignations of LAM7, H4 and selected "U" spoligotype patterns from SpolDB4, according to different methods.

	spoligotype pattern	SpolDB4		Recent litterature	Deletions	Domain Walls	SpotClust subfamily	
						
SIT		family	Sub-family	assignation	family	family	SpolDB3-based	RIM
41		LAM	LAM7	T-TUR	**T**	*LAM*	*LAM9*	*N19*
186		LAM	LAM7	T-TUR	**T**	*LAM*	*LAM9*	*N19*
367		LAM	LAM7	T-TUR	**T**	*LAM*	*LAM9*	*N19*
930		LAM	LAM7	T-TUR	**T**	*LAM*	*LAM1*	*N19*
1261		LAM	LAM7	T-TUR	**T**	*LAM*	*LAM9*	*N19*
1589		LAM	LAM7	T-TUR	**T**	*LAM*	*LAM3*	*N2*
1924		LAM	LAM7	T-TUR	**T**	*LAM*	*LAM9*	*N19*
1937		LAM	LAM7	T-TUR	**T**	*LAM*	*LAM9*	*N19*
								
35		H	H4	T-Ural	**T**	*H*	*H3*	*N34*
262		H	H4	T-Ural	**T**	*H*	*H3*	*N34*
361		H	H4	T-Ural	**T**	*H*	*H3*	*N34*
399		H	H4	T-Ural	**T**	*H*	**T2**	*N34*
596		H	H4	T-Ural	**T**	*H*	*H3*	*N34*
597		H	H4	T-Ural	**T**	*H*	*H3*	*N34*
656		H	H4	T-Ural	**T**	*H*	*H3*	*N34*
762		H	H4	T-Ural	**T**	*H*	*H3*	*N34*
777		H	H4	T-Ural	**T**	*H*	*H3*	*N34*
817		H	H4	T-Ural	**T**	*H*	*H3*	*N34*
920		H	H4	T-Ural	**T**	*H*	*H3*	*N34*
921		H	H4	T-Ural	**T**	*H*	*H3*	*N34*
922		H	H4	T-Ural	**T**	*H*	*H3*	*N34*
1117		H	H4	T-Ural	**T**	*H*	*H3*	*N34*
1134		H	H4	T-Ural	**T**	*H*	*H3*	*N34*
1165		H	H4	T-Ural	**T**	*H*	*H3*	*N34*
1174		H	H4	T-Ural	**T**	*H*	*H3*	*N34*
1242		H	H4	T-Ural	**T**	*H*	*U*	*N34*
1269		H	H4	T-Ural	**T**	*H*	*H3*	*N34*
1276		H	H4	T-Ural	**T**	*H*	*H3*	*N34*
1281		H	H4	T-Ural	**T**	*H*	*H3*	*N34*
1292		H	H4	T-Ural	**T**	*H*	*H3*	*N34*
1447		H	H4	T-Ural	**T**	*H*	*H3*	*N34*
1448		H	H4	T-Ural	**T**	*H*	*H3*	*N34*
1457		H	H4	T-Ural	**T**	*H*	*H3*	*N34*
1568		H	H4	T-Ural	**T**	*H*	*H3*	*N34*
1581		H	H4	T-Ural	**T**	*H*	*H3*	*N34*
1384		H	H4	T-Ural	**T**	U	**T3**	**N40**
1446		H	H4	T-Ural	**T**	U	*H3*	*N34*
1452		H	H4	T-Ural	**T**	U	*H3*	*N34*
1455		H	H4	T-Ural	**T**	U	U	*N34*
1456		H	H4	T-Ural	**T**	U	*H3*	*N34*
1461		H	H4	T-Ural	**T**	U	*H3*	*N34*
1480		H	H4	T-Ural	**T**	U	*LAM9*	*N19*
								
105		U	U	H	U	*Afri*	*LAM7*	*N29*
1274		U	U	LAM	U	*Afri*	*H1*	*N5*
1531		U	U	X	**X**	**X**	**X1**	**N44**

"Recent literature assignation" represents the standard, and refers to studies using loci other than the CRISPR locus: T-TUR classification has been suggested both by Millet et al. [35] and Abadia et al. [34] based respectively on VNTR signature and SNPs signatures. T-Ural classification has been suggested by Kovalev et al. [36] as they clustered with H37Rv strains and Abadia et al. [34]. RIM: Randomly Initialized Model. N ... families as defined by Spotclust are described on their web-site based on what SpolDB4 families/sub-families are mostly represented: N2 family is described as LAM3-rich; N5 as H1-rich; N19 as LAM-rich; N29 as LAM+EAI-rich; N34 as H3+S-rich; N40 as T3-africanum-rich; N4 as X1-H37Rv-rich. The assignations matching the standard are shown in bold characters. Assignations failures are shown in italic. Note that the "Deletions" method provides the highest number of exact assignations and the least assignation failures.

Interestingly, Beijing, X and EAI families exhibited no incongruence between the "Deletions" and the expert method (no light gray box), suggesting that these families are clearly and appropriately defined. As reported above (Figure 2), the Jaccard method failed to assign most spoligotype patterns to any family; for instance, no spoligotype patterns could be assigned to BEIJ, EAI or X families (Figure 3A) with a maximum similarity to any reference not reaching 20% for BEIJ family (Additional file 1). "Domain Walls" and "Blocks" methods provided either poor resolution between correctly and wrongly assigned spoligotype patterns (Figure 3A and 3C), or a lower number of families with no discrepancy with the expert classification (only the X family with the "Domain Walls" method, Figure 3B).

### Assignations of U spoligotype patterns

**Figure 4:**
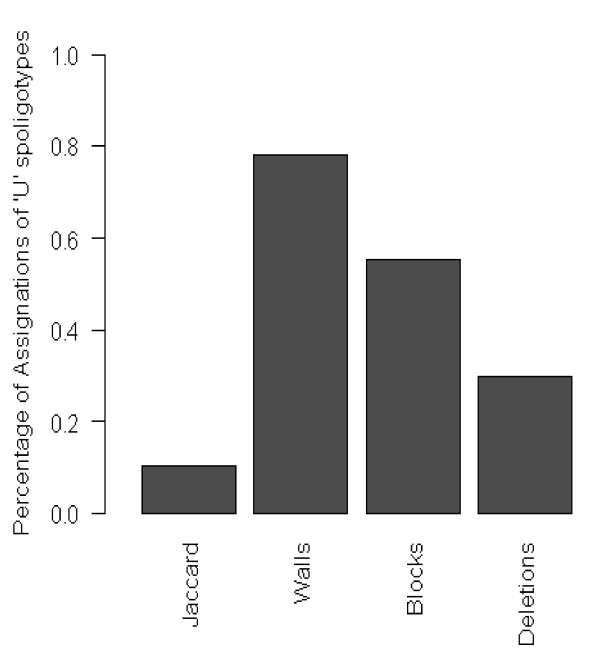
**Assignations of 'U" patterns managed by the different methods**. Percentage was calculated based on the 272 "U" patterns found in SpolDB4.

Assignations thus seem phylogenetically relevant using the "Deletions" method and the references of the well-acknowledged families. We thus propose an alternative spoligotype patterns classification on the 1939 spoligotypes reported in SpolDB4 (Additional File 2). Assignation rate of "U" (Unclassified) patterns was relatively low with this method as compared to others (81 out of 272 U patterns, *i.e. *29.8%, Figure 4) but may be more reliable as exemplified by three U patterns recently assigned [34]: "Deletions" method could only assign one of them but without error whereas two of the three assignations provided by the "Domain Walls" method and Spotclust algorithm were erroneous (Table 2, SIT 105, 1274 and 1531).

### Automatized identification of references by Affinity Propagation clustering

**Figure 5:**
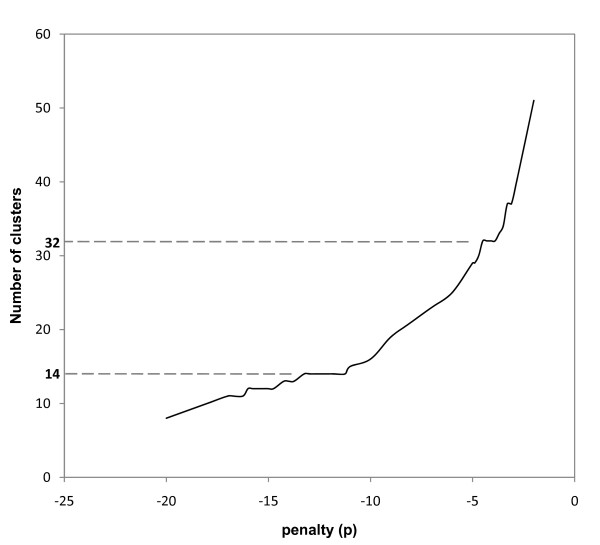
**Number of clusters found by Affinity Propagation as a function of the penalty *p *for the distance between a data point and its reference**. Note that two plateau can be detected, at 14 and 32 clusters respectively, indicating that the corresponding clustering is robust, and therefore might be relevant.

The "Deletions" method is highly useful to classify spoligotype patterns in the described families, but this classification highly depends on the identification of references. These references are widely acknowledged for major families but the relevance of finer classification is recurrently debated [25,27,35]. Affinity propagation is an algorithm that identifies a representative (also called exemplar) for each data point in an iterative manner until the chosen configuration of exemplars minimizes a suitable cost function that depends on the choice of the clusters (see Methods). A parameter set by the user (that we denote as 'penalty', *p*) determines an additional cost for every exemplar found. When *p *is low (high negative value), large clusters are built where some data points have relatively low similarity with their representative. As *p *increases, the clusters reach smaller sizes so that they become

numerous, and the mean similarity with the representative increases. Interestingly, when the number of clusters does not vary even if the penalty changes, this indicates that the data points are not evenly distributed, *i.e.*, form meaningful clusters. When applying this method to the SpolDB4 dataset, relevant numbers of clusters were found to be 14 and 32 (Figure 5). The mean similarity with the representative was higher using Affinity Propagation as compared to K-Means or with Bionumerics applying hierarchical clustering (Additional File 3). The clustering in 14 clusters reproduced most of the 10 references identified by the experts (references for animal strains, CAS, EAI, H, LAM, T, and X, Table 3). However, H family was divided in H1 and H3, none of them including the H4. H4 was grouped with T spoligotype patterns as suggested by previous studies that renamed it Ural [34,36]. We propose some renamings according to major families represented in each cluster (Table 3). When performing clustering with 32 clusters, many of the SpolDB4 subfamilies were identified. Some of them were however merged such as africanum2-africanum3, Bovis1-3, pinnipedii-microtii, LAM2-LAM1-LAM5, X1 and 3 in X, etc. (Table 4). Among EAI, four meaningful subfamilies were identified whereas only 2 were among LAM. This suggests that the LAM family was oversplitted in the expert classification. In contrast, seven subfamilies were found among the T family. Two of them exhibited complex signatures with few spoligotype patterns actually matching the whole signature (for example, n = 5/29 among T1a). Subfamilies T2, T3 and T5 were confirmed by this method. One family still had SIT53 (T1) as a reference indicating that many spoligotypes (n = 261) still cannot be further classified according to their spoligotype pattern. Last, one family was created from U spoligotypes and has SIT 458 as a reference. Most patterns carried the deletion of spacers 29 to 34 that could constitute a new significant signature. Countries in which the corresponding SITs were most abundant surround the Indian Ocean: Madagascar, Thailand, India and Vietnam (Table 5). We thus named it SEA1 (South East Asia 1) (Table 4). The significance of this signature compared to the classical EAI signature, which differs only by the presence of spacer 33, remains to be established.

**Table 3:** References after Affinity Propagation clustering for n_clusters _= 12.

AP-family	Reference			Majoritary SpolDB4 family		
			
	SpolDB4 subfamily	SIT	Spoligotype pattern	Family	Proportion in the AP-family	Total Nb
**animal1(Bov1-3-Cap-Mic-Pin)**	bovis_1	**482**		Animal	0.888	206
animal2(Bov2)	bovis_2	683		Animal	0.621	66
Beij-afri	BEIJ	255		Afri	0.339	56
**CAS**	CAS_1	**26**		CAS	0.760	96
**EAI**	EAI_5	**236**		EAI	0.84	250
H1-2	H_1	47		H	0.853	68
**H**3	H_3	**50**		H	0.874	111
**LAM**_(9-3-11-6-4)_	LAM_9	**42**		LAM	0.721	179
T2	T_2	52		T	0.545	145
T3-LAM_(2-5)_	LAM_2	17		LAM	0.432	148
**T**-(Ural-H3-LAM**_10-7)_)**	T_1	**53**		T	0.823	351
S(&U)	S	34		T	0.554	74
T(&U)	T_1	173		T	0.420	81
**X**	X_1	**119**		X	0.75	108

BEIJ = Beijing (also East Asia); afri=*M. africanum*; CAS = Central Asia (also East Africa and India); LAM = Latino-American and Mediterranean; H = Haarlem; EAI = East African Indian. Confirmed families are shown in bold.

**Table 4:** References after Affinity Propagation clustering for n_clusters _= 32.

AP-subfamily naming	reference			Most represented subfamilies			Nb of spoligotype patterns
			
	Classical subfamily naming	SIT	spoligotype (43 format)	First most represented subfamily		Second most repr. family	
						
				Subfamily	Prop.		
**Afri1**	AFRI1	181		AFRI1	0.531	AFRI	32
**Afri2-3**	AFRI2	331		AFRI2	0.364	AFRI3	22
**Beij**	BEIJ	1		BEIJ	0.842	U	19
**Bov1-3**	BOV1	482		BOV1	0.585	BOV	159
**Bov2**	BOV2	683		BOV2	0.467	BOV	45
**Cap**	CAP	647		CAP	0.75	U	20
**CAS**	CAS1	26		CAS1	0.487	CAS	80
**EAI1**	EAI1	48		EAI1	0.804	U	46
**EAI3-5 (del2-3-37-38-39)**	EAI2	11		EAI5	0.383	EAI3	55
**EAI2 (del3-20-21)**	EAI2	19		EAI2	0.5	U	48
**EAI**	EAI5	236		EAI5	0.651	EAI4	86
**EAI6 (del23-37)**	EAI6	292		EAI6	0.5	EAI5	42
**H1-2**	H1	47		H1	0.790	U	62
**H3**	H3	50		H3	0.927	U	96
**Ural**	H4	262		H4	0.714	U	28
**LAM5-2-1(del3-13)**	LAM2	17		LAM5	0.207	U	92
**LAM3**	LAM3	33		LAM3	0.455	U	33
**LAM**	LAM9	42		LAM9	0.574	LAM11	136
**Manu**	MANU2	54		MANU2	0.793	U	29
**Pin-Mic**	PIN	637		BOV	0.391	U	23
**S**	S	34		S	0.678	U	59
T (T1-H3-Lam10-Cam)	T1	53		T1	0.828	H3	261
T1a (del5-40-43)	T1	833		T1	0.484	U	31
**T1b (del21)**	T1	291		U	0.367	T1	30
**T1c (del15)**	T2	118		T1	0.432	U	37
**T2 (del40)**	T2	52		T2	0.521	U	119
**T3 (del13)**	T3	37		T3	0.373	U	59
T4 (del19-23-24-38-39)	T4	39		T1	0.406	T4	32
**T5 (del23)**	T5	44		T5	0.561	U	41
**X**	X1	119		X1	0.492	U	61
**X2**	X2	137		X2	0.824	T1	34
**SEA1 (del29-34)**	U	458		U	0.955	CAS	22

BEIJ = Beijing (also East Asia); afri=*M. africanum*; CAS = Central Asia (also East Africa and India); LAM = Latino-American and Mediterranean; H = Haarlem; EAI = East African Indian. Confirmed subfamilies are shown in bold.

**Table 5:** Spoligotype patterns clustered with SIT 458 with Affinity Propagation when n_clusters _= 32.

SIT	Spoligotype pattern	SpolDB4 assignation	Main country
458*		U	THA
354		U	GBR
526		U	GNB
527		U	GNB
863		U	BRA
1172		U	EST
1186		U	THA
1187		U	THA
1374		U	MYS
1386		U	BGD
1436		U	BGD
1462		U	GEO
1515		U	MDG
1518		U	MDG
1519		U	MDG
1520		U	MDG
1521		U	MDG
1524		U	MDG
710		U	NLD
405		U	VNM
426		CAS_2	USA
523		U	MYS

Note that most of the patterns carry the 29-34 spacers deletion, and that most of them are unclassified by SpolDB4. "Main country" refers to the country where the highest number of isolates carrying this pattern were found according to SpolDB4 [33]. * indicates the spoligotype proposed as a reference by the Affinity Propagation algorithm. The countries are identified via the ISO3166-1 alpha-3 code. THA = Thailand; GBR = United Kingdom; GNB = Guinea-Bissau; BRA = Brazil; EST = Estonia; MYS = Malaysia; BGD = Bangladesh; GEO = Georgia; MDG = Madagascar; NLD = Netherlands; VNM = Vietnam; USA = United States.

## Discussion

Here we first validated a simple distance method that can be used to classify CRISPR genetic profiles based on a worldwide *M. tuberculosis *spoligotype database. Second, using a recent clustering algorithm exploiting a different approach with respect to those commonly used in the biological sciences community, we could identify an alternative *M. tuberculosis *classification. The comparison between the largely validated expert classification described in the international database SpolDB4 and our alternative classification validates our approach for *M. tuberculosis *CRISPR profiles, opening the way for its use for other bacterial species where CRISPR were shown to provide interesting typing information [16].

### Clustering power of CRISPR patterns

*M. tuberculosis *complex (MTC) has been infecting humans for at least 2600 years [37] and could be 20,000 years old or even much older [6,38]. Despite its restricted genetic diversity even between human and animal strains [39,40], phylogenetical relationships have been detected using polymorphic DNA sequences [41,42]. CRISPR loci characterized using the "spoligotyping" technique have been used to define families through the use of so-called signatures *i.e. *the absence and presence of characterized units of CRIPSR loci, the spacers [43]. Most of these families found independent support such as host range or congruence with independent genetic markers [22,23,44], even SNPs and Regions of Deletion [26,34,45]. However, some of them were "ill-defined" *i.e. *had a signature that was shared by several other groups, and others were defeated by independent loci: H4 subfamily was renamed Ural as it was related to T strains and not H strains [34,36], LAM7 and LAM10 were renamed TUR and Cameroon respectively as they are unrelated to LAM strains [6,34,35]. As a consequence, the use of CRISPR patterns to infer phylogenetical relationship was recurrently discussed [44,46].

We used here an automatized approach for clustering CRISPR patterns. Our clusters largely reproduced the well-acknowledged MTC families and provided meaningful clustering for Ural, TUR and Cameroon. In fact, the misclassification of Ural among Haarlem family was due to the merging of all signatures having spacer 31 deleted and spacer 32 present disregarding the left border of the deletion. This classification criterion is not relevant knowing the evolutionary dynamics of CRISPR loci due either to the insertion of IS*6110 *elements or to the deletion of one of several adjacent spacers. This kind of errors is avoided if comparison is performed using an algorithm identifying complete signatures (left and right borders of the deletions) as included in our automatized approach (see below for a detailed discussion on methods used to calculate distances between strains).

Still, the fact that some families are "ill-defined" is an intrinsic problem of spoligotyping: CRISPR loci in *M. tuberculosis *are relatively short and they evolve at a rate that cannot exclude the absence or the insufficient number of mutation in some lineages. This intrinsically limits the power of our study, *i.e. *we cannot classify all strains, and not all of them with the same precision. However, this problem does not affect the assignation quality of the strains we classify which are in fact numerous (more than 80%).

We thus argue that CRISPR profiles evolving by the insertion of transposable elements or by deletion such as those of *M. tuberculosis *contain relevant information for clustering and even inference of some phylogenetic links. The targeted locus must however not be missing for the individuals to be classified. To avoid this pitfall, the use of CRISPR loci should restrict to recently diverged groups as is the *M. tuberculosis *species complex (more than 99.9% identity). Such organisms uncover diverse human pathogens such as *Yersinia pestis*, *Salmonella enterica*, *Bacilllus anthracis*, *Mycobacterium leprae and Mycobacterium ulcerans*. Still, the use of CRISPR profiles in phylogenetic reconstructions would benefit from further developments and validations for species with still active CRISPR loci.

### Distance methods for CRISPR profiles

If CRISPR can be used to infer phylogenetic relationship, the evolutionary model or distance method used during the inference is also of great importance. Several developments had been proposed until now. We want to discuss here what our approach adds to previous ones.

CRISPR profiles (spoligotype patterns) form a sequence of binary data. As such, it has been analyzed with tools developed for binary information such as the Jaccard Index that focuses on the sharing of every unit in the profile (here the spacers) taken independently. This however ignores an essential feature of the corresponding CRISPR locus: that it evolves by the loss of spacers. These losses can occur either because of the insertion of a transposable element that disrupts the sequence used in the spoligotyping technique, or by deletion. Deletions can occur for several spacers at once, even if the frequency of large deletions may be lower [21]. As a consequence, the distance between two patterns, one carrying many spacers and the other carrying one large deletion, should not be considered as proportional to the number of spacers that were lost (as done by the Jaccard index), but as corresponding to a single mutation event. The methods proposed by the Bennett laboratory [30,31] take into account the deletion process and add a probability function that best mimics the frequency of deletion size. In Spotclust, a Bayesian algorithm incorporating the inference of ancestral spoligotype patterns based on SpolDB3 database is used to assign spoligotypes to SpolDB3 subfamilies or to families built using a Randomly Initialized Model (RIM) [30]. We showed here that, by simply using expert-defined references of main families and the "Deletions" distance method that is based on deletion signatures, we could better assign Unknown spoligotype patterns than Spotclust as well as correct previous erroneous assignations in SpolDB4 classification such as those to LAM7(TUR) [29]. For Spotclust algorithm, this was true for both the SpolDB3-based classification and the Randomly Initialized Model. The reason for that could be either that the size of the database used by Spotclust was too small to capture evolutionary steps relevant to MTC evolution, or that Bayesian statistical inferences are too dependent on priors.

### Performance of the Affinity propagation algorithm on CRISPR profiles clustering

Affinity Propagation is a message-passing algorithm that considers clustering as a problem of minimizing an "energy" function of the clusters configuration in the data set (see Methods section for a general review of the algorithm, and [8]). This approach seems particularly promising and could help solving species delineations in asexual lineages where obligate gene exchange cannot be used as a delineation criterion [9]. One of the main features of the algorithm is the possibility of regulating the total number of clusters as a function of an input parameter of the algorithm (called the "chemical potential" μ, by analogy to the chemical potential of physical systems, or p for penalty parameter, see Methods). Also the high speed (the computational time goes as N^2 ^if N is the size of the dataset) and thus the possibility to analyze very large networks is encouraging the use of this algorithm. With this method we identified both families and subfamilies in MTC. A single family out of 14 made no sense (Beijing-africanum). This is likely due to a lack of information in Beijing spoligotype pattern as the large 1-36 deletion limits the recognition of other signatures. When considering patterns carrying a larger number of spacers, the classification was largely congruent with the literature. In addition, we could identify new signatures, especially one, termed SEA1, among previously unclassified patterns. We therefore believe that this algorithm is very useful for classifying the widely used 43-spoligotype patterns in *M. tuberculosis *but could prove even more useful on patterns larger than 43 spacers, e.g. the improved 68 spacers format.

### "Euro-american" lineage evolution

Despite large sequencing efforts [25,47], there has been a standing difficulty in unraveling the relationships inside "Euro-American" lineage strains (carrying the 33-36 deletion in the spoligotype pattern), especially in the so-called "T family" described in SpolDB4 [23]. Here, using SpolDB4 database, we could challenge expert-defined families and subfamilies. We first confirmed the validity of S and T2 subfamilies that we suggest to consider as families. The S family was first described in Sicily [48] and independently identified in Québec where a sublineage was shown to harbor a peculiar *pncA *SNP [49]. The T2 family, defined by the absence of spacer 40 was originally described as *M. africanum *2, however was shown later to be a bona fide *M. tuberculosis *subfamily [50]. We also confirmed the reliability of Haarlem family subclustering, if renaming H4 as Ural, and suggest considering H1-2 and H3 as two families. We confirmed the validity of T3 and T5 families as well as T4-CEU (although T4 alone was invalidated). Some LAM subfamilies renamings based on VNTR and SNP loci [34-36] are given further support (LAM7 as TUR, LAM10 as Cameroon), while other were merged (LAM1, 2 and 5). The tendency to merge many expert-defined families was not pervasive. Indeed in the EAI family, four subfamilies out of the 5 expert-defined ones were confirmed.

**Figure 6:**
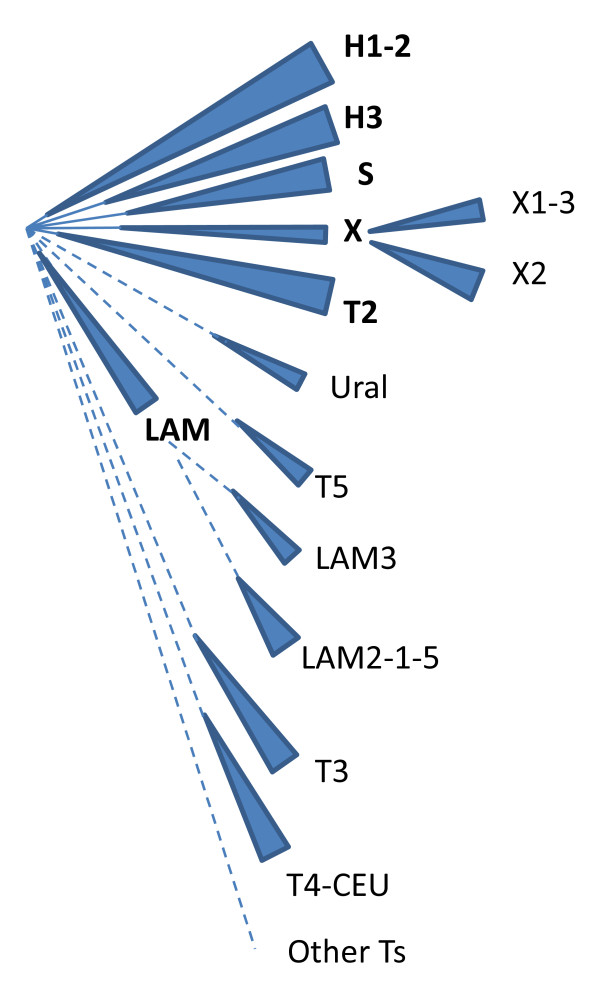
**Evolutionary scheme of the Euro-American supported sublineages**. Note that our study does not identify the monophyly of H1-H2 and H3. Monophyly of T sublineages is not supported by this method either. LAM monophyly (once LAM7 and LAM10 were extracted) is in contrast well supported.

Combining the families and subfamilies identification, we could provide a simplified evolutionary scheme for this lineage (Figure 6). We hope in the future, by applying affinity propagation on 68 spoligotype patterns, to identify other Euro-American subclusters.

## Conclusion

This study describes 1) a novel distance method to be applied on genetic loci evolving by deletion, as for instance do inactive CRISPR loci, 2) a framework to take advantage of identified references for classifying individuals using such loci, 3) a way to identify new references using the Affinity Propagation algorithm [8], and 4) assignations and assignation tools for *M. tuberculosis *complex. The distance method and the framework to identify known references were largely validated by worldwide *M. tuberculosis *database at the CRISPR locus (spoligotype patterns). This work encourages the use of CRISPR patterns to cluster strains in other organisms carrying such loci and for which wide genotyping has been undertaken as it is now the case for human pathogens such as *Yersinia pestis*, *Salmonella enterica*, *Bacilllus anthracis*, *Mycobacterium leprae and M. ulcerans*. Affinity propagation could also be useful to cluster other genotyping data such as SNPs or minisatellites. Databases larger than those available by now are however required to test the validity of this method on such markers.

## Methods

### Spoligotyping data

SpolDB4[33], containing 1939 shared international spoligotypes (SIT) was used as raw data for spoligotyping diversity analysis. This database contains family or subfamily information, with some uncertainties indicated such as LAM3 and S-convergent or T1-T2. To simplify the analyses, when two assignations were provided, only the first one was kept. We also merged certain families when the number of spoligotype patterns was very small and not been confirmed by SNP typing [40]. Specifically, the families we retained are: africanum (n = 46), animal strains (grouping BOVIS, PINNIPEDII, MICROTII, CAPRAE, n_tot _= 231), Beijing (n = 21), CAS (n = 86), EAI (n = 213), H (n = 233), LAM (n = 224), MANU (n = 39), T (n = 482) including S and H37Rv ST as suggested by Brudey et al (2006), X (n = 90). We excluded SIT69 which was suppressed by Institut Pasteur [33] as well as the canetti spoligotype pattern which is unique (SIT592). There are 272 unclassified spoligotypes (U). The references for each family correspond to SpolDB4 description [23]; they are listed in Table 1.

### Methods to compute distances

Three new methods to compute distances were designed that fit CRISPR loci evolutionary dynamics such as that of *M. tuberculosis *complex *i.e. *evolution by deletion or transposon insertion. All methods rely on the identification of the beginning and the end of spacers deletions. These limits were named Domain Walls (Figure 1). The "Domain Walls" method measures the proportion of common Domain Walls between two CRISPR profiles, *i.e. *if the profiles have i_w _and j_w _Domain Walls respectively and K_w _are common, the distance is:

The "Blocks" method considers blocks of spacers; let i_b _and j_b _be the numbers of blocks carried by the two profiles and K_b _the number of blocks they share,

The "Deletions" method considers deleted blocks; let i_d _and j_d _be the numbers of blocks of deletions carried by the two profiles and K_d _the number of shared deletions,

These distance methods were used to compute the distance between each SpolDB4 spoligotype pattern and the references of the ten main *M. tuberculosis *complex families. The scripts for such calculations were written in R language [51] and are available upon request. Each pattern was assigned to the family whose reference it was the most similar to.

### Clustering algorithms

Affinity Propagation (AP), proposed first in [8], is a recent clustering method based on the choice of "exemplars" as centers of the clusters, i.e., one representative data point for each cluster to which the other nodes rely. The choice of the exemplars is based on the minimization of the total "energy" of the system, function of the total distance between data points and exemplars in a given clusters configuration. This method falls in the class of message-passing type algorithms, exploiting the Belief Propagation method (also known as Cavity method in the physics literature) to minimize the energy function in an computationally efficient way (from the exponential time complexity of the naïve methods to O(N^2^), where N is the total number of nodes to cluster). The starting point is thus a set of data points, representing the nodes of the network, and a similarity matrix S defining the similarities among all the nodes as deduced from the distance between all these nodes. The similarity between two points *i *and *j *is defined as

provided that the distance *d *ranges from 0 to 1, as in our case (this is always true up to a normalization of the distance). The aim is then to find a map *c *:{1,..., *N*} →{1,..., *N*}, with *N *being the total number of data points and *c*(*i*) ≡ *c_i _*is the exemplar of node *i*, such that the vector  minimizes the energy function

The first term of the function defined above is (minus) the sum of all the similarities between a point and its exemplar, while the second term is introduced to avoid any configuration in which an exemplar does not belong to the cluster that itself represents, that is, an exemplar must be the exemplar of itself. This is granted by defining the function  as

and by taking the log function of it and summing it over all the nodes, so that the energy becomes infinite if at least an exemplar is represented by a different exemplar. The parameter *β *plays formally the role of the inverse of the temperature in a thermodynamical system, and thus determines the level of thermal noise acting on the system. This means that varying this parameter, but keeping it finite, allows the algorithm to accept configurations of the clusters that do not exactly corresponds to minima of the energy function. We consider here only the optimal case of zero temperature, *i.e*., *β *→ ∞ (for a general and exhaustive treatment of the cavity method see, for example [52]).

Once the Cavity equations are written one is left with two coupled update rules for each couple of nodes *(i, j)*:

These update rules represents messages that the nodes are exchanging between iteration *t *and *t+1*, with  and  representing respectively the energetic "competition" between node *i *and all the other nodes except *j *to be the exemplar of node *j *and the gain in the total energy of the system if node *j *is represented by node *i*. The notation *i *→ *j *indicates that the message is sent from node *i *toward node *j*. When the update equations converge, then the value of each *c_i_*, *i *= 1,..., *N*, is obtained summing over all the messages arriving at node *i *and maximizing the sum. The diagonal elements of the similarity matrix, that are not constrained to be equal to the unity, play the role of an effective cost to every chosen exemplar, and thus a cost for the total number of clusters found. They are a fine-tuning for the selection of the total number of clusters found by AP. In our study we chose to consider every data point *a priori *equally probable to be an exemplar, so we set *S*(*i*,*i*) = *p *∀ *i *= 1,..., *N*. Varying the parameter *p *from very large (negative) values up to positive values gives the range of total clusters from 1 to N, and we interpret a stability in the total number of clusters under changes of this parameter as a genetically meaningful grouping of the data, as discussed in the Results section. The similarity matrix S was obtained using the "Deletions" distance that had turned out to be the most accurate distance. Linear combinations of the various distances introduced in the previous section were also considered, but the overall result still favors the Deletions distance. We performed also a comparison of AP with other "classical" clustering algorithms, such as K-Means and Hierarchical clustering. We considered the performance with respect to the experts' classification as defined in SpolDB4 [23,33] and identified that AP found clusters with much lower error (see Additional File 3). The script for computing the distance matrices of SpolDB4 database and performing the analysis with AP was written in Matlab as a self-contained script, the bare AP algorithm for Matlab is available from the authors Frey and Dueck.

## Authors' contributions

CS and SF initiated this work through informal discussions; ML did the first experiments under SF supervision and CS guidance for classification (University Paris-Sud Master 1 program, Physics). ML wrote his Master report on this topic. SF supervised CB (Master 2 program) to program writing, acquisition and analysis of data. GR performed complementary program writing, data acquisition and analysis. CB and GR were both involved in data analysis and writing of the manuscript. CS provided the taxonomic expertise and contributed to the revision of the manuscript. All authors approved the final version.

## Supplementary Material

Additional file 1 **Plot of similarity to their reference for patterns assigned as the expert classification (Green), patterns not assigned due to ambiguity (Gray) and patterns assigned differently than the expert classification (Red)**. Click here for file

Additional file 2 **SpolDB4 new assignations, using the previously identified references or the newly identified ones**. Click here for file

Additional file 3 **Mean similarity of patterns with their representative as a function of the cluster size, and for different clustering methods (AP: Affinity Propagation; Bio: Bionumerics; KM: K-Means)**. Click here for file
