# Supplementary figures and images for: Using affinity propagation for identifying subspecies among clonal organisms: lessons from M. tuberculosis
Source: BMC Bioinformatics. 2011 Jun 2;12:224. doi: 10.1186/1471-2105-12-224 (PMC3126747; doi:10.1186/1471-2105-12-224)

## Additional file 1

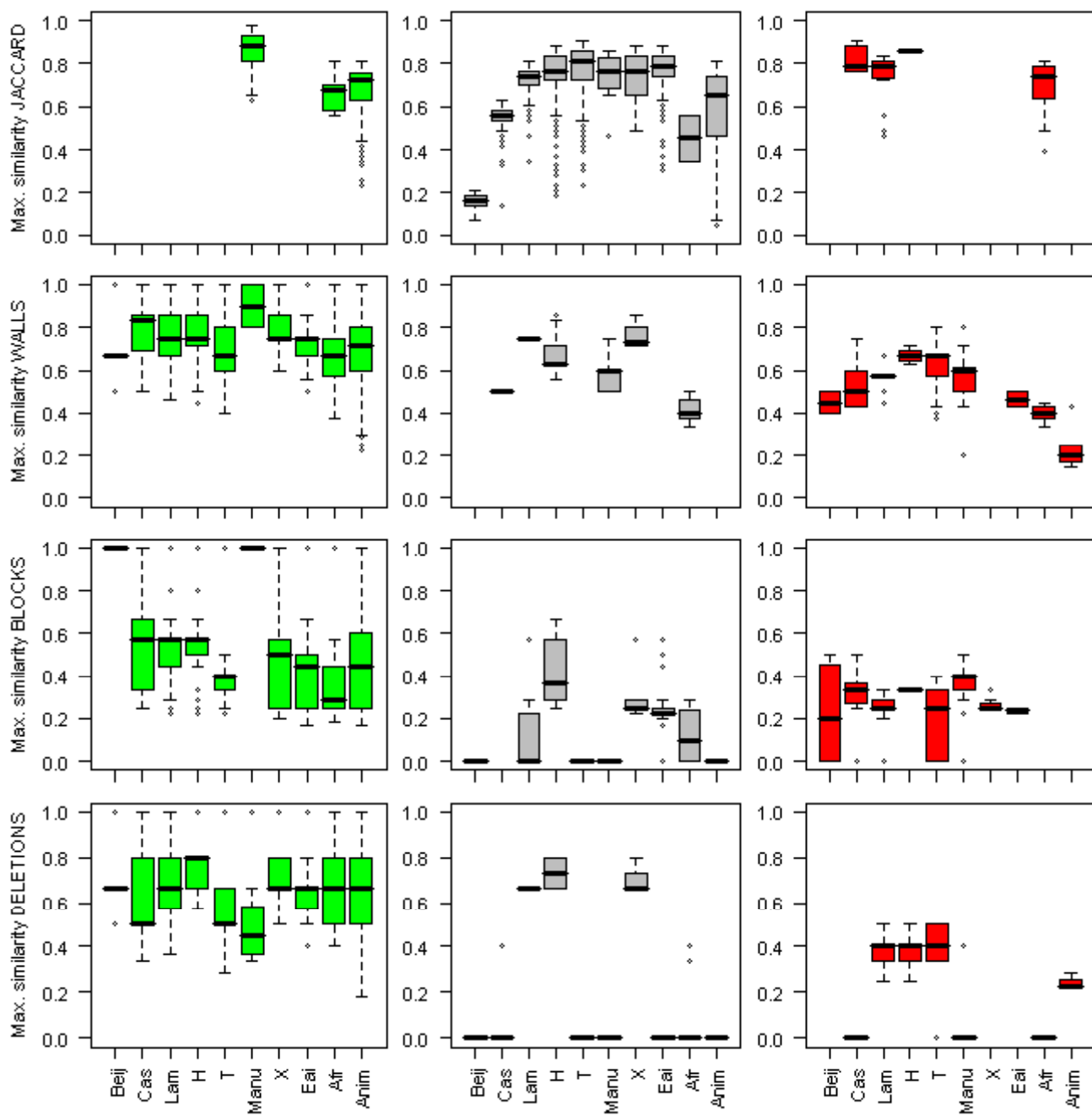

Supplement: Additional file 1 — Plot of similarity to their reference for patterns assigned as the expert classification (Green), patterns not assigned due to ambiguity (Gray) and patterns assigned differently than the expert classification (Red). [file 1471-2105-12-224-S1.PDF]

### Additional file 3

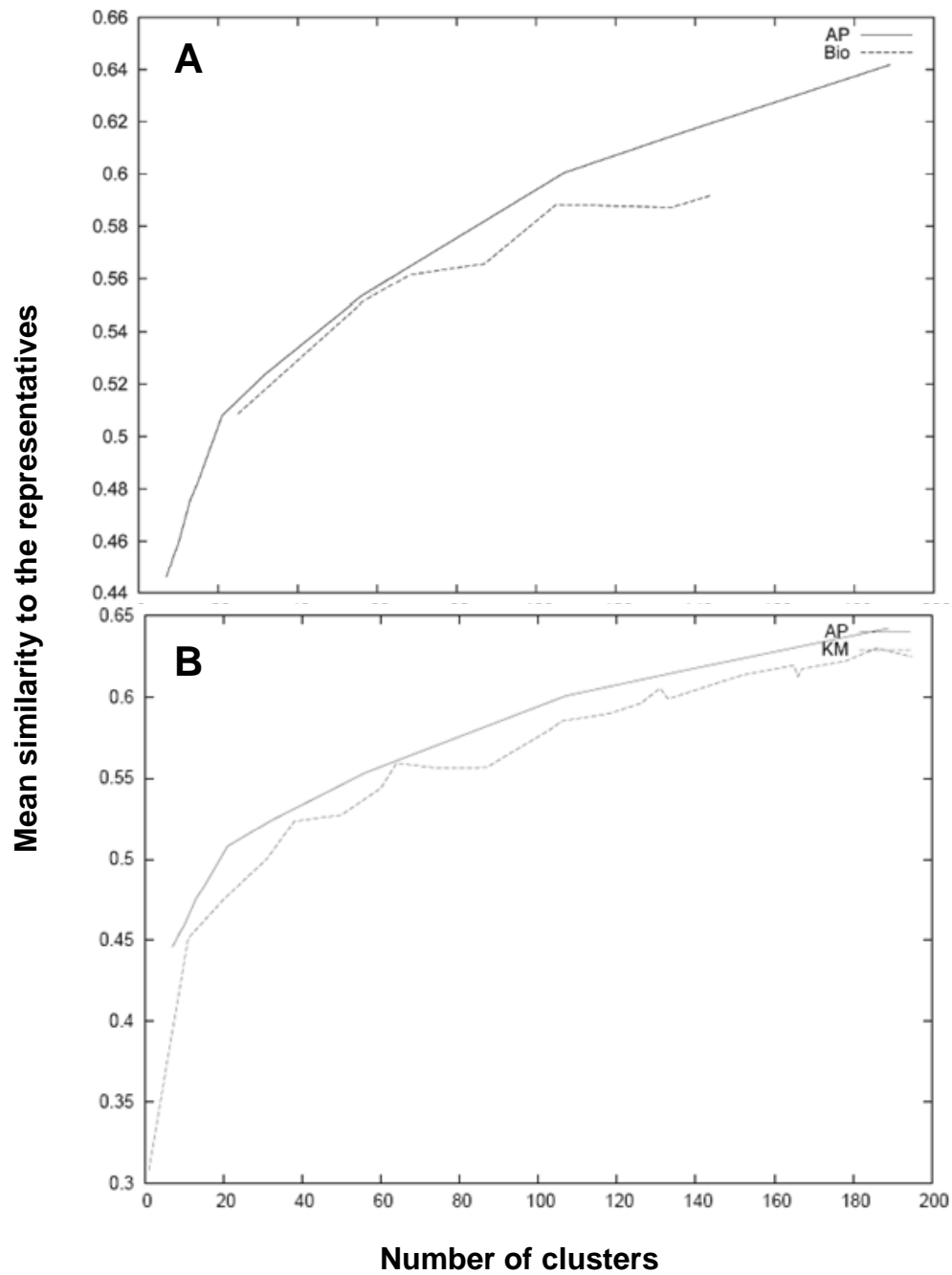

Supplement: Additional file 3 — Mean similarity of patterns with their representative as a function of the cluster size, and for different clustering methods (AP: Affinity Propagation; Bio: Bionumerics; KM: K-Means). [file 1471-2105-12-224-S3.PDF]
