# Supplementary material for: Using affinity propagation for identifying subspecies among clonal organisms: lessons from M. tuberculosis
Source: BMC Bioinformatics. 2011 Jun 2;12:224. doi: 10.1186/1471-2105-12-224 (PMC3126747; doi:10.1186/1471-2105-12-224)
Supplement: Additional file 2 — SpolDB4 new assignations, using the previously identified references or the newly identified ones. [file 1471-2105-12-224-S2.PDF]

Additional File 2

SIT	spoligotype pattern	SpolDB4 family	SpolDB4 subfamily	Deletion-based family using SpolDB references
1	□□□□□□□□□□□□□□□□□□□□□□□□□□□□□□□□□□■■■■■■■■■	BEIJ	BEIJ	BEIJ
2	□□□□□□□□□□□□□□□□□□□□□□□□■□□□□□□■□□□□■■■■■■■	H	H2	T
3	□□□□□□□□□□□□□□□□□□□□□□□□■■■■■■□■□□□□■■■■■■■	H	H3	H
4	□□□□□□□□□□□□□□□□□□□□□□□□■■■■■■■■□□□□■■■■■■■	LAM	LAM3	T
5	□□□□□□□□□■■□■■■■■■■■■■■■■■■■■■■■□□□□■■■■■■■	T	T1	T
6	□□□■■■■■■■■■■■■■■■■■■■■■■■■■□□□□■□■■■■■□■■■	EAI	EAI1	EAI
7	□■■■■■■■■■■■■■■■■■■■■■■■■■■■■■■■□□□□■■■■■■■	T	T1	T
8	■□□□□□□□□□□□□■■■■■■■■■■■■■■■□□□□■□■■■■■■■■■	EAI	EAI5	EAI
9	■□□□□□□□■■■■■■■■■□■■■■■■■■■■■■■■■■■■■■■■■■□□□□■■□□□□■	X	X2	X
10	■□□■■■■■■■■■■■■■■■□■□■■■■■■■□□□□■□■■■■■■■■■	EAI	EAI5	EAI
11	■□□■■■■■■■■■■■■■■■■■■■■■■■■■□□□□■□■■□□□■■■■	EAI	EAI2	EAI
12	■□■□□□□□□□□□□■■■■□■■■■■■■■■■■■■■□□□□■■■■■■■	X	X3	X
13	■□■□□□□□□□□□■■□■■□■■■■■■■■■■■■■■□□□□■■■■■■■	X	X3	X
14	■□■□□□□□□□□□■■■■■□■■■■■■■■■■■■■■□□□□■■■■■■■	X	X3	X
15	■□■■■■■■■■■■■■■■■■■■□□□□□□□□■■■■□□□□■■■■■■■	T	T1	T
16	■□■■■■■■■■■■■■■■■■■■■■■■■■■■□□□□■□■■□□□■■■■	EAI	EAI5	EAI
17	■■□■■■■■■■■■□■■■■■■■□□□□■■■■■■■■□□□□■■■■■■■	LAM	LAM2	LAM
18	■■□■■■■■■■■■■■■■■□■■■■■■■■■■■■■■□□□□■■□□□□■	X	X2	X
19	■■□■■■■■■■■■■■■■■■■□□■■■■■■■□□□□■□■■■■■■■■■	EAI	EAI2	EAI
20	■■□■■■■■■■■■■■■■■■■■□□□□■■■■■■■■□□□□■■■■■■■	LAM	LAM1	LAM
21	■■■□□□□■■□■■■■■■■■■□□□□□□□□□□□□□□□□■■■■■■■■	CAS	CAS1	CAS
22	■■■□□□□■■■■■■■■■■■■□□□□□□□□□□□□□□□□■■■■■■■■	CAS	CAS	CAS
23	■■■□□□□■■■■■■■■■■■■■■■□□□□□□□□□□□□□■■□□■■■■	CAS	CAS	CAS
24	■■■□□□□■■■■■■■■■■■■■■■□□□□□□□□□□□□■■□□□□■■■	CAS	CAS1	CAS
25	■■■□□□□■■■■■■■■■■■■■■■□□□□□□□□□□□□■■□□■■■■■	CAS	CAS1	CAS
26	■■■□□□□■■■■■■■■■■■■■■■□□□□□□□□□□□□■■■■■■■■■	CAS	CAS1	CAS
27	■■■□□□□■■■■■■■■■■■■■■■□□■■■■■■■■■□□□□■■■■■■	U	U	CAS
28	■■■□■■■■■■■■■■■■■■■■■■■■■■■■□□□□■□■■□■■■■■■	EAI	EAI5	EAI
29	■■■■■□□□□□□□□□□□□■■□□□□□□□□□□□□□□□□□□□■■■■■	U	U	Unassignable
30	■■■■■■□■■■■■■■■■■□■■□□□□■■■■■■■■□□□□■■■■■■■	LAM	LAM9	Unassignable
31	■■■■■■■□□□□□□■■■■■■■■■■■■■■■■■■■□□□□■■■□□□□	T	T1	T
32	■■■■■■■■□□□□□□□□□□□□□□□□□□□□□□□□□□□□□□■■■■■	U	U	Unassignable
33	■■■■■■■■□□□■■■■■■■■■□□□□■■■■■■■■□□□□■■■■■■■	LAM	LAM3	LAM
34	■■■■■■■■□□■■■■■■■■■■■■■■■■■■■■■■□□□□■■■■■■■	T	S	T
35	■■■■■■■■■■■■□■■■■■■■■■■■■■■■□□□■□□□□■■■■■■■	H	H4	T
36	■■■■■■■■■■■■□■■■■■■■■■■■■■■■■□■□□□□■■■■■■■	H	H3	H
37	■■■■■■■■■■■■□■■■■■■■■■■■■■■■■■■■□□□□■■■■■■■	T	T3	T
38	■■■■■■■■■■■■■■■■■□■□□□□□■■■■■■■■□□□□■■□□□□■	X	X2	X
39	■■■■■■■■■■■■■■■■■■□■■■□□■■■■■■■■□□□□■□□■■■■	T	T4	T
40	■■■■■■■■■■■■■■■■■■□■■■■■■■■■■■■■□□□□■■■■■■■	T	T4	T
41	■■■■■■■■■■■■■■■■■■■□□□□□■□□■■■■■□□□□■■■■■■■	LAM	LAM7	T
42	■■■■■■■■■■■■■■■■■■■■□□□□■■■■■■■■□□□□■■■■■■■	LAM	LAM9	LAM
43	■■■■■■■■■■■■■■■■■■■■■■□□■■■■□□□□■□■■■■■■■■■	EAI	EAI6	EAI
44	■■■■■■■■■■■■■■■■■■■■■■□■■■■■■■■■□□□□■■■■■■■	T	T5	T
45	■■■■■■■■■■■■■■■■■■■■■■■□■□□□□□□■□□□□■■■■■■■	H	H1	T
46	■■■■■■■■■■■■■■■■■■■■■■■■□□□□□□□□□□□□□□□□□□□	U	U	Unassignable
47	■■■■■■■■■■■■■■■■■■■■■■■■■□□□□□□■□□□□■■■■■■■	H	H1	T
48	■■■■■■■■■■■■■■■■■■■■■■■■■■■■□□□□■□■■■■■□■■■	EAI	EAI1	EAI
49	■■■■■■■■■■■■■■■■■■■■■■■■■■■■■■□■□□□□■■■□■■■	H	H3	H
50	■■■■■■■■■■■■■■■■■■■■■■■■■■■■■■□■□□□□■■■■■■■	H	H3	H
51	■■■■■■■■■■■■■■■■■■■■■■■■■■■■■■■■□□□□■■■□□□□	T	T1	T
52	■■■■■■■■■■■■■■■■■■■■■■■■■■■■■■■■□□□□■■■□■■■	T	T2	T

53	■■■■■■■■■■■■■■■■■■■■■■■■■■■■■■■■□□□□■■■■■■■	T	T1	T
54	■■■■■■■■■■■■■■■■■■■■■■■■■■■■■■■■□□■■■■■■■■■	MANU	MANU2	Unassignable
55	■■■■■■■■□□■■■■■■■■■■■■■■■□■■□■■□□□□□■■■■■■■	U	U	Unassignable
56	■■■■■■■■■■■■□■■■■■■■■■■□□□□□□□□□□□□□□□□□□□	U	U	Unassignable
57	■■■■■■■■■■■■■■■■■■□□■■□□□■■■■■■■□□□□■■■■■■■	LAM	LAM10	T
58	■■■■■■■■■■■■■■■■■■■□■■□■■■■■■■■■□□□□■■■■■■■	T	T5	T
59	■■■■■■■■■■■■■■■■■■■■□□□□■■□□□□■■□□□□■■■■■■■	LAM	LAM11	LAM
60	■■■■■■■■■■■■■■■■■■■■□□□□■■■■■■■■□□□□■■■□■■■	LAM	LAM4	LAM
61	■■■■■■■■■■■■■■■■■■■■■■□□□■■■■■■■□□□□■■■■■■■	LAM	LAM10	T
62	■■■■■■■■■■■■■■■■■■■■■■■■■□□□□□□■□□□□■■■□■■■	H	H1	T
63	□■■■■■■■■■■■■■■■■■■■■□■■■■■■■■■■□□□□■■■■■■■	T	T1	T
64	■■■■■■■■■■■■■■■■■■■■□□□□■■■■□■■■□□□□■■■■■■■	LAM	LAM6	LAM
65	■■■■■■■■■■■■■■■■■■■■■■■■■■■■■■■■□□□□■□□■■■■	T	T1	T
66	■■■■■■■■■■■■■■■■■■□□□□■■■■■■■■□■□□□□■■□■■■■	H	H3	H
67	■■■■■■■■■■■■■■■■■■□□□□■■■■■■■■□■□□□□■■■■■■■	H	H3	H
68	■■■■■■■□■■■■■■■■■■■■■■□■■■■■■■■■□□□□■■■■■■■	T	T5	T
70	■■■□□□□□□□□□■■■■■□■■■■■■■■■■■■■■□□□□■■□■■■■	X	X3	X
71	■■■■■■■■□□■■□■■■■■■■■■■■■■■■■■■■□□□□■■■■■■■	T	S	T
72	■■■■■■■■■■□■■■■■■■■■■■■■■■■□□□□□□□■■■■■□■■■	EAI	EAI	Unassignable
73	■■■■■■■■■■■■□■■■■■■■■■■■■■■■■■■■□□□□■■■□■■■	T	T2	T
74	■■■■■■■■■■■■■■■■■■■■□□□□□□□□□■■■□□□□■■■■■■■	T	T1	T
75	■■■■■■■■■■■■■■□■■■■■■■■■■■■■■■□■□□□□■■■■■■■	H	H3	H
76	■□□■■■■□□□□□□□□□□■□■■■■■■■■■□□□□■□■■■■■■■■■	EAI	EAI5	EAI
77	■■■■□□□■■■■■■■■■■■■■■■■■■■■■■■■■□□□□■■■■■■■	T	T1	T
78	■■■■■■■■■■■■■■■■■■■■■■■■■■■■■■■■□□□□■■■□□■■	T	T1	T
79	■□■□□■■■■□■■■□□■■■■■□□□□■■□□□□■■□□□□■■□■■□■	LAM	LAM11	LAM
80	■■■■■□■■■■■■■■■■■■■■■■□□■□□□□□□■□□□□■■■■■■■	H	H1	T
81	■■■■■■■■■□■■■■■■■■■■□□□□■■■■■■■■□□□□■■■■■■■	LAM	LAM9	LAM
82	■■■■■■■■■■■■■□■■■■■■□□□□■■□□□□■■□□□□■■■□■■■	LAM	LAM11	LAM
83	■■■■■■■■■■■■■□■■■■■■■■■■■■■■■■■■□□□□■■□■■■■	T	T1	T
84	■■■■■■■■■■■■■■■□■■■■□□□□■□□□□□■■□□□□■■■□■■■	LAM	LAM11	LAM
85	■□■■□■■■■■■■■□■■■■■■□□□□■■□□□□■■□□□□■■■■■■■	LAM	LAM11	LAM
86	■■■■■■■■■■■■■■■■■■■■■□■■■■■■■■■■□□□□■■■■■■■	T	T1	T
87	■□■■■■■■■■■■■□■■■■■■□□□□■■□□□□■■□□□□■■■■■■■	LAM	LAM11	LAM
88	■■■■■■■■□□■■□■■■■■■■■■■■■■■■■■■■□□□□■■■□■■■	T	S	T
89	■■□■■■■□□□□□□□□□□□□□□□□□□■■■□□□□■□■■■■■■■■■	EAI	EAI2	EAI
90	■■□■■■■■■■■■■■■■■■■■■■■■■□□□□□□□□□□□■■■■■■	U	U	animal
91	■■■□□□□□□□□□□■■■■□■■■■■■■■■■■■■■□□□□■■■■■■■	X	X3	X
92	■■■□□□□□□□□□■■■■■□■■■■■■■■■■■■■■□□□□■■■■■■■	X	X3	X
93	■■■■■■■■■■■■□■■■■■■■□□□□■■■■■■■■□□□□■■■■■■■	LAM	LAM5	LAM
94	■■■■■■■■■■■■■■■■■■□■■■□□■■■■□■■■□□□□■□□■■■■	T	T4	T
95	■■■■■■■■■■■■■■■■■■■■□□□□■■■■□■■■□□□□■■■□■■■	LAM	LAM6	LAM
96	■■■■■■■■■■■■■■■■■■■■■■□■□□□□□□□□□□■■□■■■■■■	EAI	EAI7	Unassignable
97	■■■■■■■■■■■■■■■■■■■■■■■□□□□■■■■■□□□□■■■■■■■	T	T1	T
98	□□■■■■□□□□□□□□□□■■■■□□□□■■■■■■■■□□□□■■■■■■■	LAM	LAM9	LAM
99	■■■■□■■■■■■■■■■■■■■■■■■■■■■■■■□■□□□□■■■■■■■	H	H3	H
100	■■■■■■■■■■■■■■■■■■■■■■■■■■■■■■■■■□■■■■■■■■■	MANU	MANU1	MANU
101	■■■■■■■□□□□□■■■■■■■■□□□□■■□■■■■■□■■■□□□■■■■	AFRI	AFRI2	AFRI
102	■■■■■■■■■■■■□□□□■■■■■■■■■■■■■■■■□□□□■■■■■■■	T	T1	T
103	■■■■■■■■■■■■■■■■■■■■■■□■■□□□□□□■□□□□■■■□■■■	H	H1	T
104	■□■■■■■■□□□■■■□■■■■□□□□□□□□□□□□□□□□□□□■■■■■	U	U	Unassignable
105	■■■■■■■■□□□■■■□□□□□□□□□□□□□□□□□□□□□□□□□■■■■	U	U	Unassignable
106	■■■■■■■■□□□■■■■■■■■□□□□□□□□□□□□□□□□□□□■■■■■	U	U	Unassignable
107	□□□□■■■■□□■■■■■■■■■■■■■■■■■■■■■■□□□□■■■■■■■	T	S	T
108	□■■■■■■■■■■■■■■■■■■■■■■■■■■■■■□■□□□□■■■□■■■	H	H3	H
109	■□□■■■■■■■■■■■■■■■□■■■■■■■■■□□□□■□■■■■■■■■■	EAI	EAI8	EAI

110	■■■■■■□□□■■■■■■■■■■■■■■■■■□■■■■□■□□□□■■■■■■■	H	H3	H
111	■■■■■■■■□□□■■■□■■■■■□□□□■■■■■■■■□□□□■■■■■■■	LAM	LAM3	LAM
112	■■■■■■■■■■■■□■■■■■■■■■■□□□■■■■■■□□□□■■■■■■■	T	T3	T
113	■□□■■■■■■■■■■■■■■■■■■■■■■■□■□□□□■□■■□□□■■■■	EAI	EAI3	EAI
114	■■■■■■■■■■■■■■□■■■■■■■□□□□■■■■■■□□□□■■■■■■■	T	T1	T
115	■■■■■■■■■■■■■■□■■■■■■■□□□■■■■■■■□□□□■■■■■■■	LAM	LAM10	T
116	■■■■■■■■■■■■■■□■■■■■■■■■■□■■■■□■□□□□■■■■■■■	H	H3	H
117	■■■■■■■■■■■■■■□■■■■■■■■■■■■■■■■■□□□□■■■□■■■	T	T1	T
118	■■■■■■■■■■■■■■□■■■■■■■■■■■■■■■■■□□□□■■■■■■■	T	T2	T
119	■■■■■■■■■■■■■■■■■□■■■■■■■■■■■■■■□□□□■■■■■■■	X	X1	X
120	■■■■■■■■■■■■■■■■■■■□■■■■■■■■■■■■□□□□■■■■■■■	T	T1	T
121	■■■■■■■■■■■■■■■■■■■■■■■■■□■■■■□■□□□□■■■■■■■	H	H3	H
122	■■■■■■■■■■■■■■■■■■■■■■■■■□■■■■■■□□□□■■■■■■■	T	T1	T
123	■■■■■■■■■■■■■■■■■■■■■■■■■■□□■■■■□□□□■■■■■■■	T	T1	T
124	■■■■■■■■■■■■■■■■■■■■■■■■■■■■■■□□□□□□■■■■■■■	U	U	Unassignable
125	□□□□□□□□□□□□□□□□□□□□□□□□■■■■■■■■□□□□■■■□■■■	T	T2	T
126	■□□■■■■■■■■■■■■■■■■■■■■■■■■■□□□□■□■■■■■■■■■	EAI	EAI5	EAI
127	■□■■■■■■■■■■■■■■■■■■■■■■■■■■□□□■□□□□■■■■■■■	LAM	LAM3	T
128	■■□□■■■■■■■■■■■■□■■■■■■■■■■■■■■■□□□□■■■□■■□	T	T1	T
129	■■■□□□□□□■■■■■■■■■■■■■□□■■■■□□□□■□■■■■■■■■■	U	U	EAI
130	■■■■■■■■□□□■■■■■■■■■□□□□■■■■■■■■□□□□■■■□■■■	T	T1	LAM
131	■■■■■■■■■■■■□□■■■■■■■■■■■■■■■■■■□□□□■■■■■■■	H	H3	T
132	■■■■■■■■■■■■■■■■■■■■□□□□■■□□□□□□□□□□□□□□■■■	T	T2	LAM
133	■■■■■■■■■■■□□□□□■■■□□□□□■□■■■■■■□□□□■□□□■■□	T	T1	T
134	■■■■■■■■■■■■■■■■■■■■■■■■■■■■■■□■□□□□■■□□■■■	H	H3	H
135	■■■■■■■■■■■■■■■■■■■■■■■■■■■■■■■■□□□□■■■□■■□	T	T2	T
136	■■■■■■■■■■■□□□□□■■■□□□□□■□■■■■■■□□□□■□□■■■■	T	T1	T
137	■■■■■■■■■■■■■■■■■□■■■■■■■■■■■■■■□□□□■■□□□□■	X	X2	X
138	■■■■■■■■■■■■■■■■■■■■■■■■■■■■□□□□■□■■■■■□□□□	EAI	EAI5	EAI
139	■■■■■■■■■■■■■■■■■■■■■■■■■□□■□□□□■□■■■■■■■■■	EAI	EAI4	EAI
140	■■■□□□□■□■■■■■■■■■■■■■□□□□□□□□□□□□■■□□■■■■■	CAS	CAS1	CAS
141	■■■□□□□■■■■■■■□■■■■■■■□□□□□□□□□□□□■■■■■■■■■	CAS	CAS1	CAS
142	■■■□□□□■■■■■■■■■■■■■■□□□□□□□□□□□□□■■■■■■■■■	CAS	CAS	CAS
143	■■■■□■■■■■□□□□□□□□□□□□□□■□□□□□□■□□□□■■■■■■■	H	H1	T
144	■■■■■■□□□□□□□□□□□□□□□□□□□■■■■■■■□□□□■■■■■■■	T	T1	T
145	■■■■■■□□□□□□□□□■■■□□□□□□□□□□□□□□□□■■□□□■■■■	AFRI	AFRI	AFRI
146	■■■■■■■□□□□□□□□□□□□□□■■■■■■■■■■■■■■■■□□□□■■	AFRI	AFRI	Unassignable
147	■■■■■■■□□■■■■■■■■■■■□□□■■■□□□□□□□□□□□□■■■■■	U	U	Unassignable
148	■■■■■■■□□■■■■■■■■■■■■□□■■■□□□□□□□□□□□□■■■■■	U	U	Unassignable
149	■■■■■■■■■□□□□□□□□□□■■■■■■■■■■■■■□□□□■■■■■■■	T	T3	T
150	■■■■■■■■■■■■■■□■■■■■□□□□■■■■■■■■□□□□■■■■■■■	LAM	LAM9	LAM
151	■■■■■■■■■■■■■■□■■■■■■■■■■□□□□□□■□□□□■■■■■■■	H	H1	T
152	■■■■■■■■■■■■■■■■■■■■■■■■■■□■□□□□■□■■■■■■■■■	EAI	EAI5	EAI
153	■■■■□■■■■■■■■■■■■■■■■■■■■■■■■■■■□□□□■■■□■■■	T	T2	T
154	■■■■□■■■■■■■■■■■■■■■■■■■■■■■■■■■□□□□■■■■■■■	T	T1	T
155	■■■■■■■■□□□■■□■■■■■■■■■■■■■■■■■■□□□□■■■■■■■	T	T1	T
156	■■■■■■■■□□□■■■■■■■■■■■■■■■■■■■■■□□□□■■■■■■■	T	T1	T
157	■■■■■■■■■■■■□■■■■■■■■■■■■■■■■■■■□□□□■□□■■■■	T	T3	T
158	■■■■■■■■■■■■□■■■■■■■■■■■■■■■■■■■□□□□■■□□□□■	T	T3	T
159	■■■■■■■■■■■■■□□□□□□□□□□■■■■■■■■■□□□□■■■■■■■	T	T1	T
160	■■■■■■■■■■■■■■■■■■■□□■■■■■■■□□□□□□□□□□□□□□■	U	U	Unassignable
161	■■■■■■■■■■■■■■■■■■■■□□□□■■■■■■■□□□□□■■■■■■■	U	U	LAM
162	■■■■■■■■■■■■■■■■■■■■□□□□■■■■■■■■□□□□■■□■■■■	LAM	LAM9	LAM
163	■■■■■■■■■■■■■■■■■■■■□□□□■■■■■■■■□□□□■■■□□□□	LAM	LAM9	LAM
164	■■■■■■■■■■■■■■■■■■■■■■■□□□□□□□■■□□□□■■■■■■■	T	T1	T
165	■■■■■■■■■■■■■■■■■■■■■■■■■□□■■■□■□□□□■■■□■■■	H	H3	H

166	■■■■■■■■■■■■■■■■■■■■■■■■■■■□■■□■□□□□■□■■■■■	H	H3	H
167	■■■■■■■■■■■■■■■■■■■■■■■■■■■■■□■■□□□□■■■■■■■	T	T1	T
168	■■■■■■■■■■■■■■■■■■■■■■■■■■■■■■□■□□□□■■□■■■■	H	H3	H
169	■■■■■■■■■■■■■■■■■■■■■■■■■■■■■■■□□□□□■■■□□□□	U	U	Unassignable
170	■■■■■■■■□□□□□□■■■■■■■■■■■■■■■■■■□□□□■□□□□□□	T	T1	T
171	■■■■■■■■□□□□□□■■■■■■■■■■■■■■■■■■□□□□■■■■■■■	T	T1	T
172	■■■■■■■■■■■■■■■■■■■■■■■■■■■■■■■□□□□□■■■■■■■	U	U	Unassignable
173	■■■■■■■■■■■■■■□■■■■■□■■■■■■■■■■■□□□□■■■■■■■	T	T1	T
174	■■■■■■■■■■■■■■■■■■□□□□■■■■■■■■■■□□□□■■■■■■■	T	T1	T
175	■■■■■■■■■■■■■■■■■■■■□■■■■■■■■■■■□□□□■■■□■■■	T	T2	T
176	■■■■■■■■■■■■□■■■■■■■□□□□■■■■□■■■□□□□■■■■■■■	LAM	LAM5	LAM
177	□■■■■■■■■■■■■■■■■■■■□□□□■■■■■■■■□□□□■■■■■■■	LAM	LAM9	LAM
178	■□□■■■■■■■■■□■■■■■■■□□□□■■■■■■■■□□□□■■■□■■■	LAM	LAM4	LAM
179	■■□■■■■■■■■■□■■■■■■■□□□□■■■■■■■■□□□□■■■□□■■	LAM	LAM2	LAM
180	■■□■■■■■■■■■■■■■■■■■■■■■■■■■■■□■□□□□■■■■■■■	H	H3	H
181	■■■■■■□□□■■■■■■■■■■■■■■■■■■■■■■■■■■■□■■■■	AFRI	AFRI1	Unassignable
182	■■■■■■■■■■■■■■■■■■□□□□□□■□□□□□□■□□□□■■■■■■■	H	H1	T
183	■■■■■■■■■■■■■■■■■■□■■■■■■■■■■■□■□□□□■■■■■■■	H	H3	H
184	■■■■■■■■■■□□□□■■■■■■□□□□■■□□□□■■□□□□■■■□■■■	LAM	LAM11	LAM
185	■■■■■■■■■■■■■■□■■□■■■■■■■■■■■■■■□□□□■■□□□□■	X	X2	X
186	■■■■■■■■■■■■■■□■■■■□□□□□■□□■■■■■□□□□■■■■■■■	LAM	LAM7	T
187	■■■■■■□□□■■■■■■■■■■■■■■■■■■■■■■□■■■■■□■■■■	AFRI	AFRI1	Unassignable
188	■■■■■■□□□■■■■■■■■■■■■■■■■■■■■■■■■■■■□□■■■	AFRI	AFRI	Unassignable
189	■■■■■■■■■■■■■□□□□■■■■■■■■■■■■■■■□□□□■■■■■■■	T	T1	T
190	□□□□□□□□□□□□□□□□□□□□□□□□□□□□□□□□□□■■■■■□■■■	BEIJ	BEIJ	BEIJ
191	□□■■■■■■■■■■■■■■■■■■■■■■■■■■■■■■□□□□■■■■■■■	T	T1	T
192	■□□□□□□□□□□□□□□□■■■■■■■■■■■■□□□□■□■■■■■■■■■	EAI	EAI5	EAI
193	■■□■■■■■■■■■□■■■■■■■□□□□■■■■■□■■□□□□■■■■■■■	LAM	LAM2	LAM
194	■■□■■■■■■■■■□■■■■■■■□□□□■■■■■■■■□□□□■■■□■■■	LAM	LAM2	LAM
195	■■□■■■■■■■■■■■■■■■■■□□□□■■■■■□■■□□□□■■■■■■■	LAM	LAM1	LAM
196	■■□■■■■■■■■■■■■■■■■■■■■■■■■■■■■■□□□□■■■■■■■	T	T1	T
197	■■■□□□□□□□□□□■■■■□■■■■■■■■■■■■■■□□□□■■■□■■■	X	X3	X
198	■■■□□□□□□□□□■□□□□□■■■■■■■■■■■■■■□□□□■■■■■■■	T	T1	T
199	■■■□□□□□□□□□■■■■■□■■■■■□□□□□□□□□□□□□□□□□□■■	U	U	X
200	■■■□□□□□□□□□■■■■■□■■■■■■■■■■■■■■□□□□■■■□□□□	X	X3	X
201	■■■□□□□□□□■■■■■■■■□□□□□□□□□□□□□□□□■■■■■■■■■	CAS	CAS	Unassignable
202	■■■□□□□□■■■■■■■■■□■■■■■■■■■■■■■■□□□□■■□□□□■	X	X2	X
203	■■■□□□□■■■■■■■■■■■■■■■□□□□□□□□□□□□□■■■■■■■■	CAS	CAS	CAS
204	■■■□■■■■■■■■■■■■■■■■■■■■■■■■□□□□■□■■■■■■■■■	EAI	EAI5	EAI
205	■■■□■■■■■■■■■■■■■■■■■■■■■■■■■■■■□□□□■■■■■■■	T	T1	T
206	■■■■□□□□□■■■■■■■■■■■□□□□■■■■■■■■□□□□■■■■■■■	LAM	LAM9	LAM
207	■■■■■□■■■■■■■■■■■■■■■■■■■■■■■■□■□□□□■■■■■■■	H	H3	H
208	■■■■■■■□□□□□■■■■■■■■■■■■■□□■□□□□■□■■■■■■■■■	EAI	EAI4	EAI
209	■■■■■■■■□□□□□□■■■■■■□□□□■■■■■■■■□□□□■■■■■■■	LAM	LAM12	LAM
210	■■■■■■■■□□□□□■■■■■■■■■■■■■■■■■■■□□□□■■■■■■■	T	T1	T
211	■■■■■■■■□□□■□■■■■■■■□□□□■■■■■■■■□□□□■■■■■■■	LAM	LAM3	LAM
212	■■■■■■■■□□■■■■■■■■■■■■■■■■□■■■■■□□□□■■■■■■■	T	S	T
213	■■■■■■■■□■■■■■■■■■■■□□□□■■■■■■■■□□□□■■■□■■■	LAM	LAM4	LAM
214	■■■■■■■■■■■■□□□□■■■■■■□■■■■■■■■■□□□□■■■■■■■	T	T5	T
215	■■■■■■■■■■■■□□□□■■■■■■■■■■■□■■■■□□□□■■■■■■■	T	T1	T
216	■■■■■■■■■■■■□□■■■■■■□□□□■■■■■■■■□□□□■■■■■■■	LAM	LAM9	LAM
217	■■■■■■■■■■■■□■■■■□■■■■■■■■■■■■■■□□□□■■■■■■■	X	X1	X
218	■■■■■■■■■■■■□■■■■■■■■■■■■□□□□□□■□□□□■■■■■■■	H	H1	T
219	■■■■■■■■■■■■■□□□□□■■■■■■■■■■■■■■□□□□■■■■■■■	T	T1	T
220	■■■■■■■■■■■■■□□□■□■■■■■■■■■■■■■■□□□□■■□□□□■	X	X2	X
221	■■■■■■■■■■■■■■□■■□■■■■■■■■■■■■■■□□□□■■■■■■■	X	X1	X

222	■■■■■■■■■■■■■■■■□□□□□■■■■■■■□■■■□□□□■■■■■■■	T	T1	T
223	■■■■■■■■■■■■■■■■■□□■■■■■■■■■■■■■□□□□■■□□□□■	T	T1	T
224	■■■■■■■■■■■■■■■■■□■■■■■■■■□□■■■■□□□□■■■□□■■	X	X1	X
225	■■■■■■■■■■■■■■■■■□■■■■■■■■■■■■■■□□□□■■□□□■■	X	X1	X
226	■■■■■■■■■■■■■■■■■□■■■■■■■■■■■■■■□□■■■■■■■■■	MANU	MANU2	X
227	■■■■■■■■■■■■■■■■■■□■■■■■□□□□□□□■□□□□■■■■■■■	H	H3	T
228	■■■■■■■■■■■■■■■■■■■■□□□□□■■■□■■■□□□□■■■■■■■	T	T1	T
229	■■■■■■■■■■■■■■■■■■■■□□□□□■■■■■■■□□□□□■■■■■■	U	U	Unassignable
230	■■■■■■■■■■■■■■■■■■■■□□□□□■■■■■■■□□□□■■■■■■■	T	T1	T
231	■■■■■■■■■■■■■■■■■■■■■■□■■■■■■■■■□□□□■■■□□□□	T	T5	T
232	■■■■■■■■■■■■■■■■■■■■■■■□□□□□□□□□□□□□□□■■■■■	U	U	Unassignable
233	■■■■■■■■■■■■■■■■■■■■■■■■□□□□□□■■□□□□■■■□■■■	T	T2	T
234	■■■■■■■■■■■■■■■■■■■■■■■■■■■■□□□□■□■■□■■■■■■	EAI	EAI5	EAI
235	■■■■■■■■■■■■■■■■■■■■■■■■■■■■□□□□■□■■■□■□■■■	EAI	EAI1	EAI
236	■■■■■■■■■■■■■■■■■■■■■■■■■■■■□□□□■□■■■■■■■■■	EAI	EAI5	EAI
237	■■■■■■■■■■■■■■■■■■■■■■■■■■■■■■□□□□□□□□□□□□□	U	U	H
238	■■■■■■■■■■■■■■■■■■■■■■■■■■■■■■□□□□□□□□■■■■■	U	U	Unassignable
239	■■■■■■■■■■■■■■■■■■■■■■■■■■■■■■■■□□□□□□□□■■■	U	U	Unassignable
240	■■■■■■■■■■■■■■■■■■■■■■■■■■■■■■■■□□□□□■■■■■■	U	U	Unassignable
241	■■■■■■■■■■■■■■■■■■■■■■■■■■■■■■■■□□□□■□□□□■■	T	T1	T
242	■■■■■■■■■■■■■■■■■■■■■■■■■■■■■■■■□□□□■□□□■■■	T	T1	T
243	■■■■■■■■■■■■■■■■■■■■■■■■■■■■■■■■□□□□■■□□□□□	T	T1	T
244	■■■■■■■■■■■■■■■■■■■■■■■■■■■■■■■■□□□□■■□□□□■	T	T1	T
245	■■■■■■■■■■■■■■■■■■■■■■■■■■■■■■■■□□□□■■□■■■■	T	T1	T
246	■■■■■■■■■■■■■■■■■■■■■■■■■■■■■■■■■■■■■□■■■	U	U	Unassignable
247	■■■□□□□■■■■■■■■■■■■■■■□□□□□□□□□□□□■■■□□■■■■	CAS	CAS1	CAS
248	■■■■■□□■■■■■■■■■■■■■■■■■■□□■□□□□■□■■■■■■■■■	EAI	EAI4	EAI
249	■■■■■■■■■■■□■□□□□□□□□□□□■■■■■■■■□□□□■■■■■■■	T	T1	T
250	□□□□□□□□□□□□□□□□□□□□□□□□□□□□□□□□□□□□□■■■■■■	BEIJ	BEIJ	Unassignable
251	■■■■■■■□□□□□□□□□□□□□□□□□□□□■■■■■□□□□■■■■■■■	T	T1	T
252	■■■■■■■■■■□□■■■■■■■■□□□□■■■■■■■■□□□□■■■■■■■	LAM	LAM9	LAM
253	■■■■■■■■■■■■■■■■■■■■□■■□□■■■■■■■□□□□■■■■■■■	T	T1	T
254	■■■■■■■■■■■■■■□□□□□□□□□□■■■■■■■■□□□□■■■■■■■	T	T5	T
255	□□□□□□□□□□□□□□□□□□□□□□□□□□□□□□□□□□■■■■□■■■■	BEIJ	BEIJ	BEIJ
256	■■■■■■■■■■■■■■■■■■■■■■■■■■■■□□□□■□■■■■□■■■■	EAI	EAI5	EAI
257	□□□■■■■■■■■■■■■■■■■■■■■■■■■■□□□□■□■■■■□□■■■	EAI	EAI5	EAI
258	□□□□□□□□□□□□□□□□□□□□■■■■■■■■■■■■□□□□■■■■■■■	T	T1	T
259	■■■■■■■□■■■■■■■■■■□■■■■■■■■■■■■■□□□□■■■■■■■	T	T1	T
260	□□□□□□□□□□□□□□□□□□□□□□□□□□□□□□□□□□■■□□■■■■■	BEIJ	BEIJ	BEIJ
261	■■■□■■■■■■■■■■■■■■■■■■■■□■■■■■■■□□□□■■■■■■■	T	T1	T
262	■■■■■■■□□■■■■■■■■■■■■■■■■■■□□□■□□□□■■■■■■■	H	H4	T
263	■■■■■■■■■■■□□□□□□□□□□□□□□■■■■■■■□□□□■■■■■■■	T	T1	T
264	■■■■■■■■■■■■■□□□□□□□□□□□□■■■■■■■□□□□■■■■■■■	T	T1	T
265	□□□□□□□□□□□□□□□□□□□□□□□□□□□□□□□□□□■■□■■■■■■	BEIJ	BEIJ	BEIJ
266	■■■■■■■□■■■■■□□□□□□□□□□□□■■■■■■■□□□□■■■■■■■	T	T1	T
267	■■■■■■■■■■■■□□□□□□□□□□□□□■■■■■■■□□□□■■■■■■■	T	T1	T
268	■■■■■■■■■■■■■■■■■■■■□■■■■■■■■■□■□□□□■■■■■■■	H	H3	H
269	□□□□□□□□□□□□□□□□□□□□□□□□□□□□□□□□□□□□■■■■■■■	BEIJ	BEIJ-LIKE	Unassignable
270	□■■□■■■■■■■■■■■■■■■■■■■■■■■■□□□□■□■■■■■□■■■	EAI	EAI1	EAI
271	■■■■■■■■□□□□□□□□□□□□□□□□□□□□□□□□□□□□□□■□□■■	U	U	Unassignable
272	□□□□■■■■■■■■■■■■■■■■■■■■■■■■■■■■□□□□■■■■■■■	T	T1	T
273	■■■■■■■■■■■□□□■■■■■■□□□□■■■■■■■■□□□□■■■■■■■	LAM	LAM9	LAM
274	■■■■■■■■■■■■■■■■■■■■□□□□□□□□□□□□□□□□□■■■■■■	U	U	Unassignable
275	■■■■■■■■■■■■■■■■■■■■□□□□□□□□□□□■□□□□■■■■■■■	H	H3	T
276	■■■■■■■■■■■■■■■■■■■■■■■■□□□□□□■■□□□□■■■■■■■	T	T1	T
277	■■■■■■■■■■■■■■■■■■■■■■■■■■■■□■■■□□□□■■■□□□□	T	T1	T

278	■■■■■■■■■■■■■■■■■■■■■■■■■■■■■■■■□□□□■■■■■□■	T	T1	T
279	■■□■■■■■■■■□■■■■■■■□□■■■■■■■□□□□■□■■■■■■■■■	EAI	EAI2	EAI
280	■■■■■■□□□□□□□□□□□□■■■■■■■■■■■■■■□□□□■■■■■■■	T	T1	T
281	■■■■■■■■■■■■■■■■□■■■■■■■■■■■■■■□□□□■■■■■■■	T	T1	T
282	■■■■■■■■■■■■■■■■■□■■■■□□■■■■■■■■□□□□■■□□□□■	X	X2	X
283	■■■■■■■■■■■■■■■■■■■■■□□□■□□□□□□■□□□□■■■■■■■	H	H1	T
284	□□□□■■■■■■■□□■■■■■■■■■■■■■■■■■■■□□□□■■■■■■■	T	T1	T
285	■■□□■■■■■■■■■■■■■■■■■■■■■■■■■■■■□□□□■■■■■■■	T	T1	T
286	■■□■■■■■■■■■■■■■■■■□□■■■□□□□□□□□□□□□□□□□□□□	U	U	animal
287	■■□■■■■■■■■■■■■■■■■□□■■■■■■■□□□□■□■■■■■■□■■	EAI	EAI2	EAI
288	■■■□□□□□□□■■■■■■■■■■■■□□□□□□□□□□□□■■■■■■■■■	CAS	CAS2	CAS
289	■■■□□□□■■■■■■■■■■■■■■■□□□□□□□□□□□□■■■□■■■■■	CAS	CAS1	CAS
290	■■■■■■■■■■■■■■■■■■■■□□□□■■□■■■■■□□□□■■■■■■■	LAM	LAM8	LAM
291	■■■■■■■■■■■■■■■■■■■■□■■■■■■■■■■■□□□□■■■■■■■	T	T1	T
292	■■■■■■■■■■■■■■■■■■■■■■□■■■■■□□□□■□■■□■■■■■■	EAI	EAI6	EAI
293	■■■■■■■■■■■■■■■■■■■■■■■■■■■■■■□■□□□□■■■■□■■	H	H3	H
294	■□■■■■■■■■■■■■■■■■■■■■■■■■■■■■□■□□□□■■■■■■■	H	H3	H
295	■■■■■■■■□□■■■■■■■■■■■■■□□■■■■■■■□□□□■■■■■■■	T	S	T
296	■□□■■■■■■■□■■■□■■■■■■■■■■■■■□□□□■□■■□□□■■■■	EAI	EAI3	EAI
297	■□□■■■■■■■■■■■□■■■□■■■■■■■■■□□□□■□■■■■□■■■■	EAI	EAI5	EAI
298	■□□■■■■■■■■■■■□■■■■■■■■■■■■■□□□□■□■■□□□■■■■	EAI	EAI3	EAI
299	■□□■■■■■■■■■■■■■■□□□□■■■■■■■□□□□■□□■□□■■■■■	U	U	EAI
300	■□■■■■■■■■■■□■□■■■■■□■■■■□□■□□□□■□■■■■□■■■■	EAI	EAI4	EAI
301	■□■■■■■■■■■■■■□■■■■■■■■■■■■■□□□□■□■■■■□□■■■	EAI	EAI5	EAI
302	■■■■■■■■■■■■■□■■■□■■■■■■■■■■■■■■□□□□■■■■■■■	X	X1	X
303	■■■■■■■■■■■■■■□■■■■■■■■■■■■■□□□□■□■■□■■□■■■	EAI	EAI1	EAI
304	■□■■□□□□□■■■■■■■■■■■■■□■■■■■■■■■□□□□■■■■■■■	T	T5	T
305	■■■■■■■■■■■■■■■■■■■□□■■■■■■■■■■■□□□□■■□□■■■	T	H37Rv	T
306	■■■■■■■■■■■■■■■■■■■■□□□□□□■■□■■■□□□□■■■■■■■	T	T1	T
307	□□□■■■■■■■■■■■■■■■■■■■■■■■■■■■□■□□□□■■■□■■■	H	H3	H
308	■■□■■■■□□□□□□□□□□■■■■■■■■■■■■□■■■■■■■■□□□□□	animal	BOV	animal
309	■■■□□□□■■■■■■■□■■■■■■■□□□□□□□□□□□□■■□□■■■■■	CAS	CAS1	CAS
310	■■■■■■■■■■■■□■□■■■■■■■■■□□□□□□□■□□□□■■■□■□□	H	H3	T
311	■■■■■■■■■■■■■■□■■■■□■■■■□□□□□□□■□□□□■■■□■■■	H	H3	T
312	■■■■■■■■■■■■■■□■■■■■■■■■□□□□□□□■□□□□■■■□■■■	H	H3	T
313	■■■■■■■■■■■■■■■■□□□□□□□□□■■■■■■■□□□□■■■□■■■	T	T2	T
314	■■■■■■■■■■■■■■■■■■■□■■■■□□□□□□□■□□□□■■■□■■■	H	H3	T
315	■■■■■■■■■■■■■■■■■■■■■■■□■□□□□□□■□□□□■■■□■■■	H	H1	T
316	■■■■■■■■■■■■■■■■■■■■■■■■□□□□□□□■□□□□■■■□■■■	H	H3	T
317	■■■■■■■■■■■■■■■■■■■■■■■■■■■□■■■■□□□□■■■□■■■	T	T2	T
318	■□■■■■□□□■■■■■■■■■■■■■■■■■■■■■■■■■■■□■■■■	AFRI	AFRI1	Unassignable
319	■□■■■■■□□□□□■■■■■■■■□□□□■■■■■■■■■■■■□□□■■■■	AFRI	AFRI2	AFRI
320	■■■■■■□□□□□□□□□□■■■■□□□□■■□□■■■■■■■■□□□■■■■	AFRI	AFRI	Unassignable
321	■■■■■■□□□■■■□■■■■■□□□□□□■□□□□■■■■■■■■■□■■■■	AFRI	AFRI1	Unassignable
322	■■■■■■□□□■■■■■□■■■■■■■■■■■■■■■□■■■■■■■□■■■■	AFRI	AFRI1	H
323	■■■■■■□□□■■■■■□■■■■■■■■■■■■■■■■■□■■■■■□■■■■	AFRI	AFRI1	Unassignable
324	■■■■■■□□□■■■■■□■■■■■■■■■■■■■■■■■■■■■■■□■■■■	AFRI	AFRI1	Unassignable
325	■■■■■■□□□■■■■■■■■■■■□■□□□■■■■■■■■■■■■■■□■■■■	AFRI	AFRI1	Unassignable
326	■■■■■■□□□■■■■■■■■■■■■■□□□■■■■■■■■■■■■■■□■■■■	AFRI	AFRI1	Unassignable
327	■■■■■■■□□□□□■■□■■■■■□□□□■■□■■■□■□■■■□□□■■■■	AFRI	AFRI2	AFRI
328	■■■■■■■□□□□□■■□■■■■■□□□□■■□■■■■■□■■■□□□■■■■	AFRI	AFRI2	AFRI
329	■■■■■■■□□□□□■■□■■■■■□□□□■■■■■■■■■■■■□□□■■■■	AFRI	AFRI2	AFRI
330	■■■■■■■□□□□□■■■■■■■■□□□□■■■■■■■■■■■■□□□□■■■	AFRI	AFRI	Unassignable
331	■■■■■■■□□□□□■■■■■■■■□□□□■■■■■■■■■■■■□□□■■■■	AFRI	AFRI2	AFRI
332	■■■■■■■■□■■■□□□■■■■■■■■■■■■■■■□■■■■■□□□■■■■	AFRI	AFRI	Unassignable
333	■■■■■■■■■■■■□■□■■■■■□□□□■■■■■■■■□□□□■■■■■■■	LAM	LAM5	LAM

334	■□■■■■■■■■■■■■■■■■■■■■■■■■■■■■■■□□□□■■■■■■■	T	T1	T
335	■■■■■■■■■■■■■■■■■■■■□□□□■■■■■■□■□□□□■■■■■■■	H	H3	Unassignable
336	■■■■■■■■■■■■■■■■■□■■■■■■■■■■■■■■□□□□■■■□■■■	X	X1	X
337	■■■■■■■■■■■■■■■■■■■■■■■■■■■□□□□□■□■■■■■□■■■	MANU	MANU1	MANU
338	□□□■■■■■■■■■■■■■■■■■■■■■■■■■□□□□■□■■□□□■■■■	EAI	EAI3	EAI
339	■□□□■■■■■■■■■■■■■□■■■■■■■■■■■■■■□□□□■■□□□□■	X	X2	X
340	■□□■■■■□□□■■■■■■■■■■■■■■■■■■□□□□■□■■■■■■■■■	EAI	EAI5	EAI
341	■□□■■■■■■■■■■■■■■■■■■■■■■□■■□□□□■□■■□□□□■■■	EAI	EAI5	EAI
342	■■□■■■■■■■■■■■■■■■■■■■■■■■■■□□□□■□■■■■■■■■■	EAI	EAI5	EAI
343	■■■□□□□□□□□□□□□□□□□□□□□□□■■■□□■■■■■□■■■■■■■■	U	U	Unassignable
344	■■■□□□□□□□□□■■■■■■■■■■■■■■■■■■■■□□□□■■■■■■■	T	T1	T
345	■■■■■■■■■□□□□□□□□□□■■■■■■■■■■■■■□□□□■■■□■■■	T	T3	T
346	■■■■■■■■■□■■■■■■■■■■■□□□□□□□□□□□□□■■■□■□■■■	U	U	Unassignable
347	■■■■■■■■■■■■□■■■■□■■■■■■■■■■■■■■□□□□■■□□□□■	X	X2	X
348	■■■■■■■■■■■■□■■■■□■■■■■■■■■■■■■■□□□□■■□■■■■	X	X1	X
349	■■■■■■■■■■■■□■■■■■■■■■■■■■■■■□□□□■□■■■■■□■■■	EAI	EAI1	EAI
350	■■■■■■■■■■■■■■■■■□■■□□□■■■■■■■■■□□□□■■□□□□■	X	X2	X
351	■■■■■■■■■■■■■■■■■□■■■■■■■■■■■□□□□□□□□□■■■■■	U	U	X
352	■■■■■■■■■■■■■■■■■□■■■■■■■■■■■■■■□□□□■■■□□■■	X	X1	X
353	■■■■■■■■■■■■■■■■■■■■■■■■■□□■■■■■□□□□■■■■■■■	T	T1	T
354	■■■■■■■■■■■■■■■■■■■■■■■■■■■■□□□□□□■■■■■□■■■	U	U	Unassignable
355	■□□■■■■■■■■■■■■■■■■■■■■■■■■■□□□□■□■■□□□□■■■	EAI	EAI5	EAI
356	■■■□□□□■■■■■■■■■■■■■□□□□□□□□□□□□□□□■■■■■■■■	CAS	CAS	CAS
357	■■■□□□□■■■■■■■■■■■■■■■□□□□□□□□□□□□□□■■■■■■■	CAS	CAS	CAS
358	■■■□□■■■■■■■■■■■■■■■■■■■■■■■■■■■□□□□■■■■■■■	T	T1	T
359	□□□□■■■■■■■□■■■■■■■■■■■■■□□□□□□■□□□□■■■■■■■	H	H1	T
360	■□■■□□□□□□■■■■■■■■■■■■■■■■■■■■□■□□□□■■■■■■■	H	H3	H
361	■□■■■■■■■■■■□■■■■■■■■■■■■■■■■□□□■□□□□■■■■■■■	H	H4	T
362	■■□■■■■□■■■■■■■■■■■■■■■□■■■■■■■■■□□□□■■■■■■■	T	T5	T
363	■■■□□■■■□□■■■■■■■■■■■■■■■■■■■■■■□□□□■■■■■■■	T	S	T
364	■■■■□■■■■■■■■■■■■■■■■■■■■□■■■■■□□□□■■■■■■■	T	T1	T
365	■■■■■■■■■□□□□□□□□□□□□□□□□■■■■■■■□□□□■■■■■■■	T	T1	T
366	■■■■■■■■■■□■■■■■■■■■■■■■■■■■■■■■□□□□■□□■■■■	T	T1	T
367	■■■■■■■■■■■■□■■■■■■□□□□□■□□■■■■■□□□□■■■■■■■	LAM	LAM7	T
368	■■■■■■■■■■■■■■■■■■□□□□□□■■■■■■□■□□□□■■■□■■■	H	H3	H
369	■■■■■■■■■■■■■■■■■■■□■■■■■□□□□□□■□□□□■■■■■■■	H	H1	T
370	■■■■■■■■■■■■■■■■■■■■■■□□■■■■■■■■□□□□■□□■■■■	T	T1	T
371	■■■■■■■■■■■■■■■■■■□□□□□□■■■■■■□■□□□□■■■■■■■	H	H3	H
372	■■■■■■■■■■■■■■■■■■■■■■□■□■□□□□□□□□□□□□□□□	U	U	Unassignable
373	■■■■■■■■■■■■■■■■■■■■■■■□■■■■■■■■□□□□■■■■■■■	T	T1	T
374	■■■■■■■■■■■■■■■■■■■■■■■■■■□□□□□□□□□□■■■■■■■	U	U	Unassignable
375	□□□■■■■■■■■■□■■■■■■■□□□□■■■■■■■■□□□□■■■■■■■	LAM	LAM5	LAM
376	□■■■■■■■□□□■■■■■■■■■□□□□■■■■■■■■□□□□■■■■■■■	LAM	LAM3	LAM
377	■■□■□■■■■■■■□■■■■■■■□□□□■■■■■■■■□□□□■■■■■■■	LAM	LAM2	LAM
378	■■■■■■■■■■■■■■■■■■■■□■■□■■■■■■■■□□□□■■■■■■■	T	T1	T
379	■■■■■■■■■■■■■□□□□■□□□□□■■■■■■■■■□□□□■■■■■■■	T	T1	T
380	■□■■■■■■■■■■■■■■■■■■■■■■■■■■□□□□■□■■■■■■■■■	EAI	EAI5	EAI
381	■■■□□□□■■■■■■■■■■■■■■■□□□□□□□□□□□□■■□□□■■■■	CAS	CAS1	CAS
382	■■■■□■■■■■■■■■■■■■■■■■■■■□□□□□□■□□□□■■■■■■■	H	H1	T
383	■■■■■■■■□□■■■■■■■■■■■■■■■□□□□□□■□□□□■■■■■■■	H	H1	T
384	■■■■■■■■■■■■■■■■■■■■□■■■■□□□□□□■□□□□■■■■■■■	H	H1	T
385	■■■■■■■■■■■■□■■■■□□■■■■■■■■■■■■■□□□□■□■■■■■	T	T3	T
386	□□□□□□□□□□□□□□□□□□□□□□□□■■■□■■■■□□□□■■■■■■■	T	T1	T
387	■□□□□□□□□□□□□□■■■■■■□□□□□□□■□■■■□□□□■■■□■■■	T	T2	T
388	■■■□■■■■■■■■■■■■■■■■□□□□■■■■■■■■□□□□■■■■■■■	LAM	LAM9	LAM
389	■■□■■■■■■■■■■■□■■■■■□□□□■■■■■■■■□□□□■■■■■■■	LAM	LAM1	LAM

390	■■■■■■■■■■■■■■■■■■■■■■■■■■■■■□□■□□□□■■■■■■■	H	H3	T
391	■■■■□□□■■■■■■■■■■■■■■□□□□■■■■■■■■□□□□■■■□■■■	LAM	LAM4	LAM
392	■■■■■■■■■■■■■■■■■■■■□□□□□□□□□■■■□□□□■■■□■■■	T	T2	T
393	■■■■■■■■■■■■■□■■■■■■■■■■■■■■■■■■■■□□□□■■■■■■■	T	T1	T
394	■■■■■■■■■■■■■■■■■■■■■■■■■■■■■□□■□□□□■■■□■■■	H	H3	T
395	■■■■■■■■■■■■■■■■■■■■■■■■■■■■■■■■□□□□■□□□□□□	T	T1	T
396	■■■■■■■■■■■■■■■■■■■■□□□□■■■■□■■■□□□□□■■■■■■	U	U	LAM
397	■■■■■■■■■■■■■■■■■■■■□□□□□□□□□□□□□□□□■■■■■■■	U	U	Unassignable
398	■■■■■■■■■■■■■■■■■■■■□□□□■■■■■■■■□□□□■■□□■■■	LAM	LAM9	LAM
399	■■■■■■■■■■■■■■■■■■■■■■■■■■■■□□□■□□□□■□□□□■□	H	H4	T
400	■■■■□■■■■■■■■■■■■■■■■■■■■□□□□□□■□□□□■■■□■■■	H	H1	T
401	■■■■■■■■□□■■■■■■■■■□□□□□□■■■■■■■□□□□■■■■■■■	T	S	T
402	■■■■■■■■■■■■■■■■■■■■□□□□□□□□□□□□□□□□□□□□□□□	U	U	LAM
403	■■■■■■■■■■■■■■■■■■■■■■□□□■■■■■■■□□□□■■■□■■■	LAM	LAM10	T
404	■■■■■■■■■■■□■□□■■■□□□□□□□□□□□□□□□□□□□□□□□■■	U	U	Unassignable
405	■■■■■■■■■■■■■■■■■■□□□□□□□□□□□□□□□□□□□□□□□■■	U	U	Unassignable
406	□□□□□□□□□□□□□□□□□□□□□□□□□□□□□□□□□□□□■■■□■■■	BEIJ	BEIJ-LIKE	Unassignable
407	□□□□□□□□□□□□□■■■■□□□□■□□□□□□□□□□□□■■■■■■■■■	CAS	CAS	CAS
408	□□■■□■■■■■■■■□■■■□■■■■■■■■■■■■■■□□□□■■■■■■■	X	X1	X
409	□■□■■□■□□□□□■■■□■■■■■■■■■■■■■■■■■■■■■■□□□□□	animal	BOV2	animal
410	□■■■■■■■■■■■■■■■□□□□□■■■■■■■■■■■□□□□■■■■■■■	T	T1	T
411	■□□□□□■■■■■■■■■■■■■■■■■■■■■■■■■■□□□□■■■■■■■	T	T1	T
412	■□□□■■■■■■■■■■■■■■■■□□□□■■□□□□■■□□□□■■□□□□□	LAM	LAM11	LAM
413	■□□■■■■■■■■■■■■■■■■□□■■■■■■■□□□□■□■■■■■■■■■	EAI	EAI5	EAI
414	■□□■■■■■■■■■■■■■■■■■■■■□■■■■□□□□■□■■□□□■■■■	EAI	EAI3	EAI
415	■□□■■■■■■■■■■■■■■■■■■■■■■□□□□■□■■■■■■■■■■■■	U	U	H
416	■■□□□■■■□■■■■■■■■■■■□■■■■■■■■■■■■■■■■■□□□□□	animal	BOV2	animal
417	■□■■■■■■■■■■■■■■■■■■■■■■□□□□□□■■□□□□■■■■■■■	T	T1	T
418	■■□□□■■■■■■■■■■■■■■■■■■■■■■■■□■□□□□■■■■■■■	H	H3	H
419	■■□□■■□□□□□□□□□□□□□■□□□□■■■■■■■■□□□□■■■■■■■	LAM	LAM9	LAM
420	■■□□■■■■■■■■■■■■□□■■■■■■■■■■■■■■□□□□■■■□■■□	T	T2	T
421	■■□■■■■■□■■□□□□□□□□□□□□□□□□□□□□□□□□□■■■■■■	U	U	animal
422	■■□■■■■■□■■■■■■□■■■■□■■□■■■■■■■■■■■■■■□□□□□	animal	BOV1	animal
423	■■□■■■■■■■■■■■■■□□□■□□□□■■■■■■■■□□□□■■■■■■■	LAM	LAM1	LAM
424	■■□■■■■■■■■■■■■■■■■□□■■■■■■■□□□□■□■■■■■□□■■	EAI	EAI2	EAI
425	■■■□□□□□□□□□■■■■■□■■■■■■■□□□□■■■□□□□■■■■■■■	X	X3	X
426	■■■□□□□□□□■■■■■■■■■■□□□□□□□□□□□□□□■■■■■■■■■	CAS	CAS	Unassignable
427	■■■□□□□■■■■■□□□■■■■■■■□□□□□□□□□□□□■■■■■■■■■	CAS	CAS1	CAS
428	■■■□□□□■■■■■■■■■■■■■■■□□□□□□□□□□□□■■□■■■■■■	CAS	CAS1	CAS
429	■■■□□□□■■■■■■■■■■■■■■■□□□□□□□□□□□□■■■■■□■■■	CAS	CAS1	CAS
430	■■■□□□■□□□□□□□□□■■□■■■□□■■■■■■■■□□□□■□□■■■■	T	T4	T
431	■■■□□■■■■■■■■■■■■■■■■■■■■□□□□□□■□□□□■■■■■■■	H	H1	T
432	■■■□■■■□■■■■■■■■■■■■■■■■■■■■■■■■□□□□■■■□■■■	T	T2	T
433	■■■■□■■■■■■■■■■■■■■■■■■■■■■□■■□■□□□□■■■■■■■	H	H3	H
434	■■■■■□□□□■■■■■■■■■■■■■■■■■■■■■■■□□□□□□■■■■■■	U	U	Unassignable
435	■■■■■□□■■■■■■■■■■■■■□□□□■■■■■■■■□□□□■■■■■■■	LAM	LAM9	LAM
436	■■■■■■□□□□■■■■■■■□■■■■■■■■■■■■■■□□□□■■□□□□■	X	X2	X
437	■■■■■■■□□□□□■■■■■■■■■■■■■■■■■■■■■■□■□□□■■■■	AFRI	AFRI3	AFRI
438	■■■■■■■□□□□□■■■■■■■■■■■■■■■■■■■■■■■■□□□■■■■	AFRI	AFRI3	AFRI
439	■■■■■■■■■□■■■■■■■□■■■■■■■■■■■■■■□□□□■■□□□□■	X	X2	X
440	■■■■■■■■■■■□□□□■■■■■□□□□■■■■■■■■□□□□■■■■■■■	LAM	LAM9	LAM
441	■■■■■■■■■■■■□□□□□□□■□■■■■■■■■■■■□□□□■■■■■■■	T	T1	T
442	■■■■■■■■■■■■□■■■■■□■■■■■■■■■■■■■□□□□■■■■■■■	T	T3	T
443	■■■■■■■■■■■■□■■■■■■■□□□□■■■■■■□□□□□□■■■■■■■	U	U	LAM
444	■■■■■■■■■■■■■□□□□□□□□□□□□□■■■■■■□□□□■■■■■■■	T	T1	T
445	■■■■■■■■■■■■■□■■■□■■■■■■■■■■■■■■□□□□■■□□□□■	X	X2	X

446	■■■■■■■■■■■■■■□□□□□□□□□■■■□■■■■■□□□□■■□□□□■	T	T1	T
447	■■■■■■■■■■■■■■□□□□□■■■■■■■■■■■■■■□□□□■■■■■■■	T	T1	T
448	■■■■■■■■■■■■■■□■■■■■■■■■■■■■■■■□■□□□□■■■□■■■	H	H3	H
449	■■■■■■■■■■■■■■■■■□■■■■□■■■■■■■■■■□□□□■■■■■■■	X	X1	X
450	■■■■■■■■■■■■■■■■■□■■■■■■□□□□□□□□□□□□□□□□□□□	U	U	X
451	■■■■■■■■■■■■■■■■■■■□□■■■■■■■■■■■□□□□■■■■■■■	T	H37Rv	T
452	■■■■■■■■■■■■■■■■■■■■□□□□■■□□■■■■□□□□■■■■■■■	LAM	LAM9	LAM
453	■■■■■■■■■■■■■■■■■■■■□■■■■■■■□■■■□□□□■■■■■■■	T	T1	T
454	■■■■■■■■■■■■■■■■■■■■■□■■■■□■■■■■□□□□■■■□■■■	T	T2	T
455	■■■■■■■■■■■■■■■■■■■■■■■□□□□□■■■■□□□□■■■■■■■	T	T1	T
456	■■■■■■■■■■■■■■■■■■■■■■■■■□□■□□□□■□■■■■■□□■■	EAI	EAI4	EAI
457	■■■■■■■■■■■■■■■■■■■■■■■■■■■□■■□■□□□□■■■■■■■	H	H3	H
458	■■■■■■■■■■■■■■■■■■■■■■■■■■■■□□□□□□■■■■■■■■■	U	U	Unassignable
459	■■■■■■■■■■■■■■■■■■■■■■■■■■■■□□□□■□□□□■■□■■■	U	U	EAI
460	■■■■■■■■■■■■■■■■■■■■■■■■■■■■□□□□■□■■■■■■□■■	EAI	EAI5	EAI
461	■■■■■■■■■■■■■■■■■■■■■■■■■■■■□□□□■■■□■■■■■□■	U	U	EAI
462	■■■■■■■■■■■■■■■■■■■■■■■■■■■■□■■■□□□□■■■■■■■	T	T1	T
463	■■■■■■■■■■■■■■■■■■■■■■■■■■■■■■□■□□□□■□■■■■■	H	H3	H
464	■■■■■■■■■■■■■■■■■■■■■■■■■■■■■■■□□■■■■■■■■■■	U	U	Unassignable
465	■■■■■■■■■■□□□□■■■■■■■□■■■■■■■■□□□□□□□□□□□□□	U	U	Unassignable
466	■■■■■■■■□□■■■■■■■■□■■■■■■■■■■■■■■□□□□■■■■■■■	T	S	T
467	□□□□□□□□□□□□□□□□□□□□□□□□□□□□□□□■□□□□■■■■■■■	H	H3	T
468	□□□■■□■■■■■■■■■■■■■■■■■■■■■■□□□□■□■■□□□□■■■	EAI	EAI5	EAI
469	□□□■■■■■■■■■■■■■■■■■□□□□■■■■■■■■□□□□■■■■■■■	LAM	LAM9	LAM
470	□□■■■■■■■■■■■■■■■■■■■■■■■■■■□□□□■□■■■■■■■■■	EAI	EAI5	EAI
471	□■■□□□□■■■■■■■■■■■■■■■□□□□□□□□□□□□■■■■■■■■■	CAS	CAS1	CAS
472	□■■■■■■■■■■■■■■■■■■■■■■■■■■■■■□■□□□□■■■■■■■	H	H3	H
473	■□□□□□□□■■■■■■■■■■■■■■■■■■■■□□□□■□■■□□□■■■■	EAI	EAI3	EAI
474	■□□■■□■■■■■■■■■■■■■■■■■■■■■■□□□□■□■■□□□□■■■	EAI	EAI5	EAI
475	■□□■■■■■■■■■■■■■■□□□□■■■■■■■□□□□■□□■■■■■■■■	U	U	EAI
476	■□■■■■■■■■■■■■■■■□■■■■■■■■■■■■■■□□□□■■□□□□■	X	X2	X
477	■□■■■■■■■■■■■■■■■■■■■■■■■■■■□□□□■□■■■■■□■■■	EAI	EAI1	EAI
478	■■□□□■■■■■■■■■■■■□■■■■■■■■■■■■■■□□□□■■□□□□■	X	X2	X
479	■■□■□□□□□□□□□□■□■■■■■■■■■■■■■■■■■■■■■■□□□□□	animal	BOV3	animal
480	■■□■■■■□□■■■■■■■■■■□□■■■■■■■□□□□■□□■■■■■■■■	U	U	EAI
481	■■□■■■■■□■■■■■■□■■■■□■■■■■■■■■■■■■■■■■■□□□□□	animal	BOV1	animal
482	■■□■■■■■□■■■■■■□■■■■■■■■■■■■■■■■■■■■■■□□□□□	animal	BOV1	animal
483	■■□■■■■■■■■■■■■■■■■□□■■■■■■■□□□□■□■■■■■□□□■	EAI	EAI2	EAI
484	■■■□□□□□□□□□□■■■■□■■■■■■■■■■■■■□□□□□□□■■■■■	U	U	X
485	■■■□□□□■■■■■■■■■■■■□□□□□□□□□□□□□□□■■■■■■■■■	CAS	CAS	CAS
486	■■■□□□□■■■■■■■■■■■■■■■■□□□□□□□□□□□□□□□■■■■■■	CAS	CAS	CAS
487	■■■■□□□□□□□□□□□□□□□□□□□□■□□□□□□■□□□□■■■■■■■	H	H3	T
488	■■■■■□□□□■■■■■■■■■■■■■■■■□□□□□□■□□□□■■■■■■■	H	H1	T
489	■■■■■□■■■■■■■■■■■□■■■■■■■■■■■■■■□□□□■■□□□□■	X	X2	X
490	■■■■■□■■■■■■■■■■■□■■■■■■■■■■■■■■□□□□■■■■■■■	X	X1	X
491	■■■■■■□■■■■■■■■■■□■■■■■■■■■■■■■■□□□□■■□□□□■	X	X2	X
492	■■■■■■□■■■■■■■■■■■■■□□□□■■■■■■■■□□□□■■■■■■■	LAM	LAM9	LAM
493	■■■■■■■□□□□■■■■■■■■■■■■■■■■■□□□□■□■■■■■□■■■	EAI	EAI1	EAI
494	■■■■■■■■□□■■■■■■■■■■■■■■■■■■■■■■□□□□■■■□□■■	T	S	T
495	■■■■■■■■□■■■■■□□□□□□□□□□□■■■■■■■□□□□■■■■■■■	T	T1	T
496	■■■■■■■■■■□□■■□□□□□□□□□□■■■■■■■■□□□□■■■■■■■	T	T5	T
497	■■■■■■■■■■■□■■■■■■■■■■■■■■■■□□□□■□■■■■■■■■■	EAI	EAI5	EAI
498	■■■■■■■■■■■□■■■■■■■■■■■■■■■■■■■■□□□□■■■■■■■	T	T1	T
499	■■■■■■■■■■■■□□□□□□□□□□□□□□□□□□■■□□□□■■■■■■■	T	T1	T
500	■■■■■■■■■■■■□□□□□□□□□□□□□□■■■■■■□□□□■■■■■■■	T	T1	T
501	■■■■■■■■■■■■□□□□□□□■■■■■■■■■■■■■□□□□■■■■■■■	T	T1	T

502	■■■■■■■■■■■■□■□■■■■■■□■■■■■■■■■■□□□□■■■■■■■	T	T3	T
503	■■■■■■■■■■■■□■■■■■□■■■■■■■■■■■■■■□□□□■■■□■■■	T	T2	T
504	■■■■■■■■■■■■□■■■■■■■■□■■■■■■■■■■□□□□■■■■■■■	T	T3	T
505	■■■■■■■■■■■■□■■■■■■■■■■■■■■■■■■■□□□□■■■□□□□	T	T3	T
506	■■■■■■■■■■■■■■□□□□□□□□□□□■■■■■■■□□□□■■■■■■■	T	T1	T
507	■■■■■■■■■■■■■■■■■□□□□□■■■■■■■■■■□□□□■■■■■■■	T	T1	T
508	■■■■■■■■■■■■■■■■■□□■■■■■■■■■■■■■□□□□■■■■■■■	T	T1	T
509	■■■■■■■■■■■■■■■■■■■■□□□□■□■■■■■■□□□□■■■■■■■	LAM	LAM9	LAM
510	■■■■■■■■■■■■■■■■■■■■■□□□□□□□□□□□□□■■■□■□■■■	U	U	Unassignable
511	■■■■■■■■■■■■■■■■■■■■■□□□□□□□□□□■□□□□■■■■■■■	H	H3	T
512	■■■■■■■■■■■■■■■■■■■■■□□□■■■■■■□■□□□□■■■■■■■	H	H3	H
513	■■■■■■■■■■■■■■■■■■■■■□□■■■■■■■■■□□□□■■■□■■■	T	T2	T
514	■■■■■■■■■■■■■■■■■■■■■■■■■□□■□□□□■□■■■■■□■■■	EAI	EAI4	EAI
515	■■■■■■■■■■■■■■■■■■■■■■■■■■□■■■■■□□□□■■■□■■■	T	T2	T
516	■■■■■■■■■■■■■■■■■■■■■■■■■■■□■■■■□□□□■■■■■■■	T	T1	T
517	■■■■■■■■■■■■■■■■■■■■■■■■■■■■□□□□■□■■■■■□□■■	EAI	EAI5	EAI
518	■■■■■■■■■■■■■■■■■■■■■■■■■■■■■□■■□□□□□□□□■■■	U	U	Unassignable
519	■■■■■■■■■■■■■■■■■■■■■■■■■■■■■■■□□□□□□■■■■■■	U	U	Unassignable
520	■■■■■■■■■■■■■■■■■■■■■■■■■■■■■■■■□□□□■□■■■■■	T	T1	T
521	■■■■■■■■■■■■■■■■■■■■■■■■■■■■■■■■□□□□■■□□□■■	T	T1	T
522	■■■■■■■■■■■■■■■■■■■■■■■■■■■■■■■■□□□□■■■■■■□	T	T1	T
523	■■■	U	U	Unassignable
524	■■■■■■■■■■■■■■■■■■■■■■■■■■■■■■□■□□□□■■■□□■■	H	H3	H
525	■□■■■■□□□■■■■■■■■■■■□□■■■■■■■■■□□□■■■■□■■■■	AFRI	AFRI1	Unassignable
526	■■□■■■■■■■■■■■■■■■■■■■■■■■■■□□□□□□■■■■□■■■■	U	U	animal
527	■■□■■■■■■■■■■■■■■■■■■■■■■■■■□□□□□□■■■■■■■■■	U	U	animal
528	■■□■■■■■■■■■■■■■■■■■■■■■■■■■□□□□■□■■■■□■■■■	EAI	EAI5	EAI
529	■■□■■■■■■■■■■■■■■■■■■■■■■■■■□□□□■□■■■■■□■■■	EAI	EAI1	EAI
530	■■■■■■□□□■■■■■■■■■■□□■■■■■■■■■■■■■□■■■□■■■■	AFRI	AFRI1	Unassignable
531	■■■□■■■■■■■■■■■■■■■■■■■■■□□□□□□■□□□□■■■■■■■	H	H1	T
532	■■■■■■□□□■■■■■■■■■■■■■■■□■■■■■■■■■■■■■■□■■■■	AFRI	AFRI1	Unassignable
533	■■■■■■■■■■□□□□□□□□■■■■■■■■■■■■□■□□□□■■■■■■■	H	H3	H
534	■■■■■■■■■■■■■■■■■■■■□□□□■■■■□□□□□□□□□□□□□□□	U	U	LAM
535	■■■■■■■■■■■■■■■■■■■■■□□□■■■■■■■■□□□□■■■■■■■	T	T1	T
536	■■■□□□□□□□□□□□□□□□□■■■■■■■■■■■■■■■■□■■□■■■■	AFRI	AFRI	Unassignable
537	■■■■■■□□□■■■■■■■■■■■■■■■■■■■■■■■■■■■□□□□■	AFRI	AFRI	Unassignable
538	■■■■■■■■■■■□□■■■■■■■■■■■■■■■□□□□■□■■■■■□■■■	EAI	EAI1	EAI
539	□□□□□□□□□□□□□□□□□□□□□□□□□□□□□□□□□□□□■■□□□□□	animal	MICROTI	Unassignable
540	□□□□□□□□□□□□□□□□□□□□□□□□□□□□□□□□□□■■□□□□■■■	BEIJ	BEIJ	BEIJ
541	□□□□□□□□□□□□□□□□□□□□□□□□□□□□□□□□□□■■■■■□□■■	BEIJ	BEIJ	BEIJ
542	□□□■■■■■■■■■■□■■■■■■□□□□■■■■■■■■□□□□■■■□■■■	LAM	LAM4	LAM
543	■□■■■■■■■■■■■■■■■■■■□■■■■■■■□■■■□□□■■■■■■■■	U	U	Unassignable
544	■■□□□□□□□□□□□□□□□□□□□□□□□□□■■■■■□□□□■■■□■■■	T	T2	T
545	■■□■■■■□■■■■□■■■■■■■□□□□■■■■■■■■□□□□■■■■■■■	LAM	LAM2	LAM
546	■■■□□□□□□□□□□■■■■□■■■■■■■■■■□■■■□□□□■■■■■■■	X	X3	X
547	■■■□□□□□□□□□□■■■■□■■■■■■■■■■■■■■□□□□□□□□■■■	U	U	X
548	■■■□□□□□□□□□■■■■■□■■■□□□□□□□□□□□□□□□□□□■■■■	U	U	X
549	■■■□□□□□□□□□■■■■■□■■■■□■■■■■■■■■□□□□■■■■■■■	X	X3	X
550	■■■□□■■■■■■■■■■■■■■□□■■■■■■■□□□□■□■■■■■□■■■	EAI	EAI1	EAI
551	■■■□■■■■■■■■■■■■■■■■■■■■■■■■■■■■□□□□■■□□□■□	T	T1	T
552	■■■■□□□□□□□□■■■■■□■■■■■■■■■■■■■■□□□□■■□□□□■	X	X2	X
553	■■■■□□□□■■■■□■■■■■■■■■■■■■■■■■■■□□□□■■■□■■■	T	T2	T
554	■■■■□■■■■■■■□□□□□□■■■■■■■■■■■■■■□□□□■■■■■■■	T	T1	T
555	■■■■□■■■■■■■■■■■■□■■■■■■■■■■■■■■□□□□■□■■■■■	X	X1	X
556	■■■■■■□■■■■□□□□□■■■■■■■■■□□□■■■■□□□□■■■■■■■	T	T1	T
557	■■■■■■□■■■■■■■■■■■■■■■■■■□□□□□□■□□□□■■■■■■■	H	H1	T

558	■■■■■■■■□□■■■■■■■■■■■■■■□□□■■■■■□□□□■■■□■■■	T	S	T
559	■■■■■■■■□□■■■■■■■■■■■■■■□□□■■■■■□□□□■■■■■■■	T	S	T
560	■■■■■■■■■□□□□□□□□□□□□□□□□□□□□□□□□□□□□■■■■■■	U	U	Unassignable
561	■■■■■■■■■■□■□□□□□□□□□□□□□■■■■■■■□□□□■■■■■■■	T	T1	T
562	■■■■■■■■■■■■□■■■■■■■■□□□□□□□□□□□□□□□□□□□□□□	U	U	Unassignable
563	■■■■■■■■■■■■□■■■■■■■■■■■□□□□□□□□□□□□□□□■■■■	U	U	Unassignable
564	■■■■■■■■■■■■□■■■■■■■■■■■■□□■□□□□■□■■■■■■■■■	EAI	EAI4	EAI
565	■■■■■■■■■■■■□■■■■■■■■■■■■■■■■■■■□□□□■■□■■■■	T	T3	T
566	■■■■■■■■■■■■■□□□□□■■■■■■■■■■■■■■□□□□■■■□■■■	T	T2	T
567	■■■■■■■■■■■■■■□□□□□□□□□□■■■■■■■■□□■■■■■■■■■	MANU	MANU2	Unassignable
568	■■■■■■■■■■■■■■□■■■■□□■■■■■■■■■■■□□□□■■■■■■■	T	H37Rv	T
569	■■■■■■■■■■■■■■□■■■■■■■■□□□□□□□□□□□□□□□□□□□□	U	U	Unassignable
570	■■■■■■■■■■■■■■■□■■■■■■■■■■□■■■■■□□□□■■■■■■■	T	T1	T
571	■■■■■■■■■■■■■■■■□■■■■■■■□□□□□□■□□□□■■■■■■■	H	H1	T
572	■■■■■■■■■■■■■■■■■□■□□□□□■■■□■■■■□□□□■■□□□□■	X	X2	X
573	■■■■■■■■■■■■■■■■■■■■□■■□□■■■□■■■□□□□■■■■■■■	T	T1	T
574	■■■■■■■■■■■■■■■■■■■■■■■■■■■■■■■□□□□□□□□■■■■	U	U	Unassignable
575	■■■■■■■■■■■■■■■■■■■■■■■■■■■■■■■■□□□□■□■■■□■	T	T1	T
576	■■■■■■■■■■■■■■■■■□■■■■■■■■■□■■■■□□□□■■■■■■■	X	X1	X
577	■□□■■■■■■■■■■■□■■■□■□■■■■■■■□□□□■□■■■■■■■■■	EAI	EAI5	EAI
578	■■□□■■■■■■■■■■■■■■■■□□□□■■■■■■■■□□□□■■■■■■■	LAM	LAM9	LAM
579	■■■■■■■■■■■■□■■■■■■■□□□□■■■■■■□□□□□□□□■■■■■	U	U	LAM
580	■■□■■■■■□□□□■■■■■■■■□□□□■■■■■■■■□□□□■■■■■■■	LAM	LAM1	LAM
581	■■■■■■■■■■■■■■■■■■■■□■■■■■■■□■■■□□□□■■□■■■■	T	T1	T
582	■■■■■■■■■□□□□□□□■□■■■■■■■■■■■■■■□□□□■■■□■■■	X	X1	X
583	■■■■■■■■■■■■□■■■■■■■■■■■■■■■■■■■□□■■■■■■■■■	MANU	MANU2	Unassignable
584	■■■■■■■■■■■■■■■■□■■■■■■■■■■■■■■■□□□□■■■□■■■	T	T2	T
585	□□■■■	BEIJ	BEIJ-LIKE	Unassignable
586	□□□□□□□□□□□□□□□□□□□□□□□□■■■■■■□■□□□□■■□□■■■	H	H3	H
587	■■■□□□□□□■■■■□□□□□□□□□□□■■■■■■■■■■□□■■□■■■■	AFRI	AFRI	Unassignable
588	■■■□□□□■■■■■■■■■■■■■■■□□■□□□□□□□□□■■■■■■■■■	U	U	CAS
589	■■■■■■■■□□□■■■□□□■□□□□□□□□□□□□□□□□□□□□□■■■■	U	U	Unassignable
590	■■■■■■■■■■■■■■■■■■■□□□□□□■■■■■■■□□□□■■■□■■□	T	T2	T
591	■■■■■■■■■■■■■■■■■■■■■■□■■■■■□□□□■□■■■■■■■■■	EAI	EAI6	EAI
593	□□□■■■■□□□□□□□□□□□□□□□■■■■■■■■■■■■■■■■□□□□□	animal	PIN	animal
594	■■□■□■■■□■□■■■■□■■■■■■■■■■■■■■■■■■■■■■□□□□□	animal	BOV1	animal
595	■■□■■■■■□■■■■■■■■■■■■■■■■■■■■■■■■■■■■■□□□□□	animal	BOV1	animal
596	■□□■□■■■■■■■■■■■■■■■■■■■■■■■□□□■□□□□■■■■■■■	H	H4	T
597	■□■■■□■■■■■■■■■■■■■■■■■■■■■■□□□■□□□□■■■■■■■	H	H4	T
598	■■■□□□□■■■■■■■■■■■■□□□□□□□□□□□□□□□□□□■■■■■■	CAS	CAS	CAS
599	■■■□□□□■■■■■■■■■■■■□□□□□□□□□□□□□□□□□■■■■■■■	CAS	CAS	CAS
600	■■■□□□□■■■■■■■■■■■■□□□□□□□□□□□□□□□■■□□■■■■■	CAS	CAS	CAS
601	■■■□□□□□□□■■■■■■■■■□□□□□□□□□□□□□□□□□■■■■■■■	CAS	CAS	Unassignable
602	■■■■■■■■■■■■■■■■■■■■■■■■□□□□□□□□□□□□■■■■■■■	U	U	Unassignable
603	■■■■■■■■■■■■■■■■■■■■■■■■■■■□■■□■□□□□■■■□■■■	H	H3	H
604	■■□■■■■■■■■■□■■■■■■■□□□□■■■■■■■■□□□□■■■■□■■	LAM	LAM2	LAM
605	■■■■■■■■■■■■□■■■■■■■□□□□■■■■■■□□□□□□□□□□■■■	U	U	LAM
606	■■■■■■■■■■■■□■■■■■■■□□□□■■■■■■■■□□□□□□□□□□□	U	U	LAM
607	■■■■■■■■■■■■■■■■■■■□□■■□■■■■□□□□□□□□□□□□□□□	U	U	Unassignable
608	■■■■■■■■□□□■■■■■■■■■■■■■□□□□□□□□□□□□□□□□□□□	U	U	Unassignable
609	■■■■■■■■■■■□□□□□□□■■■■■■■□□□□□□■□□□□■■■■■■■	H	H1	T
610	■■■■■■■■■■■■■■□■■■■■■■■■■□□□□□□■□□□□■■■□■■■	H	H1	T
611	■■■■■■■■■■■■■■□■■■■■■■■■■■■■■□■■■□□□□■■■■■■■	T	T1	T
612	■■■■■■■■■■■■■■■■■■■■■■■■■■■■■■■■□□□□■■■■□■■	T	T1	T
613	■■■■■■■■■□□■■■■■■■■■■■■■■■■■■■■■□□□□■■■■■■■	T	T1	T
614	■■■■■■■■■■■■■■■■■■■■□□□□■■■■■■■■□□□□■□□□■■■	LAM	LAM9	LAM

615	■■■■■■■■■■■■■■■■■■■■■■■■■■■■■■□■□□□□■■■■■■□	H	H3	H
616	■□□□□□□□□□□□□□□□□□□□□□□□□□□□□□□□□□■■■■■■■■■	U	U	Unassignable
617	■■■■■■■■■■■■□■■■■■■■■■■■■■■■■□■□□□□■□■■■■■■■■■	EAI	EAI5	EAI
618	■■■■■■■■■■■■□■■■■■■■■■■■■■■■■■■■■■□□□□■□■■■■■■■■■	EAI	EAI5	EAI
619	■■■■■■■■■■■■■■■■■■□□□□□□□■□□□□□□□□□□□□□□□■■	U	U	Unassignable
620	■■■■■■■■■■■■■■■■■■■■■■□□■□□□□□□■□□□□■■■■■■■	H	H1	T
621	□□□□□□□□□□□□□□□□□□□□□□□□□□□□□□□□□□■□■■■■■■■	BEIJ	BEIJ	BEIJ
622	■■■■■■■■■■■□□□□□□■■■■■■■■□□■□□□□■□■■■■■■■■■	EAI	EAI4	EAI
623	■■■■■■■■■■■■■■■■■■■■■■□■■■■■■■■■■■■■■■■■■■■	U	U	Unassignable
624	■□□■■■■■■■■■■■■■□■■■■■■■■■■■□□□□■□■■□□□■■■■	EAI	EAI3	EAI
625	■□□■■■■■■■■■■■■■■■■■■■■■■■■■□□□□■□■□□□□■■■■	EAI	EAI5	EAI
626	■□□■■■■■■■■■■■■■■■■■■■■■■■■■□□□□■□■□■■■■■■■	EAI	EAI5	EAI
627	■■■■□□□□■■■■□■■■■■■■■■■■■■■■■■■□□□□■■■■■■■	T	T3	T
628	■■■■■■■■■■■■■■■■■■■■■■■■■■■■■■■■□□□□■■■■■□□	T	T1	T
629	■□□■■■■■■■■■■■■■■■■■■■■■■■■■□□□□■□□□□□□■■■■	U	U	EAI
630	■■■■■■□□■■■■■■■■■■■■■□■■■■■■■■■■□□□□■■■■■■■	T	T1	T
631	■■■■■■■■■■■■■■■■■■■■■■■□■■■■■■□■□□□□■■■■■■■	H	H3	H
632	□□□□□□□□□□□□□□□□□□□□□□□□□□□□□□□□□□■■■□■■■■■	BEIJ	BEIJ	BEIJ
633	■■□■□■■■■■■■■■■■■■■■□□□□■■■■■■■■□□□□■■■□■■■	LAM	LAM4	LAM
634	□□□□□□□□□□□□□□□□□□□□□□□□■□□□□■■■■■■■■■□□□□□	animal	PIN	animal
635	□□□□□□□□□□□□□□□□□□□□□□□□■■■■□■■■□□□□■■■■■■■	T	T1	T
636	□□□□□□□□□□□□□□□□□□□□□□□□■■■■■■■■■■■■□□□□□□□	animal	BOV	Unassignable
637	□□□□□□□□□□□□□□□□□□□□□□□□■■■■■■■■■■■■■■□□□□□	animal	PIN	Unassignable
638	□□□□□□□□■■■■■■■■■■■■□□□□■■■■■■■■□□□□■■■■■■■	LAM	LAM9	LAM
639	□□□■□□□□□□□□□□□□□□□□□□□□□□□□□□□□□□□□■■□□□□□	animal	MICROTI	animal
640	□□□■■■■□□□□□□□□□□□□□□□□□■■■□□■■■■■■■□■□□□□□	animal	PIN	animal
641	□□□■■■■□□□□□□□□□□□□□□□■■□□□□□□□□□□□□■■□□□□□	animal	MICROTI	animal
642	□□□■■■■□□□□□□□□□□□□□□□■■□■□□□□□□□□□□■■□□□□□	animal	MICROTI	animal
643	□□□■■■■■■■■■■■■■■■□■■■■■■■■□□□□□□□□□□■■■■■■	U	U	Unassignable
644	□■□□□□□□□□□□□□□□■■□■■■■■■■■□■□□□□■■■■■□□□□□	animal	CAP	animal
645	□■□□□□□□□□□□□□□□■■■■■□□■■■■□■□□□□■■■■■□□□□□	animal	CAP	animal
646	□■□□□□□□□□□□□□□□■■■■■■■■■■■□■□□□□■■■■■□□□□□	animal	CAP	animal
647	□■□□□□□□□□□□□□□□■■■■■■■■■■■□■■■■■■■■■■□□□□□	animal	CAP	animal
648	□■□□□□□□□□□■□□□□■■■■■□□■■■■□■□□□□■■■■■□□□□□	animal	CAP	animal
649	□■■■■■■■■■■■■■■■■■■□□□■□□■■■□■■■□□□□■■■■■■■	T	T1	T
650	■□□□■■■■□■■■■■■□■■■■□■■■■■■■■■■■■■□□■■□□□□□	animal	BOV	animal
651	■□□■■□■□□□□□■■□□■■■■■■■■■■■■■■■■■■■■■■□□□□□	animal	BOV	AFRI
652	■□□■■■■■■■□■■■■■■■■■■■■■■■■■□□□□■□■■□□□■■■■	EAI	EAI3	EAI
653	■□□■■■■■■■■■■■■■■■■■□□□□■□□□□□□■□□□□■■■■■■■	LAM	LAM9	LAM
654	■□□■■■■■■■■■■■■■■■■■■□■■■■■■□□□□■□■■□□□■■■■	EAI	EAI3	EAI
655	■□□■■■■■■■■■■■■■■■■■■■■■■■■■■■□■□□□□■■■■■■■	H	H3	H
656	■□■■■■■■■■■■■■■■■■□■■■■■■■■■□□□■□□□□■■■■■■■	H	H4	T
657	■■□□□□□□□□□□□□□□□□□□□□□■■■□□□■■■■■■■■■□□□□□	animal	BOV	animal
658	■■□□□□□□□□□□□□□□□□□□□□□■■■■■■■■■■■■■■■□□□□□	animal	BOV	animal
659	■■□□□□□■□■■■□■■□■■■■■■■■■■■■■■■■■■■■■■□□□□□	animal	BOV1	animal
660	■■□□□□□■□■■■■■■□■■■■□□□□■■■■■■■■■■■■■■□□□□□	animal	BOV1	animal
661	■■□□□□□■□■■■■■■□■■■■■■■■■■■■■■■■■■■■■■□□□□□	animal	BOV1	animal
662	■■□□□■■□□□□□■■■□■■■■■■■■■■■■■■■■■■■■■■□□□□□	animal	BOV	animal
663	■■□□□■■■□■■■■■■□■■■■□■■■■■■■■■■■■■■■■■□□□□□	animal	BOV1	animal
664	■■□□□■■■□■■■■■■□■■■■■■■■■■□□■■■■■■■■■■□□□□□	animal	BOV1	animal
665	■■□□□■■■□■■■■■■□■■■■■■■■■■■■■■■■■■■■■■□□□□□	animal	BOV1	animal
666	■■□■□□□□□□□□□□■□■■■■■■■■■■■■□□■■■■■■■■□□□□□	animal	BOV3	animal
667	■■□■□□■□□□□□■■■□■■■■■■■■■■■■■■■■■■■■■■□□□□□	animal	BOV	animal
668	■■□■□■■■□■□□■■■□■■■■■□■■■■■■■■■■■■■■■■□□□□□	animal	BOV1	animal
669	■■□■□■■■□■□■■■■□■■□■■■■■■■■■■■■■■■■□■□□□□□	animal	BOV1	animal
670	■■□■□■■■□■□■■■■□■■□■■■■■■■■■■■■■■■■■■□□□□□	animal	BOV1	animal

671	■■□■■□■□□□□□■□□□■■■■■■■■■■■■■■■■■■■■■■□□□□□	animal	BOV	animal
672	■■□■■□■□□□□□■■□□■■■■■■■■■■■■■■■■■■■■■■□□□□□	animal	BOV	animal
673	■■□■■□■□□□□□■■■□□□□■■■■■■■■■■■■■■■■■■■□□□□□	animal	BOV	animal
674	■■□■■□■□□□□□■■■□□■■■■■■■■■■■■■■■■■■■■■□□□□□	animal	BOV	animal
675	■■□■■□■□□□□□■■■□■■□■■■■■■■■■■■■■■■■■■■□□□□□	animal	BOV2	animal
676	■■□■■□■□□□□□■■■□■■■■■■■■■□□□□□□■■■■■■■□□□□□	animal	BOV2	animal
677	■■□■■□■□□□□□■■■□■■■■■■■■■■■■■■■□□□□□■■□□□□□	animal	BOV2	animal
678	■■□■■□■□□□□□■■■□■■■■■■■■■■■■■■■□■■■■■■□□□□□	animal	BOV2	animal
679	■■□■■□■□□□□□■■■□■■■■■■■■■■■■■■■■□□■■■■□□□□□	animal	BOV2	animal
680	■■□■■□■□□□□□■■■□■■■■■■■■■■■■■■■■□■■■■■□□□□□	animal	BOV2	animal
681	■■□■■□■□□□□□■■■□■■■■■■■■■■■■■■■■■■■□■■□□□□□	animal	BOV2	animal
682	■■□■■□■□□□□□■■■□■■■■■■■■■■■■■■■■■■■■□■□□□□□	animal	BOV2	animal
683	■■□■■□■□□□□□■■■□■■■■■■■■■■■■■■■■■■■■■■□□□□□	animal	BOV2	animal
684	■■□■■□■■□■■■■■■□■■■■□■■■■■■■■■■■■■■■■■□□□□□	animal	BOV1	animal
685	■■□■■■■□□□□□■■■□■■■■■■■■■■■■■■■■■■■■■■□□□□□	animal	BOV	animal
686	■■□■■■■□□□□■■□■□■■■■■■■■■■■■■■■■■■■■■■□□□□□	animal	BOV	animal
687	■■□■■■■□□■□■■■■□■■■■■■■■■■■■■■■■□■■■■■□□□□□	animal	BOV	animal
688	■■□■■■■■□■□□■■■□■■■■■■■■■■■■■■■■■■■■■■□□□□□	animal	BOV1	animal
689	■■□■■■■■□■□■■■■□■■■■■■■■■■■□□□□□■■■■■■□□□□□	animal	BOV1	animal
690	■■□■■■■■□■□■■■■□■■■■■■■■■■■■■□□■■■■■■■□□□□□	animal	BOV1	animal
691	■■□■■■■■□■□■■■■□■■■■■■■■■■■■■■■■■■■■■■□□□□□	animal	BOV1	animal
692	■■□■■■■■□■■□■■■□■■■■□■■■■■■■■■■■■■■■■■□□□□□	animal	BOV1	animal
693	■■□■■■■■□■■■■□□□■■■■□■■■■■■■■■■■■■■■■■□□□□□	animal	BOV	animal
694	■■□■■■■■□■■■■□□□■■■■■■■■■■■■■■■■■■■■■■□□□□□	animal	BOV	animal
695	■■□■■■■■□■■■■■□□■■■■□■■■■■■■■■■■■■■■■■□□□□□	animal	BOV	animal
696	■■□■■■■■□■■■■■■□■■■■□■■■■□□□□■■■■■■■■■□□□□□	animal	BOV1	animal
697	■■□■■■■■□■■■■■■□■■■■□■■■■■□□□■■■■■■■■■□□□□□	animal	BOV1	animal
698	■■□■■■■■□■■■■■■□■■■■□■■■■■■■■■■■■■■■□■□□□□□	animal	BOV1	animal
699	■■□■■■■■■■■■■■■■■■■■■■■■■■■■■■□■□□□□■□■■■■■	H	H3	H
700	■■■□□□□□□■■■■□□□□■□□□□□□■■■■■■■■□■□□■■□■■■■	AFRI	AFRI	Unassignable
701	■■■□□□□□□■■■■□□□□■□□□□□□■■■■■■■■■■□□■■□■■■■	AFRI	AFRI	Unassignable
702	■■■□□□□□□■■■■■■■□■■■■■□□■■■■□□□□■□■■■■■■■■■	EAI	EAI5	EAI
703	■■■□□□□■■□□■■■■■■■■□□□□□□□□□□□□□□□□■■■■■■■■	U	U	CAS
704	■■■□□□□■■■■■■■■■■■■■■■□□□□□□□□□□□□■■□□■□□■■	CAS	CAS1	CAS
705	■■■□□■■■■□■■■■■■■■■■■■■■■■■■■■■■□□□□■■■■■■■	T	T1	T
706	■■■□■■□□□□□■■■■■■■■□■■■■■■■■■■■■□□□□■■■■■■■	T	T1	T
707	■■■□■■■■□□■■■■■■■■■■■■■■■■■■■■■■□□□□■■■■■■■	T	S	T
708	■■■□■■■■■■■■■■■■■□■■■■■■■■■■■■■■□□□□■■■■■■■	X	X1	X
709	■■■□■■■■■■■■■■■■■■■■□□□□■■■■■■■■□□□□■■■□□□□	LAM	LAM9	LAM
710	■■■□■■■■■■■■■■■■■■■■■□□□□□□□□□□□□□□□□□□□■■■	U	U	Unassignable
711	■■■□■■■■■■■■■■■■■■■■■■■■■■■■□□□□■□■■■■■□■■■	EAI	EAI1	EAI
712	■■■■□□■■■■■■■■■■■■■■■■■■■■■■■■■■□□□□■■■□■■■	T	T2	T
713	■■■■■□□■■■■■■■■■■■■■■■■■■■■■■■■■□□□□■■■■■■■	T	T1	T
714	■■■■■■■■■■■■■■■■■■□□□□□□■■□■■■□■□□□□■■■■■■■	H	H3	H
715	■■■■■■□□□■□□■■■■■■■■■■■■■■■■■■■■■■■■□■■■■	AFRI	AFRI1	Unassignable
716	■■■■■■□■□■■■■■■■■■■□□□□□□□□□□□□□□□□■■■□■■■■	AFRI	AFRI	animal
717	■■■■■■■□□□□■■■■■■■■■■■■■■■■■■■■■□□□□■■■■■■■	T	T1	T
718	■■■■■■■■□□□□□□□□□□□□□□□□□□□□□□□□□□□□■■■■■■■	U	U	Unassignable
719	■■■■■■■■□□□■■■■■■■■□□□□□■■■■■■■■□□□□■■■■■■■	T	T1	T
720	■■■■■■■■□□□■■■■■■■■■□□□□■■■■■■■■□□□□□■■■■■■	U	U	LAM
721	■■■■■■■■□□■■■■■■■■■■■■■■■■■■■■■■□□□□□■■■■■■	U	U	Unassignable
722	■■■■■■■■■■■■□□□□□□□□□□□□■■■■□□□□■□■■■■■□■■■	EAI	EAI1	EAI
723	■■■■■■■■■■■■□□□□□□□□□■■■■□□■□□□□■□■■■■■■■■■	EAI	EAI4	EAI
724	■■■■■■■■■■■■□□□□□■□□□□□□□■□□□□□□□□□■■■■□■■■	U	U	Unassignable
725	■■■■■■■■■■■■□■■■■■■■□□□□■■■■■■■■□□□□■■■■■■□	LAM	LAM5	LAM
726	■■■■■■■■■■■■□■■■■■■■■■■□□■■■■□□□□■□■■■■■■■■■	EAI	EAI5	EAI

727	■■■■■■■■■■■■□■■■■■■■■■■■■□□□□□□■□□□□■■■□■■■	H	H1	T
728	■■■■■■■■■■■■□■■■■■■■■■■■■■■■■■■□□□□■□□□■■■	T	T3	T
729	■■□■□■■■■■■■■■■■■■■■□□□□■■■■■■■■□□□□■■■■■■■	LAM	LAM1	LAM
730	■■■■■■■■■■■■■□■■■■■■■■■■■■■■□□□□■□■■■■■□■■■	EAI	EAI1	EAI
731	■■■■■■■■■■■■■■□□□■■■□□□□■■■■■■■■□□□□■■■■■■■	LAM	LAM9	LAM
732	■■■■■■■■■■■■■■□□■■■■■■■■■■■■■■■■□□□□■■■■■■■	T	T1	T
733	■■■■■■■■■■■■■■■■□■■■■■□□■■■■□□□□■□■■■■■■■■■	EAI	EAI5	EAI
734	■■■■■■■■■■■■■■■■■□■■■■■■■■■■□□□□■□□■■■■□■■■	U	U	Unassignable
735	■■■■■■■■■■■■■■■■■□■■■■■■■■■■□□□□■□■■■■■□■■■	EAI	EAI1	EAI
736	■■■■■■■■■■■■■■■■■■■□■■■■■■■■■■■■□□□□■■■□■■■	T	T2	T
737	■■■■■■■■■■■■■■■■■■■■□□□□■■■■■■■■□□□□■□■■■■■	LAM	LAM9	LAM
738	■■■■■■■■■■■■■■■■■■■■□□□□■■■■■■■■□□□□■■■■■□□	LAM	LAM9	LAM
739	■■■■■■■■■■■■■■■■■■■■■□□□■■■■□□□□■□■■■■■■■■■	EAI	EAI5	EAI
740	■■■■■■■■■■■■■■■■■■■■■■□□■■■■■■□■□□□□■■■■■■■	H	H3	H
741	■■■■■■■■■■■■■■■■■■■■■■□■■■■■■■□■□□□□■■■■■■■	H	H3	H
742	■■■■■■■■■■■■■■■■■■■■■■■■□□□□□□□■□□□□■■■■■■■	H	H3	T
743	■■■■■■■■■■■■■■■■■■■■■■■■■■□□□□□□□□□□□□□■■■■	U	U	Unassignable
744	■■■■■■■■■■■■■■■■■■■■■■■■■■■■□□□□■□□■■■■□■■■	U	U	EAI
745	■■■■■■■■■■■■■■■■■■■■■■■■■■■■□□□□■□■■□□■□■■■	EAI	EAI1	EAI
746	■■■■■■■■■■■■■■■■■■■■■■■■■■■■□■□■□□□□■■■■■■■	H	H3	H
747	■■■■■■■■■■■■■■■■■■■■■■■■■■■■■■□■□□□□■□□■■■■	H	H3	H
748	■■■■■■■■■■■■■■■■■■■■■■■■■■■■■■□■□□□□■■■■■□□	H	H3	H
749	■■■■■■■■■■■■■■■■■■■■□■■■■■■■■■■■■■■■■■■■■■■	U	U	Unassignable
750	□□□□■■■■■■■□■■■■■■■□■□□□□□□□□□■□□□□■■■■■■■	H	H3	T
751	□□□■■■■■■■■■■■■■■■■■■■■■■■■■■■■■□□□□■■■■■■■	T	T1	T
752	■□□■□□□□□□□□□□■□■■■■■■■■■■■■■■■■■■■■■■□□□□□	animal	BOV	animal
753	■□□■■■■■■■■■■■■■■■■■□□□□■■■■■■■■□□□□■■■■■■■	LAM	LAM9	LAM
754	■□■□□□□■■■■■■■■■■■■■■■□□□□□□□□□□□□■■■■■■■■■	CAS	CAS1	CAS
755	■□■■■■■■■■■■■■■■■■■■□■■■■■■■■■□■□□□□■■■■■■■	H	H3	H
756	■■□■■■□■■■■■■■■■■■■□□■■■■■■■□□□□■□■■■■■■■■■	EAI	EAI2	EAI
757	■■□■■■■■□■□■■■■□■■■■■■■■■■■■■□■■■■■■■■□□□□□	animal	BOV1	animal
758	■■□■■■■■■■■■■■■■■■■□□■■■■■■■□□□□■□■■■■■□□□□	EAI	EAI2	EAI
759	□□□■■■■■■■■□□■■■■■■■■■■■■■■■■■■■□□□□■■■■■■■	T	T1	T
760	■■■■■■■□□□□□□■■■■■□□□□□□□□□□□□□■□□□□■■■□□□□	H	H3	T
761	■■■■■■■□□□□□■■■■■■□□□□□□■■■■■■■■■■■■□□□■■■■	AFRI	AFRI3	AFRI
762	■■■■■■■□□■■■■■■■■■■■■■■■■■■■□□□■□□□□■■■□■■■	H	H4	T
763	■■■■■■■■■■■■□□□□□□■■■■■■■■■■□□□□■□■■■■■□□□□	EAI	EAI5	EAI
764	■■■■■■■■■■■■■□■■■■■■■■■■■■■■■■□■□□□□■■■■■■■	H	H3	H
765	■■■■■■■■■■■■■■□□□□□□□■■■■■■■■■■■□□□□■■■■■■■	T	T1	T
766	■■■■■■■■■■■■■■□□□■□□□□□□■■■■■■■■□□□□■■■■■■■	T	T1	T
767	■■■■■■■■■■■■■■■□■■■■■■■■■■■■□□□□■□■■■■■□□□□	EAI	EAI5	EAI
768	■■■■■■■■■■■■■■■■■■■□□□□□■■■■■■□■□□□□■■■■■■■	H	H3	H
769	■■■■■■■■■■■■■■■■■■■□□■■■■□□□□□□■□□□□■■■■■■■	H	H1	T
770	■■■■■■■■■■■□■■■■■■■■□□□□■■■■■■■■□□□□■■■■■■■	LAM	LAM9	LAM
771	■■■■■■■■■■■■■■■■■■■■■□□■■■■■■■■■□□□□■■■■■■■	T	T1	T
772	■■■■■■■■■■■■■■■■■■■■■■□□□■■■□□■■□□□□■■■■■■■	LAM	LAM10	T
773	■■■■■■■■■■■■■■■■■■■■■■■□□□□□□□□□□□□□□□□□■■■	U	U	Unassignable
774	■■■■■■■■■■■■■■■■■■■■■■■■□■■■■■■■□□□□■■■■■■■	T	T1	T
775	■■■■■■■■■■■■■■■■■■■■■■■■■■■□□□□□□□□□□■■■■■■	U	U	Unassignable
776	■■■■■■■■■■■■■■■■■■■■■■■■■■■■□□□□■□□□■■■■■■■	CAS	CAS	EAI
777	■■■■■■■■■■■■■■■■■■■■■■■■■■■■□□□■□□□□■■■■■■■	H	H4	T
778	■■□■■■■■□■□■■■■□■■■■■■■■■■■□□□■■■■■■■■□□□□□	animal	BOV1	animal
779	■■■■■■■■■■■■■■■■■■■■■□□■■■■■■■□■□□□□■■■■■■■	H	H3	H
780	■■■■■■■■■■■■■■■■■■■■■■■■■■■■■□□□□□□□■■■■■■■	U	U	Unassignable
781	□□□□□□□□□□□□□□□□□□□□□□□□■□□□□□□■□□□□■■■□■■■	H	H2	T
782	■□□□□■■■■■■■■■■■■■■■□□□□■■■■■■■■□□□□■■■■■■■	LAM	LAM9	LAM

783	■□□■■■■■■■■■■■■■■■■■■■■■■■■■■■■■□□□□■■■□■■■	T	T2	T
784	■■■■■■■■□□■■■■■■■■■■■■■■■■■■■■■■□□□□■■■□■■■	T	T2	T
785	■■■■■■■■■■■■■■■■■■■□■■□■■□□■■■■■□□□□■■■■■■■	T	T5	T
786	■■■■■■■■■■■■■■■■■■■■■■■□□□□□□□□□□□□□□□□□□□□	U	U	Unassignable
787	■■■■■■■■■■■■■■■■■■■■■■■■■■■■■■■■□□□□□□□■■■■	U	U	Unassignable
788	■□■■■■■■■■■■■■■■■■□□□□□□□■□□□□■■□□□□■■■■■■■	T	T1	T
789	■■□■■■■■□□■■■■■■■■■■■■■■■■■■■■■■□□□□■■■■■■■	T	S	T
790	■■■■■■■■□□■■■■■■■■■■■■■■■■■■■■■□□□□□■■■■■■■	U	U	Unassignable
791	■■■■■■■■■■■■■■■■■■■■■■■□□□□□□□□■□□□□■■■■■■■	H	H3	T
792	■■■■■□□■■■■■■■■■■■■■■■■■■■■■□□□□■□■■■■■■■■■	EAI	EAI5	EAI
793	■□■■■■■■■■■■□■■■■□■■■■■■■■■■■■■■□□□□■■□□□□■	X	X2	X
794	■■■□□□□■■■■■■□■■■■■■■■□□□□□□□□□□□□■■■■■■■■■	CAS	CAS1	CAS
795	□□□□□□□□□□□□□□□□□□□□□□□□■■□■■■■■□□□□■■■■■■■	T	T1	T
796	□□□□□□□□□□□□□□□□□□□□□□□□□□□□□□□□□□□■■■■■■■■	BEIJ	BEIJ-LIKE	Unassignable
797	■■□■■■■■□■■■■■■□■■■■■■■■■■■■■■■■■■■■□■□□□□□	animal	BOV1	animal
798	■□□□■■■■■■■■■■■■■■■■■■■■■■■■■■■■□□□□■■■■■■■	T	T1	T
799	■■■■■■□□□■■■■■□■■■■■□■■■■■■■■■■■■■■■■■□■■■■	AFRI	AFRI1	Unassignable
800	■■■■■■■□□□□□□□□□□□□■■■■■□□□□□□□■□□□□■■■■■■■	H	H3	T
801	■■■■■■■□□■■■■■■■■■■■■■■■■■■■■■■■□□□□■■■■■■■	T	T1	T
802	■■■■■■■■■■■□□□□□■■□□□□□□□□□□□□□□□□□□□□□□□■■	U	U	Unassignable
803	■■■■■■■■■■■■■□□□□□□□□□□□■■■■■■■■□□□□■■■■■■■	T	T1	T
804	■□□■■■■■■■■■■■■■■■■■■■■■■■■■■■■■□□□□■■■■■■■	T	T1	T
805	■■■□■■■■■■□□□□■■■■■■□□□□■■□□□□■■□□□□■■■□■■■	LAM	LAM11	LAM
806	■■■■□■■■■■■■■■■■■■■■■■■■■■■■□□□□■□■■■■■□■■■	EAI	EAI1	EAI
807	■■■■■■□□□□□□■■■■■■■■□□□□■■□□□□■■□□□□■■■□■■■	LAM	LAM11	LAM
808	■■■■■■■■■■■■■□□■■■■■□□□□■■□□□□■■□□□□■■■■■■■	LAM	LAM11	LAM
809	■■■■■■■■■■■■■■■■□■■■□□□□■■□□□□■■□□□□■■■■■■■	LAM	LAM11	LAM
810	■■■■■■■■■■■■■■■■■■■■□□□□□■□□□□■■□□□□■■■■■■■	T	T1	T
811	■■■■■■■■■■■■■■■■■■■■□□□□■□□□□□■■□□□□■■■□■■■	LAM	LAM4	LAM
812	■■■■■■■■■■■■■■■■■■■■□□□□■■□□□□■■□□□□■□■■■■■	LAM	LAM11	LAM
813	■■■■■■■■■■■■■■■■■■■■□□□□■■□□□□■■□□□□■■□□■■■	LAM	LAM11	LAM
814	■■■■■■■■■■■■■■■■■■■■□□□□■■□□□□■■□□□□■■□■■■■	LAM	LAM11	LAM
815	■■■■■■■■■■■■■■■■■■■■□□□□■■□□□□■■□□□□■■■□■■■	LAM	LAM11	LAM
816	□□□■■■■■■■■■■■■■■■■■□□□□■■□□□□■■□□□□■■■■■■■	LAM	LAM11	LAM
817	■■■■■■■■■■■■■■■■■■■■■■■■■■■■□□□■□□□□■■■□■■■	H	H4	T
818	□■□□□□□□□□□□□□□□■■■■■■■■□□□□□□□□□□□□□□□□□□□	animal	CAP	Unassignable
819	■■□■■■■■□■■■■■□□■■■■□■■■■■■■■■■■■■■■□□□□□□	animal	BOV	animal
820	■■□■■■■■□■■■■■□□■■■■■■■■■■■■■■■■■■■■■■□□□□□	animal	BOV	animal
821	■■□■■■■□□□□□□□□□□□□□□□□□□□□□□□□□□□□□□□■■■■■	U	U	animal
822	■■■■□■■■■■■■■■■■■■■■□□□□■■■■■■■■□□□□■■■■■■■	LAM	LAM9	LAM
823	■■■■■■■■□□□□□□□□□□□□□□□□□■■■■■■■□□□□■■■■■■■	T	T1	T
824	■■■■■■■■■■■■■■■■■■■□□■□■■■■■■■■■□□□□■■■■■■■	T	T5	T
825	■■■■■■■■■■■■■■■■■■■■□□□□□□□□□□□□□□□□□□■■■■■	U	U	Unassignable
826	■■□■■■■■■■■■□■■■■■□■□□□□■■■■■■■■□□□□■■■■■■■	LAM	LAM2	LAM
827	■■■■■■■■□□■■□■■■■■■■■■■■■□□□■■■■■□□□□■■■■■■■	T	S	T
828	□■■■■■■■■■■■■■■■■■■■□□□□■■■■■■■■□□□□■■■□■■■	LAM	LAM4	LAM
829	■■□■□□□□□□□□□□□□■■■■■■■■■■■■■■■■■■■■■■□□□□□	animal	BOV	animal
830	□□□□□□□□□□□□□□□□□□□□□□□□■■■■■■□■□□□□■■■□■■■	H	H3	H
831	■■■■■■■■□□■■■■□■■■■■■■■■■■■■■■■■□□□□■■■■■■■	T	S	T
832	■■■■■■■■■■■■□■□■■■■■■■■■■■■■■■■■□□□□■■■□■■■	T	T2	T
833	■■■■□■■■■■■■■■■■■■■■■■■■■■■■■■■■□□□□■■■□□□□	T	T1	T
834	■■■■■■■■■■■■■■□■■■■■■■■■■■■■■■■□□□□□■■■■■■■	U	U	Unassignable
835	■■■■■■■■■■■■■■□■■■■■■■■■■■■■■■□■□□□□■■□■■■■	H	H3	H
836	■■■■■■■■■■■■■■■■■■■■■■■■■■■■■■□□□□□□□□■□■■■	U	U	Unassignable
837	■■■■■■■■■■■■■■■■■■■■■■■■■■■□□□□■□□□□■■■□■■■	H	H3	T
838	■■■■■■■■■■■■■■■■■■■■■■□□□■■■■■■■□□□□■■■■□■■	LAM	LAM10	T

839	■■■■■■■■■■■■■■■■■■■■■■□□□■■■■■□□□□□□□□■■■■■	U	U	Unassignable
840	■■■■■■■■■■■■■■■■□■■■■■■■■■■■■■■□■□□□□■■■■■■■	H	H3	H
841	■■■■■■■■■■■■■■□■■■■■□■□□□■■■■■■■□□□□■■■■■■■	LAM	LAM10	T
842	■■■■■■■■■■■■■■□■■□■■■■■■□□□□□□□□□□□□□□□□□□□	U	U	X
843	■■■■■■■■■□□■■■■■■■■■■■■■■■■■■■□■□□□□■■■■■■■	H	H3	H
844	■■■■■□□□■■■■■■■■■■■■■■□□□■■■■■■■□□□□■■■□■■■	LAM	LAM10	T
845	■■■■■□□□■■■■■■□■■■■■■■□□□■■■■■■■□□□□■■■□■■■	LAM	LAM10	T
846	■■■■■□□□□■■■■■■■■■■■■■■■■■■■■■■■□□□□■■■□■■■	T	T2	T
847	■■■■■□□□□□□□□□□□□■■■■■□□□■■■■■■■□□□□■■■■■■■	LAM	LAM10	T
848	■■■□■■■■■■■■■■■■■■■■■■■■■■■■■■■■□□□□■■■□■■■	T	T2	T
849	■■□□■■■■■■■■■■■■■■■■■■■■■■■■■■□■□□□□■■■■■■■	H	H3	H
850	■□□□■■■■■■■■■■■■■■■■■■□□□■■■■■■■□□□□■■■■■■■	LAM	LAM10	T
851	■□□□□□□■■■■■■■■■■■■■■□□□□■□□□□□□□□□□□■■■■■■	U	U	Unassignable
852	■□□□□□□□□□□□□□□□■■■■■■□□□■■■■■■■□□□□■■■■■■■	LAM	LAM10	T
853	□■■■■■■■■■■■■■■■■■■■■■■■■■■■■■■□□□□■■■□■■■	T	T2	T
854	□□■■■■■■■■■■■■■■■■■□□□□□□■■■■■■■□□□□■■■■■■■	T	T1	T
855	□□□□■■■■■■■■■■□■■■■■■■■■■■■■■■■□■□□□□■■■□■■■	H	H3	H
856	■■■■■■■□□□□□■■■■■■■■■■■■□□□□□□□□□■□■□□□■■■■	AFRI	AFRI3	AFRI
857	■■■■■■■□□□□□■■□■■■■■■■■■□□□□□□□□□■■■□□□■■■■	AFRI	AFRI3	AFRI
858	■■■■■■■□□□□□■■□■■■■■■■■■□□□□□□□□□■□■□□□■■■■	AFRI	AFRI3	AFRI
859	■■■■■■□□□■■■■■■■■■■■■■■■■■■■■■□□■■■□□□■■■■	AFRI	AFRI	AFRI
860	■□■■■■■□□□□□■■□■■■■■□□□□■■■■■■■■■■■■□□□■■■■	AFRI	AFRI2	AFRI
861	■□■■■■■□□□□□■■□■■■■■□□□□■■□■■■■■□■■■□□□■■■■	AFRI	AFRI2	AFRI
862	■■■■■■■■■■■■■■□■■■■■■■■■■■■□■■■■□□□□■■■□■■■	T	T2	T
863	□□□□□□□□□□□□□□□□□□□□□□□□■■■■□□□□□□□□□□□□□□□	U	U	EAI
864	■■■□□□□■■■■■■□□□□□□□□□□□□□□□□□□□□□□■■■■■□□	CAS	CAS	CAS
865	■□□■■■■■■■■■■■■■■■■■□■■■■■■■□□□□■□■■□□□■■■■	EAI	EAI3	EAI
866	■□■■■■■■■■■■■■■■■■■■□□□□■■■■■■■■□□□□■■■■■■■	LAM	LAM9	LAM
867	■■■■■■■■■■■■□■■■■■■■□□□□■■■■□■■■□□□□■■■□■■■	LAM	LAM5	LAM
868	■■■■■■■■■■■□■■■■■□■■■■■■■■■■■■■■□□□□■■■■■■■	X	X1	X
869	■■■■■■■■■■■■■■■■□□□□□■□□■■■■□■■■□□□□■■■■■■■	T	T1	T
870	■■■■■■■■■■■■■■□■■■■■■■■■■■■□■■■■■□□□□■■■□■■■	T	T2	T
871	■■■□■■■■■■■■■■■■■■■■■■■■■■■■■■□■□□□□■■■■■■■	H	H3	H
872	■■■■■■□■■■■■■■■■■■■■■□□■□□■■■■■■■□□□□■■■■■■■	T	T1	T
873	■■■□□□□■■■□□□□□□□□□□□□□□□□□□□□□□□□□□□□□□□□□	CAS	CAS	CAS
874	■■■■■■■■□□■■■■■■■■■■■■■■■■■■■■■■□□□□■□□□■■■	T	S	T
875	■■■■■■■■■■■■□□■■■■■■■■■■■■■■■■■■□□□□■■■□■■■	T	T2	T
876	■■■□□□□■■■■■■■■■■■■■■■□□□□□□□□□□□□□□■■■□■■■	CAS	CAS	CAS
877	■■■■■■□□□□□□□□□□□□■■■■■■□□□□□□□□□□□□□□□□□□□	U	U	Unassignable
878	■■■■■■■■■■■■■■■■■□■■■■■■■■■■■■■■□□□□■□■■■■■	X	X1	X
879	■■■■■■■■■■■■■■□■■■■■■■■■■■■■■■■■□□□□■■□■■■■	T	T1	T
880	■■■■■■■■■■■■■■■■■■□□□□□□□□□■■■■■□□□□■■■■■■■	T	T1	T
881	■■■■■■■■□□■■■■■■■■■■■■■■□□□□□□□□□□□□■■■□■■■	U	U	Unassignable
882	■□■■■■■■■■■■■■■■■■■■■■□■■■■■■□□□□■□■■■■■■■■■	EAI	EAI6	EAI
883	■■■■■■■■■■■■■■■■■■■■■■□■■□□□□□□■□□□□■■■■■■■	H	H1	T
884	□■■■■■■■□□■■■■■■■■■■■■■■■■■■■■■■□□□□■■■■■■■	T	S	T
885	■■■■■■■■■■■■□■■■■■■■□□□□■■■■□□■■□□□□■■■■■■■	LAM	LAM5	LAM
886	■■■■■■■■■■■■■■■■■□■■■■■■■■□■■■■■□□□□■■□□□□■	X	X2	X
887	■□■■□■■■■■■■■■□■■■■■■■■■■■■■■■□■□□□□■■■■■■■	H	H3	H
888	■■■■■■■■■■■■■■■■■■■■■■■■■■■■■■■■□□□□■■□□■■■	T	T1	T
889	■■■■□■■■■■■■■■■■■■■■■■□■■■■■□□□□■□■■■■■■■■■	EAI	EAI6	EAI
890	■■■■□□□□■■■■■■■■□□□□□■□□■■■■□■■■□□□□■■■■■■■	T	T1	T
891	■■■■■■■■■■■■■■■■■■■■□□□□■■■■■□■■□□□□■■■■■■■	LAM	LAM9	LAM
892	■■■■■■■■■■■■■■■■■■■□□■■■■■■■□□□□■□■■■■■■■■■	EAI	EAI5	EAI
893	■■■■■■■■■■■■■■■■■■□□□□□■■■■■■■■■□□□□■■■■■■■	T	T1	T
894	■■□■■■■■■■■■■■■■■■■□□■■■■■■■□□□□■□■■■■■□■■■	EAI	EAI2	EAI

895	■■□■■■■■■■■■■■■■■■■□□■■■■■■□□□□□■□■■■■■■■■■	MANU	MANU1	MANU
896	■■■■■■■■■■■■□■■■□□□□□■■■■■■■□■■■□□□□■■■■■■■	T	T3	T
897	■■□■■■■■□□□□□□□□■■■□□■■■■■■■□□□□■□■■■■■■■■■	EAI	EAI2	EAI
898	■■□■■■■■■■■■■■■■■■■■■■■■■□□□□□□■□□□□■■■■■■■	H	H1	T
899	■■□■■■□□□□□□□□□□□□■■■■■■■■■■■■■■□□□□■■■■■■■	T	T1	T
900	■■□■■■■■■■■■■■■■■■■■□□□□■■■■■■■□□□□□□□□□□■■	U	U	LAM
901	■■■□□□□□□□□□□■■■■□■■■■■■■■■■■■■□□□□□□□□□■■■	U	U	X
902	■■■■■■■■□□□■□■■■■■■■□□□□■■■■■■■■□□□□■■□□■■■	LAM	LAM3	LAM
903	■■■■■■■■■■■■■■■□■□■■■■■■■■■■■■■■□□□□■■□□□□■	X	X2	X
904	■■■■■■■□■■■■■■■■□■■■■■□■■■■■■■■■□□□□■■■■■■■	T	T5	T
905	■■□■■■■■■■■■□■■■■■■■□□□□■■■■■■□□□□□□■■■■■■■	U	U	LAM
906	■■■■■■■■■■■■■■■■□□□□■■■■■■■■■■□■□□□□■■■■■■■	H	H3	H
907	■■■■■■■■■■■■■■■■■■■■□■■■■■■■■■■■□□□□■■■■■■□	T	T1	T
908	■■□■■■■■■■■■□■■■■■■■□□□□■■■■■■■■□□□□■■■□□□□	LAM	LAM2	LAM
909	■■■■□□□□□■■■■■■■■■■■□■■■■■□□□□□□□□□□□□□□□■■	U	U	Unassignable
910	■■■□□□□□□□□□□□□□□□□□□□□□■■■□□■■■■■■■■■■■■■■	U	U	Unassignable
911	■■■■■■■■■■■■■■■■■■■■□□□□□□□□□□■■□□□□■■■■■■■	T	T1	T
912	■■□■■■■■■■■■■■■■■■■■■■■■■■■■□□□□■□■■□■■■■■■	EAI	EAI5	EAI
913	■■■■■■■■■■■■■□□□■■■■■■■■■■■■■■■■□□□□■■■■■■■	T	T1	T
914	■■■■■■■■□□■■■■■■■■■■■■■■■■■■■■□■□□□□■■■■■■■	H	H3	H
915	■■■■■□□□■■■■■■■■■■■■■■■■■■■■■■□■□□□□■■■□■■■	H	H3	H
916	■■■■■■■■■■■■■■■■■■■■□□□□■■■■■■■■□□□□■□□□□■■	LAM	LAM9	LAM
917	■■■■■■■■■■□■■■■■■■■■■■■■■■■■■■■■□□□□■■■■■■■	T	T1	T
918	■■■■■■□□□□□□□□□□□□■■□□□□■■■■■■■■□□□□■■■■■■■	T	T1	LAM
919	■■■■■■■■■■■■■■■■■■■■■■■■■□□□□□□■□□□□■■■■■□□	H	H1	T
920	■■■■■■■■■■■□□■■□■■■■■■■■■■■■□□□■□□□□■■■■■■■	H	H4	T
921	■□□□■■■■■■■■□■■■■■■■■■■■■■■■□□□■□□□□■■■■■■■	H	H4	T
922	■■■■■■■□□■■■■□□■■■□■■■■■■■■■□□□■□□□□■■■■■■■	H	H4	T
923	■■□■■■■■□□■■■■■■■■■■■■■■■■■■■■□■□□□□■■■■■■■	H	H3	H
924	■■■■■■■■■■■□□□□□□□□□□□□□■■■■□□□□■□■■□■■■■■■	EAI	EAI5	EAI
925	■■■■■■■■■■■■□□■■■■■■■■■■■■■■■■□■□□□□■■■■■■■	H	H3	H
926	■■■■■■□■■■■■■■■■■■■■■■■■■■■■■■■■□□□□■■■■■■■	T	T1	T
927	■□□■□□□□□□□□■■□□□□□□□□□□□□□□□□□□□□□□■■■■■■■	U	U	Unassignable
928	■■■■■■■■■■■■■■■■■■■■■■□■□□□□□□■■□□□□■■■■■■■	T	T5	T
929	■■■■■■■■■■■■□■■■■■■■■■■■■■■■■□□■□□□□■■■■■■■	H	H3	T
930	■■□■■■■■■■■■■■■■■■■□□□□□■□□■■■■■□□□□■■■■■■■	LAM	LAM7	T
931	■■■■■■■□□□□□□□□□□□□□□□□□■■■■■■■■□□□□■■■■■■■	T	T1	T
932	■■■■■■■■■■■■■■■■■■■■□■■□□■■■■■■■□□□□■■■□■■■	T	T2	T
933	■■■■■■■■■■■■■■■■■■■■□□□□■■■■■■■■□□□□□□□■■■■	U	U	LAM
934	■■■■■■■■■■■■■■■■■■■■■■■■■■■■□□□□■□■■□□□■■■■	EAI	EAI5	EAI
935	■■■■■■■□■■■■■■■■■■■■■■■■■■■■■■□■□□□□■■■■■■■	H	H3	H
936	■■■■□□□□□■■■□■■■■■■■□■■■■■■■■■■■□□□□■■■□■■■	T	T2	T
937	■■■■■■■■■■■■■■■■■■■■■■■■■■■□□□□□■□■■■■■■■■■	MANU	MANU1	MANU
938	■□□■■■■□□□□■■■■■■■■■■■■■■■■■□□□□■□■■■■■■■■■	EAI	EAI5	EAI
939	■■■■■■■□■■■■■■■■■■■■■■■■■■■■□□□□■□■■■■■■■■■	EAI	EAI5	EAI
940	□□□□□□□□□□□□□□□□□□□□□□□□□□□□□□□□□□■■■□□□□□■	BEIJ	BEIJ	BEIJ
941	□□□□□□□□□□□□□□□□□□□□□□□□□□□□□□□□□□■■■■■■□■■	BEIJ	BEIJ	BEIJ
942	■■■■■■■■■■■■■■■■■■■■■■■■□□□■■■■■□□□□■■■□■■■	T	T2	T
943	■■■■■■■■■■■■■■■■■■■■■■□■■■■■■■■■□□□□■■■□■■■	T	T2	T
944	■■■■■■■■■■■■■■■■■■■■■■■■■□□□□□□□□□□□□□□■■■■	U	U	Unassignable
945	■■■■■□□■■■■■■□□□■■■■■■■■■■■■□□□□■□■■■■■■■■■	EAI	EAI5	EAI
946	■■■■■■■■■■■■■■■■■■■■■■□□□□□□□□□■□□□□■■■■■■■	H	H3	T
947	■■■■■■■■■■■■■■■■■■■■■■■■■■■■□□□□■□■■■■□□■■■	EAI	EAI5	EAI
948	■■■■■■■■■■■■■■■■■■■■■■■□□□□□□□□■□□□□■■□□□■■	H	H3	T
949	■■■■■■■■■■■■■■■■■■□□□□□■■■■■■■□■□□□□■■■■■■■	H	H3	H
950	■■■□□□□□□□□□□■■■■■■■□□□□■■■■■■■■□□□□■■■■■■■	LAM	LAM9	LAM

951	■■■■■■■□□□□■■■■■■■■■■■■■■■■■□□□□■□■■■■■■■■■	EAI	EAI5	EAI
952	■■□□□□□■■■■■■■■■■■■■■■□□□□□□□□□□□□■■■■■■■■■	CAS	CAS	CAS
953	■■■■■■■■■□■■■■■■■■■□■■■■■■■□■■■■□□□□■■■■■■■	T	T1	T
954	■■■□□□□■■■■□■■■■■■■■■■□□□□□□□□□□□□■■■■■■■■■	CAS	CAS1	CAS
955	□□□□□□□□□□□□□□□□□□□□□□□□■■■■■■■■■■■■■■■■■■■	U	U	Unassignable
956	■■■■■■■■■■■■■■■■■■■■■■■■■■■■■■■■□□□□□□□□□■■	U	U	Unassignable
957	■■□■■■■■□■□■□□□□□■■■■■■■■■■□□■■■■■■■■■□□□□□	animal	BOV	animal
958	■■■■■■■■■■■■■■■■■■■■■■■■■■□□□□□□■□■■■■■□■■■	MANU	MANU1	MANU
959	■■■■■■■■■■■■■□□□□■■■■■■■■■■■□□□□■□■■■■■□■■■	EAI	EAI1	EAI
960	■□□■■■■■■■■■□■■■■■■■□□□□■■■■■■■■□□□□■■■■■■■	LAM	LAM5	LAM
961	■□□□□□■■■■■■■■■■■■■■□□□□■■■■■■■■□□□□■■■■■■■	LAM	LAM9	LAM
962	■■■■■■■■■■■■■■■■■■■■■■■■■■■■□□□□■□■■□□□□■■■	EAI	EAI5	EAI
963	■■■■■■■■■■■■■■■■■■■■■□■■■■■■■■□■□□□□■■■■■■■	H	H3	H
964	■□□□■■■■■■■■■■■■■■■■□□□□■■■■■■■■□□□□■■■■■■■	LAM	LAM9	LAM
965	■□□□■■■■■■■■■■■■■■■□□□□□■■■■■■■■□□□□■■■■■■■	T	T1	T
966	■■■■■■■□■■■■■■■■■■■■■■■■■■■■■■■■□□□□■■■■■■■	T	T1	T
967	□□□□□□□□□□□□□□□□□□□□□□□□■■□□□■■■■■■■■■□□□□□	animal	PIN	animal
968	□□□□□□□□□□□□□□□□□■■■■□□□■□■■■■■■■■■■■■□□□□□	animal	BOV	animal
969	□□□□□□□□□□□□□□□□□■■■■□□□■■■■■■■■■■■■□□□□□□□	animal	BOV	Unassignable
970	□□□□□□□□□□□□□□□□□■■■■□□□■■■■■■■■■■■■■■□□□□□	animal	BOV	animal
971	□□□□□□□□□□□□□□□□□■■■■■□□■■■■■■■■■■■■□□□□□□□	animal	BOV	Unassignable
972	□□□□□□□□□□□□□□□□□■■■■■□□■■■■■■■■■■■■■■□□□□□	animal	BOV	animal
973	□□□□□■■■□■■■■■■□■■■■■■■■■■■■■■■■■■■■■■□□□□□	animal	BOV	animal
974	□□□■□□■■□■■■■■■□■■■■■■■■■■■■■■■■□■□□■■□□□□□	animal	BOV	animal
975	□□□■□□■■□■■■■■■□■■■■■■■■■■■■■■■■□■■■■■□□□□□	animal	BOV	animal
976	□□□■■■■■□■■■■■■□■■■■■■■■■■■■■■■■■■■■■□□□□□□	animal	BOV	animal
977	□□□■■■■■□■■■■■■□■■■■■■■■■■■■■■■■■■■■■■□□□□□	animal	BOV	animal
978	□■□□□□□□□□□□□□□□■■■■■■■■■■■□■■■□■■■■■■□□□□□	animal	CAP	animal
979	□■□□□□□□□□□□□□□□■■■■■■■■■■■■■■■■■■■■■■□□□□□	animal	CAP	animal
980	■□□□□□□□□□□□□□□□□□□□□■■■■■■■■■■■■■■■■■□□□□□	animal	BOV	animal
981	■□□□□■■■□■■■■■■□■■■■■■■■■■■■■■■■■■■■□□□□□□□	animal	BOV	animal
982	■□□□□■■■□■■■■■■□■■■■■■■■■■■■■■■■■■■■■■□□□□□	animal	BOV	animal
983	■■□□□□□□□□■■■■■□■■■■■■■■■■■■■■■■■■■■■■□□□□□	animal	BOV	animal
984	■■□□□□□■□■■■■□■□■■□■■■■■■■■■■■■■■■■■■■□□□□□	animal	BOV1	animal
985	■■□□□□■■□■■■■■■□■■■■□■■■■■■■■■■□□■■■■■□□□□□	animal	BOV1	animal
986	■■□□□□■■□■■■■■■□■■■■■■■■■■■■■■■■■■■■■■□□□□□	animal	BOV1	animal
987	■■□□□■■□□□□■■■■□■■■■■■■■■■■■■■■■■■■■■■□□□□□	animal	BOV	animal
988	■■□□□■■■□■■□□□□□□■■■■■■■■■■■■■■■■■■■■■□□□□□	animal	BOV	animal
989	■■□□□■■■□■■■□■■□■■■■■■■■■■■■■■■■■■■■■■□□□□□	animal	BOV1	animal
990	■■□□□■■■□■■■■■■□■□■■■■■■■■■■■■■■■■■■■■□□□□□	animal	BOV1	animal
991	■■□□□■■■□■■■■■■□■■■■■□■■■■■■■■■■■■■■■■□□□□□	animal	BOV1	animal
992	■■□□□■■■□■■■■■■□■■■■■■■■■■■■■□■■■■■■■■□□□□□	animal	BOV1	animal
993	■■□□□■■■□■■■■■■□■■■■■■■■■■■■■■■□■■■■■■□□□□□	animal	BOV1	animal
994	■■□□□■■■□■■■■■■□■■■■■■■■■■■■■■■■□□■■■■□□□□□	animal	BOV1	animal
995	■■□□□■■■□■■■■■■□■■■■■■■■■■■■■■■■■■□■■■□□□□□	animal	BOV1	animal
996	■■□□■■■■□□■■■■■□■■■■□■■■■■■■■■■■■■■■■■□□□□□	animal	BOV	animal
997	■■□□■■■■□■■■■■■□■■■■■■■■■■■■■■■■■■■■■■□□□□□	animal	BOV1	animal
998	■■□■□□■■□■■■■■■□■■■■■■■■■■■■■■■■□■■■■■□□□□□	animal	BOV	animal
999	■■□■□□■■□■■■■■■□□□□□□■■■■■■■■■■■□■■■■■□□□□□	animal	BOV	animal
1000	■■□■□□■■□■■■■■■□■■■■■■■■■■□□□■■■□■■■■■□□□□□	animal	BOV1	animal
1001	■■□■□□■■□■■■■■■□■■■■■■■■■■■■■■■■□■■■□■□□□□□	animal	BOV1	animal
1002	■■□■□□■■□■■■■■■□■■■■■■■■■■■■■■■■□■■■■■□□□□□	animal	BOV1	animal
1003	■■□■□■■■□■■■■■■□■■■■■■■■■■■■■■■■■■■■■■□□□□□	animal	BOV1	animal
1004	■■□■■□□■□■■■■■■□■■■■■■■■■■■■■■■■■■■■■■□□□□□	animal	BOV1	animal
1005	■■□■■■□■□■■■■■■□■■■■■■■■■■■■■■■■■■□■■■□□□□□	animal	BOV1	animal
1006	■■□■■■■□□□■■■■■□■■■■■■■■■■■■■■■■■■□□□□□□□□□	animal	BOV	animal

1007	■■□■■■■□□□■■■■■□■■■■■■■■■■■■■■■■■■■■■■□□□□□	animal	BOV	animal
1008	■■□■■■■■□■□■■■□□■■■■■■■■■■■■■■■■■■■■■■□□□□□	animal	BOV	animal
1009	■■□■■■■■□■■■□□■□■■■■□■■■■■■■■■■■□■■■■■□□□□□	animal	BOV1	animal
1010	■■□■■■■■□■■■□■■□■■■■□■■■■■■■■■■■■■■■■■□□□□□	animal	BOV1	animal
1011	■■□■■■■■□■■■■□□□■■□■■■■■■■■■■■■■■■■■■■□□□□□	animal	BOV	animal
1012	■■□■■■■■□■■■■■□□□□□□□□□□□□□□□□□□□■■■■■□□□□□	animal	BOV	animal
1013	■■□■■■■■□■■■■■□□□□□□□□□□■■□□□□■■□■■■■■□□□□□	animal	BOV	animal
1014	■■□■■■■■□■■■■■□□■■■■□□■■■■■■■■■■□■■■■■□□□□□	animal	BOV	animal
1015	■■□■■■■■□■■■■■■□□□□□□■■■■■■■■■■■■■■■■■□□□□□	animal	BOV	animal
1016	■■□■■■■■□■■■■■■□■□■■■■■■■■■■■■■□■■■■■□□□□□	animal	BOV1	animal
1017	■■□■■■■■□■■■■■■□■■□□□□□■■■■■■■□■■■■■■■□□□□□	animal	BOV1	animal
1018	■■□■■■■■□■■■■■■□■■□■■■■■■■■■■■■□■■■■■■□□□□□	animal	BOV1	animal
1019	■■□■■■■■□■■■■■■□■■□■■■■■■■■■■■■■■■■■■■□□□□□	animal	BOV1	animal
1020	■■□■■■■■□■■■■■■□■■■□□□□■■■■■■■■■■■■■■■□□□□□	animal	BOV1	animal
1021	■■□■■■■■□■■■■■■□■■■■□■■■■□□□□□□■■■■■■■□□□□□	animal	BOV1	animal
1022	■■□■■■■■□■■■■■■□■■■■□■■■■■■■■■■□□□■■■■□□□□□	animal	BOV1	animal
1023	■■□■■■■■□■■■■■■□■■■■□■■■■■■■■■■□■■■■■■□□□□□	animal	BOV1	animal
1024	■■□■■■■■□■■■■■■□■■■■□■■■■■■■■■■■□□□■■■□□□□□	animal	BOV1	animal
1025	■■□■■■■■□■■■■■■□■■■■□■■■■■■■■■■■□□■■■■□□□□□	animal	BOV1	animal
1026	■■□■■■■■□■■■■■■□■■■■□■■■■■■■■■■■■■□□■■□□□□□	animal	BOV1	animal
1027	■■□■■■■■□■■■■■■□■■■■□■■■■■■■■■■■■■□■■■□□□□□	animal	BOV1	animal
1028	■■□■■■■■□■■■■■■□■■■■□■■■■■■■■■■■■■■■□□□□□□□	animal	BOV	animal
1029	■■□■■■■■□■■■■■■□■■■■■□■■■■■■■■■■□■■□■■□□□□□	animal	BOV1	animal
1030	■■□■■■■■□■■■■■■□■■■■■□■■■■■■■■■■■■■■■■□□□□□	animal	BOV1	animal
1031	■■□■■■■■□■■■■■■□■■■■■■■□■□□□□■■□□■■□■■□□□□□	animal	BOV1	animal
1032	■■□■■■■■□■■■■■■□■■■■■■■■■■□□□□□□□□■■■■□□□□□	animal	BOV1	animal
1033	■■□■■■■■□■■■■■■□■■■■■■■■■■■□□□■■■■□■■■□□□□□	animal	BOV1	animal
1034	■■□■■■■■□■■■■■■□■■■■■■■■■■■■■□□□□□□□■■□□□□□	animal	BOV1	animal
1035	■■□■■■■■□■■■■■■□■■■■■■■■■■■■■□□■□■■■■■□□□□□	animal	BOV1	animal
1036	■■□■■■■■□■■■■■■□■■■■■■■■■■■■■□□■■■■■■■□□□□□	animal	BOV1	animal
1037	■■□■■■■■□■■■■■■□■■■■■■■■■■■■■□■■■■■■■■□□□□□	animal	BOV1	animal
1038	■■□■■■■■□■■■■■■□■■■■■■■■■■■■■■□■■■□■■■□□□□□	animal	BOV1	animal
1039	■■□■■■■■□■■■■■■□■■■■■■■■■■■■■■□■■■■□■■□□□□□	animal	BOV1	animal
1040	■■□■■■■■□■■■■■■□■■■■■■■■■■■■■■□■■■■■■■□□□□□	animal	BOV1	animal
1041	■■□■■■■■□■■■■■■□■■■■■■■■■■■■■■■□□□■■■■□□□□□	animal	BOV1	animal
1042	■■□■■■■■□■■■■■■□■■■■■■■■■■■■■■■□■□■■■■□□□□□	animal	BOV1	animal
1043	■■□■■■■■□■■■■■■□■■■■■■■■■■■■■■■■□■■□■■□□□□□	animal	BOV1	animal
1044	■■□■■■■■□■■■■■■□■■■■■■■■■■■■■■■■□■■■■■□□□□□	animal	BOV1	animal
1045	■■□■■■■■□■■■■■■□■■■■■■■■■■■■■■■■■□□□■■□□□□□	animal	BOV1	animal
1046	■■□■■■■■□■■■■■■□■■■■■■■■■■■■■■■■■□□■■■□□□□□	animal	BOV1	animal
1047	■■□■■■■■□■■■■■■□■■■■■■■■■■■■■■■■■□■■■■□□□□□	animal	BOV1	animal
1048	■■□■■■■■□■■■■■■□■■■■■■■■■■■■■■■■■■□■■■□□□□□	animal	BOV1	animal
1049	■■■■■■■■■■■□□■■■■■■□■■□□■■■□□□□□■□■■■■■■■■■	MANU	MANU1	MANU
1050	■■■■■■■■■■■■□■■□□□□□□□□□□□□□□□□□□□□□■■■■■■■	U	U	Unassignable
1051	■■■■■■■■■■■■■■■■■■■□□□■■■■■■■■■■□□□□■■■■■■■	T	T1	T
1052	■■■■■■■■□□■■■■□■■■□■■■■■■■■■■■■■□□□□■■■■■■■	T	S	T
1053	■■■■■■■■■■■■■■□■■■■■□□■■■■■■■■■■□□□□■■■■■■■	T	T1	T
1054	■■■■■■■■■■■■□■■■■■■■■■■■■■■□□□□□□□■□□□□■■■□■■■	H	H3	T
1055	■■■■■■■■■■■■■■□■■■■■■■■■■□□□□□□□■□□□□■■■□■□□	H	H3	T
1056	■■■■■■■■■■□□■■■■■■■■■■■■■■■■■■■■□□□□■■■□■■■	T	T2	T
1057	□□□□□□□□□□□□□□□□■■■■■■□□□■■■■■■■□□□□■■■■■■■	LAM	LAM10	T
1058	□□□□■■■■■■■■■■■■■■■■■■■■■■■■■■■■□□□□■■■□■■■	T	T2	T
1059	■■■■■■■■■■■■■■■■■■■■■■■■■□□■□□□□■■■■■■■■■■■	U	U	EAI
1060	■■■■■■■■■■■■■■■■■■■■■■■■■■□□□■■■□□□□■■■■■■■	T	T1	T
1061	■■■■□■■■■■■■■■■■■□■■■■■■■■■■■■■■□□□□■■■■■■■	X	X1	X
1062	■■■□□□□□□■■■■■■■□□□□□□□□■■■■□□□□■□■■■■■■■■■	EAI	EAI5	EAI

1063	■■■■■■■■□□■■■■■■■■■■■■■■■■■■■□■■□□□□■■■■■■■	T	S	T
1064	■■■■■■□□■■■■■■■■■■■■□□□□■■■■■■■■□□□□■■■■■■■	LAM	LAM9	LAM
1065	■■■■■■■■■■■■□□■■■■■■■■□□□■■■■■■■□□□□■■■■■■■	LAM	LAM10	T
1066	■■■■■■■■■■■■■■□■■■■■□□□□■■■■□■■■□□□□■■■■■■■	LAM	LAM6	LAM
1067	■■■■■■■□□□□□□■■■■■■■■□□□■■■■■■■■□□□□■■■□□□□	T	T1	T
1068	■■■■■■■■□□■■□■■■■■□■■■■■■■■■■■■■□□□□■■■■■■■	T	S	T
1069	■■■■■■□□■■■■■■■■■■■■■■■■■■■■■■■■□□□□■■■■■■■	T	T1	T
1070	■■■■■■■■■■■■■■■■■■■■□□□□■■■■■■■■□□□□□■■■■■■	U	U	LAM
1071	■■■■■■■■■■■■■■■□□■■■□□□□■■■■■■■■□□□□■■■■■■■	LAM	LAM9	LAM
1072	■■■■■■■■■■■■□■■□□■■■□□□□■■■■■■■■□□□□■■■■■■■	LAM	LAM5	LAM
1073	■■■■■■■■■■■□□■■■■■■■■■■■■■■■■■■■□□□□■■■■■■■	T	T1	T
1074	■■■■■■■■■■■■■■■■■■■■□□□□■■■■■■■■□□□□■■■■■□■	LAM	LAM9	LAM
1075	■■■■■■■■■■■■■■■□□□■■□□□□■■■■■■■■□□□□■■■■■■■	LAM	LAM9	LAM
1076	□□□□□□■■■■■■■■■■■■■■□□□□■■■■■■■■□□□□■■■■■■■	LAM	LAM9	LAM
1077	■■■■■■■■■■■■■■■■■■□■■■■■■■■■■■■■□□□□■■■□■■■	T	T2	T
1078	□■■■■■■■■■■■□□□□■■□■■■■■■■■■■■■■□□□□■■■□■■■	T	T2	T
1079	■□■■■■■■■■■■■■■■■■■■■□□□□■■■■■■■□□□□■■■■■■■	T	T1	T
1080	■■■■■■■■■■■■■■■■■□■■■■■■■■■■■■■■□□□□■■■□□□■	X	X1	X
1081	■■■■■■■■■■■■□■■■■□■■■■■■■■■■■■■■□□□□■■■□■■■	X	X1	X
1082	■■■■■■■■■■■■■■■■■■■■■■■■■■■■□□□□■□■■□■■□□■■	EAI	EAI5	EAI
1083	■■■■■■■■■■■■□□□□□□□□□□□□□□□□□□□□□□□■■■■□■■■	U	U	Unassignable
1084	■■□□□□□□□□□□□□□□□□□□□□□□□□□□■■■■□□□□■■■■■■■	T	T1	T
1085	■■■■■■■■■■■■■■■■■■■■■■■■■□□■■□□■□□□□■■■□■■■	H	H3	T
1086	■■■■□□□□□■■■□■■■■■■■□□□□■■■■■■■■□□□□■■■□■■■	LAM	LAM4	LAM
1087	□□■■■■■■■■■■■■■■■■■■□□□□□■■■■■■■□□□□■■■■■■■	T	T1	T
1088	■■■■■■■■■■■■■■□■■■■■■■■■■■■■■■■■□□■■■■■■■■■	MANU	MANU2	Unassignable
1089	■□□□□□□■■■■■■■■■■■■■■■□□□□□□□□□□□□■■■■■■■■■	CAS	CAS	CAS
1090	□□□■■■■■■■■■■■■■■■■■■■■■■■■□□□□■□■■■■■■■■■	EAI	EAI5	EAI
1091	■■■□□□□■■■■■■■■■■■■■■■□□□□□□□□□□□□■□■■■■■■■	CAS	CAS1	CAS
1092	■■■□□□□■□■■■■■■■■■■■■■□□□□□□□□□□□□■■■■■■■■■	CAS	CAS1	CAS
1093	■■■□□□□■■■■■■■■■■■■■□□□□□□□□□□□□□□■■■■■■■■■	CAS	CAS	CAS
1094	■■■■■■■■■■■■■■■■■■■■■■■■■■■■□■■■□□■■■■■■■■■	MANU	MANU2	Unassignable
1095	□□□□□□□□□□□□■■■■■□■■■■■■■■■■■■■■□□□□■■■■■■■	X	X1	X
1096	■□■■■■■■■■■■■■■■■■■■■■■■■■■■■■■■□□■■■■■■■■■	MANU	MANU2	Unassignable
1097	■□□□□□□□□□□■■■■■■■■■■■■■■■■■□□□□■□■■□□□■■■■	EAI	EAI3	EAI
1098	■■■■■■■■■■■■■■■■■■■■■■■■■■■■■■■■□□□□□■■□■■■	U	U	Unassignable
1099	■■■■■■■■□□□□□□□□□□□□□□□■■■■■■■■■□□□□■□□■■■■	T	T1	T
1100	□□□□■■■■■■■■■■■■■□■■■■■■■■■■■■■■□□□□■■□□□□■	X	X2	X
1101	□□□□□□□□□□□□■■■■■□■■■□□□□□□□□□□□□□□□□□□■■■	U	U	X
1102	■■□□□■■■■■■■■■■■■□■■■■■■■■■■■■■■□□□□■□□□□□■	X	X1	X
1103	■■■■■■□□□□□□□□□□□□■■■■□■■■■■■■■■□□□□■■■■■■■	T	T1	T
1104	■■■■■■■■■■■■■■■■■■■■■■□■■■■■■□■■□□□□■■■■■■■	T	T5	T
1105	■■■■■■■■■■■■■■■□■■■■■■■■■■■■■■■■□□□□■■■■■■■	T	T1	T
1106	■■■■■■■■■■■■■■■■■■■■□□□□■■■■■■■■□□□□■□□□□□□	LAM	LAM9	LAM
1107	■■■■■■■■■■■■□□□□□□□□□□□□■■■■■■■■□□□□■■■■■■■	T	T1	T
1108	□■□□□□□□□□□□□□□□■■□■■■■■□□□□□□□□□□□□□□□□□□□	animal	CAP	Unassignable
1109	□■□□□□□□□□□□□□□□■■■□□□□□□□□□□□□■■■■■■■□□□□□	animal	CAP	animal
1110	□■■■■■■■□□□□□■■■■■■■■■■■■■■■■■■■□□□□■■■■■■■	T	T1	T
1111	□■■■■■■■■■■■■■■■■■■■■■■■■■■■■■■■□□□□■■■■■□□	T	T1	T
1112	■□□□□□□■■■■■■■■■■■■■■■■■■■■■■■■■□□□□■■■■■■■	T	T1	T
1113	■□□■■■■■□□■■■■■■■■■■■■■■■■■■■■■■□□□□■■■■■■■	T	S	T
1114	■□□■■■■■■□■■■■■■■■■■■■■■■■■■■■■■□□□□■■■□■■■	T	T2	T
1115	■□■■■■■■■■■■■■■■■■■■■■■■■■■■■■□□□□□□□□□□□□□	U	U	Unassignable
1116	■■□□□□■■■■■■■■■■■■■■■■■■■■■■■■□■□□□□■■■■■■■	H	H3	H
1117	■■□■■■■□□■■■■■■■■■■■■■■■■■■■□□□■□□□□■■■■■■■	H	H4	T
1118	■■□■■■■■□■■□■■■□■■■■■■□■■■■■■■■■■■■■■■□□□□□	animal	BOV1	animal

1119	■■■□□□□□□□□□□■■■■□■■■■■■■■■■■■■■□□□□■□□■■■■	X	X3	X
1120	■■■□□□□■■■■■■■□□□□□□□□□□□□□□□□□□□□□□□■■□■■■	CAS	CAS	CAS
1121	■■■□□■■■■■■■■■■■■■■■■■■■■■■■■■■■□□□□■■□□□■■	T	T1	T
1122	■■■■■□■■■■■■■■■■■■■■■■■■■■■■■■■■□□□□■■■■■■■	T	T1	T
1123	■■■■■■□□□□□□□□□□□□■■■■■■■■■■■■■□□□□□□□■■■■■	T	T5	Unassignable
1124	■■■■■■□□□□□□□□□□■■■■■■■■■■■■■■□■□□□□■■■■■■■	H	H3	H
1125	■■■■■■□■■■■■□■■■■■■■■■■■■□□□□□□■□□□□■■■■■■■	H	H1	T
1126	■■■■■■■□■■■□■■■■■■■■■■□■■■■■■■■■■□□□□■■■■■■■	T	T5	T
1127	■■■■■■■■□□■■■■■■■■■□■■■■■■■■■■■■□□□□■■■■■■■	T	S	T
1128	■■■■■■■■□□■■■■■■■■■■■□■■□□□□■■■■□□□□■■■■■■■	T	S	T
1129	■■■■■■■■□■■■■■■■■■■■■■■■■■■■■■■■□□□□■■■■■■■	T	T1	T
1130	■■■■■■■■■□■■■■■■■■■■■■■■■■■■■■□□□□□□□□□□□□□	U	U	Unassignable
1131	■■■■■■■■■■■□□□■■■■□■■■□□■■■■■■■■□□□□■□□■■■■	T	T4	T
1132	■■■■■■■■■■■□□■■■■■■■■■■■■■■■■■□■□□□□■■■■■■■	H	H3	H
1133	■■■■■■■■■■■■□■■■■■□■■■□□■■■■■■■■□□□□■□□■■■■	T	T4	T
1134	■■■■■■■■■■■■□■■■■■■■■■■■■■■■■■■□□□■□□□□■■■□■■■	H	H4	T
1135	■■■■■■■■■■■■□■■■■■■■■■■■■■■■■■■■■□■□□□□■■■□■■■	H	H3	H
1136	■■■■■■■■■■■■■□□□□□□□□□□□■□□■■■□■□□□□■■■■■■■	H	H3	H
1137	■■■■■■■■■■■■■□□□□□□□□□□■■□■■■■■■□□□□■■■■■■■	T	T1	T
1138	■■■■■■■■■■■■■■□■■■■■■■■■■□□■■■■■□□□□■■■■■■■	T	T1	T
1139	■■■■■■■■■■■■■■■■■■□■■■■■■□□□□□□■□□□□■■■■■■■	H	H1	T
1140	■■■■■■■■■■■■■■■■■■■□□□□□■□□□□■□■□□□□■■■■■■■	H	H3	H
1141	■■■■■■■■■■■■■■■■■■■■■■□□□□■■■■■■□□□□■■■■■■■	T	T1	T
1142	■■■■■■■■■■■■■■■■■■■■■■■■■■■■■■□■□□□□□□□■■■■	U	U	H
1143	■■■■■■■■■■■■■■■■■■■■■■■■■■■■■■□■□□□□□■■■■■■	U	U	H
1144	■■■■■■■■■■■■■■■■■■■■□□□□□□□■■■■■□□□□■■■■■■■	T	T1	T
1145	■■□■■■■■■■■■■■■■■■■■□□□□■■■□□□■■□□□□■■■■■■■	LAM	LAM1	LAM
1146	■■■■□□□□■■■■□■■■■■■■□□□□■■■■□■■■□□□□■■■■■■■	T	T3	LAM
1147	■■■■■■■■■■■■■■■■■■■■□□□□□□□■□■■■□□□□■■■■■■■	T	T1	T
1148	■■■■■■■■■■■■□■■■□□□■■■■■■■■■■■■■□□□□■■■■■■■	T	T3	T
1149	■■■■■■■■■■■■■■■■■■■■■■■■■■■■■■■■■■□■■□■■■	U	U	Unassignable
1150	■■■□□□□□□□□□□■■■■□■■■■■■■■□□■■■■□□□□■■■■■■■	X	X3	X
1151	■■■□□□□□□□■■■■■■■■■■■■□□□□□□□□□□□□□■■■■■■■■	CAS	CAS	Unassignable
1152	□□□□□□□□□□□□□■■■■■■■■■■■■■■■■■■■□□□□■■■■■■□	T	T1	T
1153	■■■■■■□■□■■■□□□□□□□□□□□□□□□□□□□□□□■■■■□□■■■	AFRI	AFRI	animal
1154	■■■■■■■■■■■■■■■■■■■■□□□□■■■■■■■■□□□□■■■■□■■	LAM	LAM9	LAM
1155	■□■■■■■■■■■■■■■■■■■■■■■■■□□□□□□■□□□□■■■■■■■	H	H1	T
1156	■■□□■■■■□□■■■■■■■■■■□□□□■■■■■■■■□□□□■■■■■■■	LAM	LAM9	LAM
1157	■■■□□□□□□□□□■■■■■□■■■■■■■■■■■■■■□□□□■■■■■■□	X	X3	X
1158	■■□■□□□□□□□□□□■□■■□■■■■■■■■■■■■■■■■■■□□□□□	animal	BOV3	animal
1159	■□□□■■■■■■■■■■■■■■■■■■■■■■■■■■□■□□□□■■■■■■■	H	H3	H
1160	■■■■■■■■■■■■□■■■■■□■□□□□■■■■■■■■□□□□■■■■■■■	LAM	LAM5	LAM
1161	■■■■■■■■■■■■■□□□□□□□□□□□■■■■■■■■□□□□■□■□■■■	T	T2	T
1162	□□□□□□□□□□□□□□□□□□□□□□□□□□□□□□□□□□■□□□■■■■■	BEIJ	BEIJ	BEIJ
1163	■■□■■■■■■■■■□■■■■■■■■■■■■■■■■■■■□□□□■■■■■■■	T	T3	T
1164	■■□□■■■■■■■■■■■■■■■■■■■■■■■■■■□□□□□□□■■■■■■	U	U	Unassignable
1165	■■■■■■■■■■■■□■■■■■■■■■■□■■■■■□□□■□□□□■■■■■■■	H	H4	T
1166	■■■■■■■■■□■■■■■■■■■■■■■■■■■■■■■■□□□□■■■■■■■	T	T1	T
1167	■■■■■■■■■■■■□□□□■■■■■□■■■■■■■■■■□□□□■■■■■■■	T	T1	T
1168	□□□□□□□□□□□□□□□□□□□□□□□□□□□□□□□□□□■■■■□□■■■	BEIJ	BEIJ	BEIJ
1169	■■□■■■■■■■■■□■■■■■■□□■■■■■■■□□□□■□■■■■■■■■■	EAI	EAI2	EAI
1170	■■□■■■■■□□□■■■■■■■■□□■■■■■■■□□□□■□■■■■■■■■■	EAI	EAI2	EAI
1171	■■□■■■■■■■■■■■■■■■■□□■■■■■■■□□□□■□■□■■■■■■■	EAI	EAI2	EAI
1172	□□□□□□□□□□□□□□□□□□□□□□□□□□□■□□□□□□■■■■■■■■■	U	U	Unassignable
1173	■■■■■■□□□□□□□□□□□□■■■■■■■■■■■■■■□□□□■■■□■■■	T	T1	T
1174	■■■■■■■□□■■■■■■■■■■■■■□■■■■■□□□■□□□□■■■■■■■	H	H4	T

1175	■■■■■■■□□■■■■■■■■■■■■■■■■■■■□□□■□□□□□■■■■■■	U	U	Unassignable
1176	■■■■■■■■■■■■■□■■■■■■□□□□■■■■■■■■□□□□■■■■■■■	LAM	LAM9	LAM
1177	■■■■■■■■■■■■■■■■■■■■■■■□■■■■■■□□□□□□□□□□□□□	U	U	Unassignable
1178	■■■■■■■■■■■■■■■□□■□□□□□□□□□□□□□□□□□□■■■□■■■	U	U	Unassignable
1179	■■■■■■■■■■■■■■■■■□■■■■■■■■□□■■■■□□□□■■■■■■■	X	X1	X
1180	■■■■■□□■■■■■■■■■■■■■■■■■■■■□□□□■□■■■■■□□□□	EAI	EAI5	EAI
1181	■■□■■■■■□■■■■■■□■■■■■■□■■■■■■■■■■■■■■■□□□□□	animal	BOV1	animal
1182	■□□■■■■■■■■■■■■■■■■■■■■■■■■■□□□□■□■■■■■□■■■	EAI	EAI1	EAI
1183	■■■■■■■■■■■■■■■■■■■■■■■■■■■■□□□□■□■■□■■□■■■	EAI	EAI1	EAI
1184	□□□□□□□□□□□□□□□□■□□□□□□□□□□□□□□□□□■■■■■■■■■	U	U	Unassignable
1185	■■□■■■■■□■■□■■■□■■■■■■■■■■■■■■■■■■■■■■□□□□□	animal	BOV1	animal
1186	■■■■■■■■■■■■■■■■■■■■■■■■■■■■□□□□□□■■■■□■■■■	U	U	Unassignable
1187	■■□■■■■□□□□□□□□□□□□□□□□□□■■■□□□□□□■■■■■■■■■	U	U	animal
1188	■■■■■■■■■■■■■■■■■■■■■■■■■■■□□□□□□□■■■■■■■■■	U	U	Unassignable
1189	■■□■■■■■■■■■■■■■■■■□□■■■■■■■□□□□□□■■■■■■■■■	U	U	animal
1190	■■■■■■■■■■■■□□□□□□■■■■■■■■□■□□□□■□■■■■■■■■■	EAI	EAI5	EAI
1191	■■■■■■■■■■■■□■■■■■■■■■■■■■□■■■■■□□□□■■■□■■■	T	T2	T
1192	■■■■■■■■■■■■■■■■■■■■□■■■■■■■■■■■□□■■■■■■■■■	MANU	MANU2	Unassignable
1193	■■■■■■■■■■■■■■■■■■■■□■■■■■■■■■■■■□■■■■■■■■■	MANU	MANU1	MANU
1194	■■■■■■■■■■■■■■■■■■■■□■■■■■■■■■■■■□□□■■■■■■■	CAS	CAS	Unassignable
1195	■■■■■■■■■■■■■■□■■■■□□■■■■■■■■■■■□□■■■■■■■■■	MANU	MANU2	Unassignable
1196	■■■■■■■■■■■■■■■■■■■■■■■■■■■■■■■■□□□■■■■■■■■	U	U	Unassignable
1197	■■■□□□□■■■■■■■■■■■■■■■□□□□□□■□□□□□■■□□■■■■■	U	U	CAS
1198	■■■□□□□■■■■■□■■■■■■■■■□□□□□□□□□□□□■■□□■■■■■	CAS	CAS1	CAS
1199	■■■□□□□■■■■■■■■■■■■■■■□□□□□□□□□□□□□■□□■■■■■	CAS	CAS	CAS
1200	■■■□□□□■■■■■■■■■■■■■■■□□■■■■■■■■■■■■■■■■■■■	U	U	CAS
1201	■■□■■■■■■■■■□□■■■■□□■□□□□■■■■■■■■□□□□■■■■■■■	LAM	LAM1	LAM
1202	□□□□□□□□□□□□□□□□□□□□□□□□□■■■■■■■□□□□■■■■■■■	T	T1	T
1203	■■■□□□□■■■■■■■■■■■■■□■□□□□□□□□□□□□■■□□■■■■■	CAS	CAS1	CAS
1204	■■■■■■■■■■■■■■■□□□□□□□□□□□□□□□□□□□□■■■□■■■	U	U	Unassignable
1205	■■■■■■■■■■■■■■■■■■■■■■■■■□□□□□□■□□□□■■□■■■■	H	H1	T
1206	■□□□■■■■■■■■■■■■■■■■■■■■■■■■□□□□□□□□□□■■■■■	U	U	Unassignable
1207	■■■□■■■■■■■■■■■■■■■■■■■■■■■■■■□□□□□□■■■■■■■	U	U	Unassignable
1208	□□□□□□□□□□□□□□□□□□□□■□□□■□□□□□□■□□□□■■■■■■■	H	H1	T
1209	□□□□□□□□□□□□□□□□□□□■■■■■■■■■■■■■□□□□■■■■■■■	T	T1	T
1210	□□□□□■■■■■■■■■■■■■■■□□□□■■■■■■■■□□□□■■■■■■■	LAM	LAM9	LAM
1211	■□■■■■■■□□■■■■■■■■■■■■■■■■■■■■■■□□□□■■■■■■■	T	S	T
1212	■■□□□□□□□□□□□□□□□□□□□□□□□□□□□□■■□□□□■■■■□■■	T	T1	T
1213	■■□■□□■■■■■■□■■■■■■■□□□□■■■■■■■■□□□□■■■■■■■	LAM	LAM2	LAM
1214	■■■■■■■■■■■□□□■■■■■■■■■■■■■■■■■■□□□□■■■■■■■	T	T1	T
1215	■■□■■■■■■■■■□■■■□■■■□□□□■■■■■■■■□□□□■■■■■■■	LAM	LAM2	LAM
1216	■■□■■■■■■■■■□■■■■■■■□□□□■■■■■■□□□□□□□■■■■■■	U	U	LAM
1217	■■□■■■■■■■■■■■■■■■■□□■■■■■■■□□□□■□■■□□■■□■■	EAI	EAI2	EAI
1218	■■■□□□□□□□□□■■■■■□■■■■■■■■■■■■■□□□□□■■■■■■■	U	U	X
1219	■■■□■■■■■■■■■■■■■■■□□□■□□■■■□■■■□□□□■■■■■■■	T	T1	T
1220	■■■■□■■□□□■■■■■■■■■■□□□□■■■■■■■■□□□□■■■□□□□	LAM	LAM9	LAM
1221	■■■■□■■■■■■■■■□■■■■■■■■■■■■■■■■■□□□□■■■■■■■	T	T1	T
1222	■■■■■■□□□□□■■■■■■■■■□□□□■■■■■■■■□□□□■■■■■■■	LAM	LAM9	LAM
1223	■■■■■■■■□□□□■■■■■■■■■■■■■■■■■■■■□□□□■■■■■■■	T	T1	T
1224	■■■■■■■■□□□■■■■■■■■■□□□□■■■■■■■■□□□□■■■□□□□	LAM	LAM3	LAM
1225	■■■■■■■■□□■■■■■■■■■■■□□□□□□□□■■■□□□□■■■■■■■	T	S	T
1226	■■■■■■■■■■■□□□■■■■■■■■■■■■■■■■■■■■■■■■■■■■■	U	U	Unassignable
1227	■■■■■■■■■■■■□■■■■■■□■■□■■■■■■■■■■□□□□■■■■■■■	T	T5	T
1228	■■■■■■■■■■■■■□□□□□□□□□□■■■■■■■■■□□□□■■■□■■■	T	T2	T
1229	■■■■■■■■■■■■■□□□■■■■■■■■■■■■■■□■□□□□■■■■■■■	H	H3	H
1230	■■■■■■■■■■■■■□■■■■■■■■■■■□□□□□□■□□□□■■■■■■■	H	H1	T

1231	■■■■■■■■■■■■■■■■□□□□□■□■■■■■■■■■□□□□■■■■■■■	T	T5	T
1232	■■■■■■■■■■■■■■■■□□□□□■■■■■■■□■■■□□□□■■■□■■■	T	T2	T
1233	■■■■■■■■■■■■■■■■■■□□□■■■■■■■■■■■□□□□■■■■■■■	T	T1	T
1234	■■■■■■■■■■■■■■■■■■■■□■□□■□□■■■□■□□□□■■■■■■■	H	H3	H
1235	■■■■■■■■■■■■■■■■■■■■■■□□■□□■■■□■□□□□■■■■■■■	H	H3	H
1236	■■■■■■■■■■■■■■■■■■■■■■■□□□□□□□□□□□□□■■□□□■■	U	U	Unassignable
1237	■■■■■■■■■■■■■■■■■■■■■■■□□■■■■□■■□□□□■■■■■■■	T	T1	T
1238	■■■■■■■■■■■■■■■■■■■■■■■■■■□■■■□■□□□□■■■■■■■	H	H3	H
1239	■■■■■■■■■■■■■■■■■■■■■■■■■■■□□■■■□□□□□□□□■■■	U	U	Unassignable
1240	■■■■■■■■■■■■■■□□□□□□□□□□□□■■■■■■□□□□■■■■■■■	T	T1	T
1241	■■■■■■■■■■■■■■■■■■■■□□□□■■■■■■□□□□□□■■■■■■■	U	U	LAM
1242	■■■■■■■■□□■■■■■■■■■■■■■■■■■■□□□■□□□□■■■■■■■	H	H4	T
1243	■■■■■■■■■□■■■■■■■■■■■■■■■■■■■■□■□□□□■■■■■■■	H	H3	H
1244	■■■■■■■■□□■■■■■■■■■■■■■■■■■■■■□■□□□□■■■□■■■	T	S	H
1245	■■■■■■■■□□■■■■■■■■■■■■■■■■■■□□□□□□□□□□□■■■■	U	U	Unassignable
1246	■■■■■■■■■■■■■■■■■□■■■■■■■■■■■■□■□□□□■■■■■■■	H	H3	Unassignable
1247	■■■■■■■■■■■■■■■■■■■■□□□□■■■■■■■■□□■■■■■■■■■	MANU	MANU2	LAM
1248	■■■■■□□□□□□□□□□□■■■■■■■■■■■■■■■■□□□□■■■■■■■	T	T1	T
1249	□□□□□□□■■■■■■■■■■■■■□□□□■■■■■■■■□□□□■■■■■■■	LAM	LAM9	LAM
1250	■■■■■■■■■■■■■■■■■■■■■□■■■■■■■■■□□□□□■■■■■■■	U	U	Unassignable
1251	■■■■■■■■■■■■■■□■■■■■■■■■■■■■□□□□■□■■■■■□■■■	EAI	EAI1	EAI
1252	■■■■■■■■■■■■■■■■■■□□■■■■■■■■■■■■□□□□■■■■■■■	T	T1	T
1253	■■□■■■■■□□■■■■■■■■■■■□■■■■■■■■■■□□□□■■■■■■■	T	S	T
1254	■■■■■■■■■■■■■■■■■□■■■■■■■■■□□□■■□□□□■■■■■■■	X	X1	X
1255	□□■■■■■■■■■■■■■■■■■■□■□■■■■■■■■■□□□□■■■■■■■	T	T5	T
1256	■■■■■■□□□□□■■■■■■■■■■■■■■□□□□□□■□□□□■■■■■■■	H	H1	T
1257	■■■■■■■■■■■■□■■■■□□□□□□□□□□□□□□□□□□□□□□□□□□	U	U	Unassignable
1258	■■■□□□■■■■■■■■■■■■□■■■□□■■■■■■■■□□□□■□□■■■■	T	T4	T
1259	■■■■■■■■■■■■■□□□□□□□□□□□□□□□□■■■□□□□■■■■■■■	T	T1	T
1260	□□□□□□□□□□□□□□□□□□□□□□□□■■■■■□□■□□□□■■■■■■■	H	H3	T
1261	■■■■■■■■■■■■■■■■■■■□□□□□■□□■■■■■□□□□■■■□■■■	LAM	LAM7	T
1262	□□□□□□□□□□□□□□□□□□□□□□□□■■■□□□□□□□□□■■■■■■■	U	U	Unassignable
1263	■□□■■■■■■■■■■■■■■■□■■■■■■■■■□□□□■□□□■■■■■■■	CAS	CAS	EAI
1264	■■■□□□□■■■■■■■■■■■■■■■□□□□□□□□□□□□□□□□□□□□□	CAS	CAS	CAS
1265	■■■■■■■■□■■■■■■■■■■■■■■■■■■■■■■□□□□■■■□■■■	T	T2	T
1266	■■■□□□□□□■■■■■■■■■■■■■□□□□□□□□□□□□■■■■■■■■■	CAS	CAS	CAS
1267	■■■■■■■□□□□□□■■■■■■■■■■■■■■■■■■■■□□□□□■■□□□□	U	U	Unassignable
1268	■■■■■■■■■■■■■■□□□□□□□■□■■■■■■■■■□□□□■■■■■■■	T	T5	T
1269	■□■■■■■□□■■■■■■■■■■■■■■■■■■■□□□■□□□□■■■■■■■	H	H4	T
1270	■■■■□■■■■■■■■■■■■□■■■■■■■■■■■■■■□□□□■■□□□□■	X	X2	X
1271	■■■■■■■■□□■■■■■■■■■■□■■■■■■■■■■■□□□□■■■■■■■	T	S	T
1272	■□■■■■■■■■■■□■■■■■■■■■■■■■■■■■■■□□□□■□■■■■■	T	T3	T
1273	■■■□□□□□□□□□■■■■■□■■■■■■■■■■■■■■□□□□■■■□■■■	X	X3	X
1274	■■■■■■■■■■■■■■■■■■■■■■■□□□□□□□□□□□□□□□□■■■■	U	U	Unassignable
1275	■■■■■■■■■■■■■■■■■■■■■■■■■□□□□□□□□□□□□■■■■■■	U	U	Unassignable
1276	■■■■■■■■■□□□□□■■■■■■■■■■■■■■□□□■□□□□■■■■■■■	H	H4	T
1277	■■■■■■■■■■■■■■■■■■□■□□□□■■■■■■■■□□□□■■■■■■■	LAM	LAM9	LAM
1278	□□□□■■■■■■■□■■■■■■■■■■■■■■■■■■■■□□□□■■■■■■■	T	T1	T
1279	■■■□■■■■■■■■■■■■■■■■■■□■■■■■■■■■□□□□■■■■■■■	T	T5	T
1280	□□□□□□□□□□□□□□□□□□□□□□□□■■■■■■■■□□□□■■■■■■□	T	T1	T
1281	■■■■■■■■■■□■□■■■■■■■■■■■■■■■□□□■□□□□■■■■■■■	H	H4	T
1282	■■■■■■■■■■■■■■■■■■■□□□□□□□□□□□□■□□□□■■■■■■■	H	H3	T
1283	■■■■■■■■■■■■■■■■■■■■■■■■■■■□□□□■□□□□■■■■■■■	H	H3	T
1284	■■■■□□□□■■■■□□□□■■■■■■■■■■■■■■■■□□□□■■■■■■■	T	T1	T
1285	■□■■■■■■■■■■■■■■■□■■■■□■■□■■□□□□■□■□■■■□■■■	EAI	EAI6	EAI
1286	■□□■■■■□□□□■■■■■■■■■■■■■■■■■■□□□■□■■■■■■■■■	MANU	MANU1	MANU

1287	■□□□□□□□□□□□□■■■■■■■■■■■■■■■■□□□■□■■■■■■■■■	MANU	MANU1	MANU
1288	■■■■■■■■■■□□■■■■■■■■□□□□■■■■■■■■□□■■■■■■■■■	MANU	MANU2	LAM
1289	■■■■■■■■■■■■■■■■■■■■■□□■■■■■■■■■□□■■■■■■■■■	MANU	MANU2	Unassignable
1290	■■■■■■■■■■■■■■■■■■■■□□□□■■■■■■□■□□■■■■■■■■■	MANU	MANU2	Unassignable
1291	■■■■■■■■■■■■□■■■■■■■■■■■■■■■□□□■□□■■■■■■■■■	MANU	MANU2	Unassignable
1292	■■□■■■■□□■■■■■■■■■□■■■■■■■■■□□□■□□□□■■■■■■■	H	H4	T
1293	■■□■■■■■□□□■■■■■■■■■□□□□■■■■■■■■□□□□■■■■■■■	LAM	LAM3	LAM
1294	■■■■■■■□□□□■■■■■■■■□□□□□■■■■■■■■□□□□■■■■■■■	T	T1	T
1295	■■■■■■■■□□□■■■□□■■■■□□□□■■■■■■■■□□□□■■■■■■■	LAM	LAM3	LAM
1296	■■■■■■■■□□■■■■■□□■■■■■■■■■■■■■■■□□□□■■■■■■■	T	S	T
1297	■■□■■□■□□□□□■■■□■■□□□■■■■■■■■■■■■■■■■■□□□□□	animal	BOV2	animal
1298	■■□■□□□■□□■□□□■■■■■■■■■■■■■■■■■■■■■■■■□□□□□	animal	BOV	animal
1299	■□□■□□□□□□□□□□□□■■■■■■■■■■■■■■■■■■■■■■□□□□□	animal	BOV	animal
1300	□□□□□□□□■■■■■■■■■□■■■■■■■■■■■■■■□□□□■■□□□□■	X	X2	X
1301	■□■■■■■■■■■■■■■■■■■■■■■■■■■■■■■■□□□□■■□□□□■	T	T1	T
1302	■□■■■■■■■■■■■■■■■■■■■■■■■■■■■■■■□□□□■■■□■■■	T	T2	T
1303	■■□□■□■□□□□□■■■□■■■■■■■■■■■■■■■■■■■■■■□□□□□	animal	BOV	animal
1304	■■□■□□□□□□□□□□■□■■■■■□■■■■■■■■■■■■■■■■□□□□□	animal	BOV3	animal
1305	□□□■■□■□□□□□■■□□■■■■■■■■■■■■■■■■■■■■■■□□□□□	animal	BOV	AFRI
1306	□■□■■□■□□□□□■■□□■■■■■■■■■■■■■■■■■■■■■■□□□□□	animal	BOV	animal
1307	□□□■■□■□□□□□■■□□□■■■■■■■■■■■■■■■■■■■■■□□□□□	animal	BOV	AFRI
1308	■■□■■□■□□□□□■■□□■■■■■■■■■■■■■■□□□□□□■■□□□□□	animal	BOV	animal
1309	□□□■■□■□□□■□■■□□■■■■■■■■■■■■■■■■■■■■■■□□□□□	animal	BOV	animal
1310	■□□■■□■□□□■□□■□□■■■■■■■■■■■■■■■■■■□□■■□□□□□	animal	BOV	animal
1311	□□□□□□□□□□□□□□□□□□□□□□□□□□□□□□□□□□■■■■■□□□□	U	U	BEIJ
1312	■■■□□□□■■■■■■■■■■■■■■■□□□□□□□□□□□□■■□□■□■■■	CAS	CAS1	CAS
1313	■■■■■■■■■■■■■■■■■■■■■■■■■□■■□□□□■□■■■■■□■■■	EAI	EAI1	EAI
1314	■■■□□□□■■□■■■■■■■■■■■■□□□□□□□□□□□□■■□□■■■■■	CAS	CAS1	CAS
1315	■■■□□□□□□□□□■■□■■□■■■■■■■■■■■■■■□□□□■■□■■■■	X	X3	X
1316	■■■□□■■■■■■■■■■■■■■■■■■■■■■■□□□□■□■■■■■□■■■	EAI	EAI1	EAI
1317	■□■■■■■■■■■■■■□■■■■■■■■■■■■■■■■■□□□□■■□■■■■	T	T1	T
1318	■□■■■■■■■■■■■■□■■■■■■■■■■■■■■■■■□□□□■■■■■■■	T	T1	T
1319	■□■□□□□□□□■■■■□■■■■■■■□□□□□□□□□□□□■■■■■■■■■	CAS	CAS2	CAS
1320	■■■□□□□□□□□■■■■■■■■■■■□□□□□□□□□□□□■■■■■■■■■	CAS	CAS	CAS
1321	■■□■■■■■■■■■■■■■■■■■□□□□■■■■■■■■□□□□■■■□■■■	LAM	LAM1	LAM
1322	■■■■■■■■■■■■□□□□■■□□□■■■■■■■■■■■□□□□■■■■■■■	T	T1	T
1323	■□■□□□□■■■■■■■□■■■■■■■□□□□□□□□□□□□■■■■□■■■■	CAS	CAS1	CAS
1324	■■■■■■■■■■■■■■□□□□□□□■□□■■■■■■■■□□□□■■■■■■■	T	T1	T
1325	■■■■■■■■■■■■■■□□□■□□□■□■□□□□□□□□□□□□■■■■■■■	U	U	Unassignable
1326	■■■■■■■■■□□□□■■■■■■■■■■■■■■■□□□□■□■■■■■□□□□	EAI	EAI5	EAI
1327	■■■□□□□■■■■□□■■■■■■■■■□□□□□□□□□□□□■■■■■■■■■	CAS	CAS1	CAS
1328	■■■■■■■■■■■■■■■■■■□□□□■■■□□□□□□■□□□□■■■■■■■	H	H1	T
1329	■■■■■■■■■■■■■■■■■□■■■■■■■■■■□■■■□□□□■■■■■■■	X	X1	X
1330	□■■■■■■■■■■■■■■■■■■■■■□■■■■■■■■■□□□□■■■■■■■	T	T5	T
1331	■■■■■■■■□□□■■■■■□■■□□□□□■■■■■■■■□□□□■■■■■■■	T	T1	T
1332	■■□■■■■■■■■■■■■■■■■■■■■■■■■■■■■■□□□□■■■□■■■	T	T2	T
1333	■■■■■■■■□□■■■■■□□□□□□□□□□□■■■■■■□□□□■■■■■■■	T	S	T
1334	■■■■■■■■□□■■■■■□□□□□□□□□□□■■■■■■□□□□■■■□■■■	T	S	T
1335	■■■■■■■■□□□■■■■■■■■■□□□□■■■■■■■■□□□□■□□□□□□	LAM	LAM3	LAM
1336	■■□■■■■■□□■■■■■■■■■■■□□■■■■■■■■■□□□□■□□■■■■	T	S	T
1337	■■■■■■■■■■■■□□□□□□■■□□□□■■■■■■■■□□□□■■■■■■■	LAM	LAM9	LAM
1338	■■■■■■■■■■■■■■■■■■■■■■■■■■■■■■■■■■■■■■□■■■■	U	U	Unassignable
1339	■■□■■■■■■■■■□■■■■■■■■■■■■■■■■■■■□□□□■■■□■■■	T	T2	T
1340	■■■■■■■■■■■■■■■■□■■■■■□■■■■■□□□□■□■■□■■■■■■	EAI	EAI6	EAI
1341	■■■■■■■■■■■■■■■■■□■■■■□■■■■■■■■■□□□□■■□□□□■	X	X2	X
1342	■□□■■■■■■■■■■■■■■■□□■■■■■■■■□□□□■□■■□□□■■■■	EAI	EAI3	EAI

1343	■■■□□□□■■■■■□■■■■■■■■■□□□□□□□□□□□□■■■■■■■■■	CAS	CAS1	CAS
1344	■■■□□□□■■■■■■■■■■■■□□■□□□□□□□□□□□□■■■■■■■■■	CAS	CAS1	CAS
1345	■■■□□□□■■■■■■■■■■■■■■□□□□□□□□□□□□□□□□■■■■■■	CAS	CAS	CAS
1346	■■■□□□□■■■■■■■■■■■■■□■□□□□□□□□□□□□■■■■■■■■□	CAS	CAS1	CAS
1347	■■■■■■■■■■■■□□□□□□■■■■■■■■■■■■■■□□□□■■■■■■■	T	T1	T
1348	■■■■■■■□□□□□□■■■■■■■■■■■■■■■■■■■□□□□■■■□□□■	T	T1	T
1349	□□□■□■■■■■■■■■■■■■■■■■■■■■■■■■■■□□□□■■■■■■■	T	T1	T
1350	■■■■■■■■■□□□□□■■■■■■■■■■■■■■■■□□□□□□□□□□□□□	U	U	Unassignable
1351	■■■■■■■■□□■■■■■■■■■■□■■■■■■■■■□□□□□□■■■■■■■	U	U	Unassignable
1352	■■■■■■■■■■■□■■■■■■■■■■■■□□□□□□□□□□□□□□□□□□□	U	U	Unassignable
1353	■■■■■■■■■■■■■■■■■■■■■■■■■□□□□□□□□□□□□□□□□□□	U	U	Unassignable
1354	■□■■■■■■□□□■■■■■■■■■□□□□■■■■■■■■□□□□■■■■■■■	LAM	LAM3	LAM
1355	■■■■■■■■■■■■■■■■■■■□□□□□■■■■□■■■□□□□■■■□■■■	T	T2	T
1356	■■■■■■■■□□■■■■■■■■■■■■■■■■■■■■■■□□□□■■■■□■■	T	S	T
1357	■■■□□□□■■■■■■■■■■■■□□□□□□□□□□□□□□□■■□□□□■■■	CAS	CAS	CAS
1358	■■■■■■■■■■□□□□■■■■■■■■■□□□□□□□■■□□□□■■■■■■■	T	T1	T
1359	■□□■■■■■■■■■■■■■□□□■■■■■■□□□□□□■□□□□■■■■■■■	H	H1	T
1360	■□■■■■■■■■■■■■■■■■■■■■■■■■■■■■□■□□□□■■□□■■■	H	H3	H
1361	■■■■□■■■■■■■□■■■■■■■■■■■■■■■■■■■□□□□■■■□■■■	T	T2	T
1362	■■□■■■■■■■■■■■■■■■■■■□□□□□□□□□□□□□□□□■■■■■■	U	U	animal
1363	■■■■■■■■■■■■■■■■■■■■■■■■■■■■□□□□□□□□□□■■■■■	U	U	Unassignable
1364	□□□□□□□□□□□□□□□□□□□□□□□□□□□□□□□□□□■■■□□■■■■	BEIJ	BEIJ	BEIJ
1365	□□□□□□□□□□□□□□□□□□□□□□□□■■■■□□□□■□■■■■■■■■■	EAI	EAI5	EAI
1366	□□□□□□□□□□□□■■■■■□■■■■■■■■■■■■■■□□□□■■□■■■■	X	X1	X
1367	□■■■■■■■■■■■□■■■■■■■□□□□■■■■■■■■□□□□■■■■■■■	LAM	LAM5	LAM
1368	■■■□□□□□■■■■■■■■■■■■■■□□■■■■■■■■■□□□□■■■■■■	U	U	Unassignable
1369	■□□■■■■■■■■■■■■■■■■■■■■■■■■■□□□□■□■■■■□■■■■	EAI	EAI5	EAI
1370	■■■■□■■■■■■■■■■■■■■■■■□■■■■■■■■■□□□□■■■■■■■	T	T5	T
1371	■■■■■□■■■■■■■■■■■■■■□□□□■■□■■■■■□□□□■■■■■■■	LAM	LAM8	LAM
1372	■■■■■■■■■■□■■■■■■■■■■■■■■■■■□□□□■□■■■■■■■■■	EAI	EAI5	EAI
1373	■■■■■■■■■■■□■■■■■■■■■■■■■■■■□□□□■□■■■■■□■■■	EAI	EAI1	EAI
1374	■■■■■■■■■■■■■■■■■■■■■□□□□□□□□□□□□□■■■■■■■■■	U	U	Unassignable
1375	■■■■■■■■■■■■■■■■■■■■■■□■□■■■□□□□■□■■■■■■■■■	EAI	EAI6	EAI
1376	■■■■■■■■■■■■■■■■■■■■■■■■■□■■□□□□■□■■□■■■■■■	EAI	EAI5	EAI
1377	■■■■■■■■■■■■■■■■■■■■■■■■■■■■□□□□■□■□■■□■■■■	EAI	EAI5	EAI
1378	■■■■■■■■■■■■■■■■■■■■■■■■■■■■■■■■■□□□■■■■■■■	CAS	CAS	Unassignable
1379	■■■■■■□■■■■■■■■■■■■■■■■■■■■■■■■■■□□□■■■■■■■	CAS	CAS	Unassignable
1380	■■■■■■■□□■■■■■■■■■■■■■■■■■■■□□□□■□□□■■■■■■■	CAS	CAS	EAI
1381	■■■■■■■□□■■■■■■■■■■■■■■■■□■■□□□□■□□□■■■■■■■	CAS	CAS	EAI
1382	■■■■■■■■■■■■■■■■■■■■■■■■■■□■■■□■□□□□■■□■■■■	H	H3	H
1383	■■■■■■□■■■■■■■■■■■■■■■■■■■■■■■■□□□□■■■□■■■	T	T2	T
1384	□□□□□□□□□□□□□□□□□□□■□■■■■■■■□□□■□□□□■■■■■■■	H	H4	T
1385	■□■□■■■■■■■■■■□■■■■■■■■■■■□■■■■■□□□□■■□□■■■	T	T1	T
1386	■■■■■■■■■■■■■■■■■■■■■■■■■■■■□□□□□□■■■■■□□□■	U	U	Unassignable
1387	■□□□□□■■■■■■■■□□□□□□□□□□■■■■■■■■□□□□■■■■■■■	T	T5	T
1388	■■■■■■■■■■■■■■■■■■■■■■□□□■■■□□□□■□■■□■■□■■■	EAI	EAI1	EAI
1389	□□■■■■■■■■■■■■■■■■■■■■■■■■■■□□□□■□■■■■■□■■■	EAI	EAI1	EAI
1390	■■■■■■■■■■■■■■■■■■■■■■□□■■■■□□□□■□■■□■■■■■■	EAI	EAI5	EAI
1391	■■■■■■■■■■■■■■■■■■■■■□□□□□□□□□□□□□■■□■■■■■■	U	U	Unassignable
1392	■■■■■■■■■■■■■■■■■■■■■■■■■■■■□□□□■□■■■□□□■■■	EAI	EAI5	EAI
1393	■■■■■■■■■■■■■■■■■■■■■■■■■■■■□□□□□■□■■■■□□□□	U	U	Unassignable
1394	■■■■■■■■■■■■■■■■■□■■■■■■■■■■■■■■□□□□■■□■■■■	X	X1	X
1395	■□□■■■■□□□■■■■■■■■■■■■■□■■■■□□□□■□■■■■■■■■■	EAI	EAI5	EAI
1396	■■■■■■■■■■■■■■■■■■■■■■□■□□□□□□□□□□■□□■■■■■■	U	U	Unassignable
1397	■■■■■■■■■■■■■■■■■■■■■■■□□□■■□□□□■□■■■■■□□□□	EAI	EAI5	EAI
1398	■□■■■■■■■■■■■■■■■■■■■■□□□□□□□□□□□□■■■■■■■■■	U	U	CAS

1399	□□□■■■■■■■■■■■■■■■■■■■■■■■■■□□□□■□■■■■■□□□□	EAI	EAI5	EAI
1400	■■■□□□□□□□□□□□□□□□□□□□□□■■■■□□□□■□■■□■■■■■■	EAI	EAI5	EAI
1401	■■■□□□□■■■■■■■■■■■■■■■□□□□□□□□□□□□■■■■□■■■■	CAS	CAS1	CAS
1402	■□■■■■■■■■■■■■■□□□□□□■□■□□□□□□□□□□□□□□□□□□□	U	U	Unassignable
1403	■■■■■■■■■■■■■■■■■■■■■■□■■■■□□□□□□□■■□■■■■■■	U	U	Unassignable
1404	■■■■■■■■■■■■■■■■■■■■■■■□■■■■□□□□■□■■■■■□■■■	EAI	EAI1	EAI
1405	■■■□□□□■■■■■■□□□■■■■■■□□□□□□□□□□□□■■■■■■■■■	CAS	CAS1	CAS
1406	■■■■■■■■■■■■■■■■■■■■■■□■■■■■□□□□■□■■□□■■■■■	EAI	EAI6	EAI
1407	■■■■■■■□■□■■■■■■■■■■■■■■■■■■□□□□■□■■■■■■■■■	EAI	EAI5	EAI
1408	□□□□□□□□□□□□□□□□□□□□□□□□■■■■□□□□■□■■□■■■■■■	EAI	EAI5	EAI
1409	■■■■■■■■■■■■■■■■■■■■■■□■■■■■□□□□■□■■□■□■■■■	EAI	EAI6	EAI
1410	■■■■■■■■■■■■■■■■■■■■■□□□□□□□□□□□□□□□□□□□□□□	U	U	Unassignable
1411	■■■■■■■■■■□□■■■■■■■■■■□■■■■■□□□□■□■■□■■■■■■	EAI	EAI6	EAI
1412	■■■■■■■■■□■■■■■■■■■■■■□■■■■■□□□□■□■■□■□■■■■	EAI	EAI6	EAI
1413	□□■■■■■■■■■■■□□□■■■■■■■■■■■■□□□□■□■■■■■□■■■	EAI	EAI1	EAI
1414	■■■■■■■■■■■■■□■■■■■■■■□■■■■■□□□□■□■■□■■■■■■	EAI	EAI6	EAI
1415	□□□■■■■■■■■■■□□□■■■■■■■■■■■■□□□□■□■■■■■□■■■	EAI	EAI1	EAI
1416	■■■■■■■■■■■■■□□■■■■■■■□■■■■■□□□□■□■■■■■■■■■	EAI	EAI6	EAI
1417	■■■■■■■■■■■■■■■■■■■■■■□■■■■■□□□□■□■■□□□■■■■	EAI	EAI6	EAI
1418	■□■■■■■■■■■■■■■■■■■■■■□■□□□□□□□□□□■■■■■■■■■	U	U	Unassignable
1419	■■■□□■■■■■■■■■■■■■■■■□□□□□□□□□□□□□■■□■■■■■■	U	U	Unassignable
1420	■■■■■■■■■■■■■■■■■■■■■■□■■■■■□□□□■□□□□□□■■■■	U	U	EAI
1421	■■■■■■■■■■■■■■■■■■■■■■□■■■■■□□□□□□■■□■■■■■■	U	U	Unassignable
1422	■■■□□□□■■■■■■■■■■■□□□□□□□□□□□□□□□□□□■■■■■■■	CAS	CAS	CAS
1423	■□□■■■■■■■■■■■■■□□■■■■■■■■■■□□□□■□■■■■■■■■■	EAI	EAI5	EAI
1424	■■■■■■■■■■■■■■■■■■□□□□□■□□□□□□□□□□■■□■■■■■■	EAI	EAI7	Unassignable
1425	■■■■■■■■■■■■■■■■■■■■■■□■■■■■□□□□■□■■□■■□■■■	EAI	EAI6	EAI
1426	■■■■■■■■■■■■□■■■■■■■■■■■■■■■■■■□□□□■■■□□■■	T	T3	T
1427	■□□■□■■■■□□□□□□□□■■■■■■■■■■■□□□□■□■■■■■■■■■	EAI	EAI5	EAI
1428	■■■■■■■□□■■■□□□■■■■■■■■■■■■■□□□□■□■■■■■□■■■	EAI	EAI1	EAI
1429	■■■□□□□■■■■■■■■■■■■■■■□■□□□□□□□□□□■■■■■■■■■	U	U	CAS
1430	■■■■□■■■■■■■■■■■■■■■■□□□□□□□□□□□□□■■□■■■■■■	U	U	Unassignable
1431	□□■■■■■□□□□□□□□□□□□□□□□□■■■■□□□□■□■■■■■□■■■	EAI	EAI1	EAI
1432	■■■■■■■■■■■■■□□□■■□■■□□□□□□□□□□□□□□□□□□□□□□	U	U	Unassignable
1433	■□■■■■■■■■■■□■■■■■■■■■□■■■■■□□□□■□■■■■■■■■■	EAI	EAI6	EAI
1434	■■■■■■□■■■■■■■■■■■■■■■□□□□□□□□□□□□□□□□■■■■■	U	U	Unassignable
1435	■■■■■■■□□■■■■■■■■■■■■■■■■■■■□□□□■□■■■■■□■■■	EAI	EAI1	EAI
1436	■□■■■■■■■■■■■■■■■■■■■■□■■■■■□□□□□□■■■■■■■■■	U	U	Unassignable
1437	■■■□□□□■■■■■□■■■■■■■■■□□□□□□□□□□□□■■■■■□■■■	CAS	CAS1	CAS
1438	■■■■■■■■■■■■■■■■■□■■■■□■■■■■□□□□■□■■□■■■■■■	EAI	EAI6	EAI
1439	■■□■■■■□□□■■■■■□■■■■□■■■■■■■■■■■■■■■■■□□□□□	animal	BOV	animal
1440	■■□■■■■■□■■■■■■■■■■■□■■■■■■■■■■■■■■■■■□□□□□	animal	BOV	animal
1441	□□□■■■■■□■■■■■■■■■■■■■■■■■■■■■■■■■■■■■□□□□□	animal	BOV	animal
1442	■■□□□■■□□□□□■■■■■■■■■■■■■■■■■■■■■■■■■■□□□□□	animal	BOV	AFRI
1443	■■□□□■■■□■■■■■■■■■■■■■■■■■■■■■■■■■■■■■□□□□□	animal	BOV	animal
1444	■■■■■■■■■■□□□□□□□□□□□□□□□□□□□□■■□□□□■■■■■■■	T	T1	T
1445	■□□□□□■■■■■■■□■■■■■■□□□□■■■■■■■■□□□□■■■■■■■	LAM	LAM9	LAM
1446	■■■■■■■■■■■■■□■■■■■■□■■■■■■■□□□■□□□□■■■■■■■	H	H4	T
1447	■■■■■■■■■■■□□■■■■■■■■■■■■■■■□□□■□□□□■■■■■■■	H	H4	T
1448	■■■■■■■■■■■□□■■■■■■■■■■■■■■■□□□■□□□□■■■□■■■	H	H4	T
1449	■■■■■■■■■■■■■■■■■■■■□■■■■■■■□□□■□□□■■■■■■■■	U	U	Unassignable
1450	■■■■■■■■■■■■■■■■■■■■□■■■□□□□□□□□□□□□■■■■■■■	U	U	Unassignable
1451	■■■■■■■■■■■■■■□□□□□□□□□□□□□□□□□□□□□□□□□□□□□	U	U	Unassignable
1452	■■■■■■■□□■■■■■■■■■■■□■■■■■■■□□□■□□□□■■■■■■■	H	H4	T
1453	■■■■■■■■□□□■■■■■■■■■□□■■■■■■■■■■□□■■■■■■■■■	MANU	MANU2	Unassignable
1454	■■■■■■■□□□□□□□□□□□■■□■■■■■■■■■■■□□□□□■■■■■■	U	U	Unassignable

1455	■■■■■■■□□□■■■■■■■■■■□■■■■■■■□□□■□□□□■■■■■■■	H	H4	T
1456	■■■■■■■■■■■□□■■■■■■■□■■■■■■■□□□■□□□□■■■■■■■	H	H4	T
1457	■■■■■■■■■■■■■□■■■■■■■■■■■■■■□□□■□□□□■■■■■■■	H	H4	T
1458	■■■■■■□■■■■■■□■■■■■■■■■■■■■■■□□□□□□□□■■■■■■■	U	U	Unassignable
1459	■■■■■■■■■■■■■■■■■□■■■■■■■■■■■■■■□□□□■■■□□□□	X	X1	X
1460	■■■■■■■■■■■■■■■■■■□■■■■■■■■■■■■■■□□■■■■■■■■■	MANU	MANU2	Unassignable
1461	■■■■■■□■■■■■□■■■■■■■□■■■■■■■□□□■□□□□■■■■■■■	H	H4	T
1462	■■■■■■■■■■■■■■■■■■■■■■■■□□□□□□□□□□■■■■■■■■■	U	U	Unassignable
1463	■■□■■■□□□□□□□□□□□□■■■■■■■■■■■■■■□□□□■■■□■■■	T	T1	T
1464	■■■■■■■■■□■■■■■■■■■■■■■■■■■■■■■■■■□□□□□□□□□□□□■■■■■■■	U	U	Unassignable
1465	■■■■■■■□□□□□■■■■■■■■■■■■□□□□□□□□□□□■□□□■■■■	AFRI	AFRI3	AFRI
1466	■■■■□■■■■■■■■■■■■■■■□□□□■■□□□□■■□□□□■■■■■■■	LAM	LAM11	LAM
1467	■□□■■■■■■■■■■■■■■■■■■■■■■■■■□□□□■□□□■■■■■■■	CAS	CAS	EAI
1468	□□□■■■■■■■■■■■■■■■■■□□□□■■□□□□■■□□□□■■□■■■■	LAM	LAM11	LAM
1469	□□□□□■■■■■■■■■■■■□■■□□□□■■■■■■■■□□□□■■■■■■■	X	X1	Unassignable
1470	■■■■■■■■■■■■■■■■■□■■□□□□■■■■■■■■□□□□■■■■■■■	X	X1	Unassignable
1471	■■■■■■■■■■■■□■■■■■■■□□□□■■□□□□■■□□□□■■■■■■■	LAM	LAM11	LAM
1472	■□□■■■■■■■■□■■■■■■■■■■■■■■■□□□□■□□□■■■■■■■	CAS	CAS	EAI
1473	■■■□□□□□□■■■■■■■■■■■■■□□■■□□□□□□■□■■■■■■■■■	MANU	MANU1	MANU
1474	■■□■■■■■■■■■■■■■■■■■□□■□■■■■■■■■□□□□■■■■■■■	T	T1	T
1475	■■■■■■■■■□■■■□■■■■■■■■■■■■■■■■■■□□□□■■■■■■■	T	T1	T
1476	■■■□■■■■□□□■■■■■■■■■■□□□□■■■■■■□□□□□□□□■■■■■	U	U	LAM
1477	■■□■■■■■■■■■■■■■■■■□□■■□□■■■□□□□■□■■■■■□□□■	EAI	EAI2	EAI
1478	■■□■■■■□■■■■■■■■■■■□□■■■■■■■□□□□■□■■■■■■■■■	EAI	EAI2	EAI
1479	■■□■■■■□□■■■■■■■■■■□□■■■□□□□□□□□□□□■■■■■■■■	U	U	animal
1480	■■■■■■■□□■■■■■■■■□□□□□□□□□□■□□□■□□□□■■■■■■■	H	H4	T
1481	■■■■■■■□□■■■■■■■■■■■■■■■■■■□□□■□□■■■■■■■■■	MANU	MANU2	Unassignable
1482	■■■■■■■□■■■■■□□□□□□□□□□□□■■■■■■■□□■■■■■■■■■	MANU	MANU2	Unassignable
1483	■■■■■■■■■■■■□□■■■■□■■■■■■■■■■■■■■□□□□■■■■■■■	T	T1	T
1484	■■■■■■■■■■■■■■■■■■■■■■■■■□□□□□□■□□■■■■■■■■■	MANU	MANU2	Unassignable
1485	■■■■■■■■■■■■■□□□□□□□□□□□■■■■■■■■□□■■■■■■■■■	MANU	MANU2	Unassignable
1486	□■■□□□□□□□□□□■■■■□■■■■■■■■■■■■■■□□□□■■■■■■■	X	X3	X
1487	□□□□□□□□□□□□□□□□□□□□□□□□■■■■■■■■■■■■■■■□■■■	U	U	Unassignable
1488	■□□□□□□□■■■■■■■■■■■■■■■■■■■■□□□□■□□□□□□■■■■	U	U	EAI
1489	■■□□■■■■■■■■■■■■■■■□□■■■■■■■□□□□■□■■■■■■□■□	EAI	EAI5	EAI
1490	■■□■■■■■■■■■■■□■■■■□□■■■■■■■□□□□■□■■■■■■■■■	EAI	EAI2	EAI
1491	■■■■□□□□□□□□□□□□□□□□□□□□■■■■■■■■□□□□■■■□■■■	T	T2	T
1492	■■■■■■■■■■■■□□□□□□□□□□□□□□□□□□□□□□□■□□■□■■■	U	U	Unassignable
1493	■■■■■■■■■■■■■■■■■■■■■□□□□□■■□□□□■□■■■■■□□□□	EAI	EAI5	EAI
1494	■■■■■■■■■■■■■■■■■■■■□■■■■■□■■■■■□□□□■■■■■■■	T	T1	T
1495	■■■■■■■■■■■■■■■■■■■■■□■■■■■■□□□□■□■■■■■■■■■	EAI	EAI5	EAI
1496	■■■■■■■■■■■■■■■■■■■■■■□■■■□■□□□□■□■■■■■■■■■	EAI	EAI6	EAI
1497	■■■■■■■■■■■■■■■■■■■■■■■■■□■■□□□□■□■■■■□■■■■	EAI	EAI5	EAI
1498	■■■■■■■■■■■■■■■■■■■■■■■■■■□□□□□□□□□□□■■■■■■	U	U	Unassignable
1499	■■□■■■■■■□□□□□□□■■■□□■■■■■■■□□□□■□■■■■■■■■■	EAI	EAI2	EAI
1500	■■□■■■■■■■■■■■■■■■■□□■■■■■■■□□□□□□□□■■■■■■■	U	U	animal
1501	■■□■■■■■■■■■■■■■■■■□□■■■■■■■□□□□■□■■■■□■■■■	EAI	EAI2	EAI
1502	■□□■■■■■■■■■■□■■■■■■■■■■■■■■□□□□□■□■■□□□■■■■	MANU	MANU1	MANU
1503	■■■■■■■■■■■■■□□■■■■■■■□■■■■■□□□□■□■■□■□■■■■	EAI	EAI6	EAI
1504	■■■■■■■■■■■■■□□■■■■■■■■■■■■■□□□□■□■■■■□■■■■	EAI	EAI5	EAI
1505	■■■■■■■■■■■■■□■■■■■■□□□□■■■■■■■■□□□□■■□■■■■	LAM	LAM9	LAM
1506	■■■■■■■■■■■■■□■■■■■■■■□■■■■■□□□□■□■■□■□■■■■	EAI	EAI6	EAI
1507	■■■■■■■■■■■■■□■■■■■■■■□■■■■■□□□□■□■■■■■■■■■	EAI	EAI6	EAI
1508	■■■■■■■■■■■■■□■■■■■■■■■■■■■■□□□□■□■■■■■■■■■	EAI	EAI5	EAI
1509	■■□■■■■■■■■■■■■■■■■□□■■■□■□□□□□□□□□□□□□□□□□□	U	U	animal
1510	■■□■■■■■■■■■■■■■■■■□□■■■■■□□□□□□□□■■■■■■■■■	U	U	animal

1511	■■□■■■■■■■□■■■■■■■■□□■■■■■■■□□□□■□■■■■■■■■■	EAI	EAI2	EAI
1512	■■□■□■■■■■■■■■■■■■■□□■■■■■■■□□□□■□■■■■■□□□■	EAI	EAI2	EAI
1513	■■■■■■■■■■□■■■■■■■■■■■■□■■■■■□□□□■□■■■■■■■■■	EAI	EAI6	EAI
1514	■■■■□■■■■■■■■■■■■■■■■■■■□□■■■■■■■■■■□□□□■■■■■■■	U	U	T
1515	■□□■■■■■■■■■■■■■■■■■■■■■■■■■□□□□□□■■■■■■■■■	U	U	Unassignable
1516	■■■■■■■■■□■■■■■■■■■■■■■■■■■■■■■■□□□■■■■■■■■	U	U	Unassignable
1517	■□□■■■■■■■■■■■■■■■□■■■■■■■■■□□□□■■■■■■■■■■■	U	U	EAI
1518	■□□■■■■■■■■■■■■■■■□■■■■■■■■■□□□□□□■■■■■■■■■	U	U	Unassignable
1519	□□□□□□□□□□□□□■■■■■■■■■■■■■■■□□□□□□■■■■■■■■■	U	U	Unassignable
1520	■□□■■■■■■■■■■■■■■■□■□■■■■■■■□□□□□□■■■■■■■■■	U	U	Unassignable
1521	■■■□□□□□□■■■■■■■□□□□□□□□■■■■□□□□□□■■■■■■■■■	U	U	Unassignable
1522	■■■■■■■■■■■■□■■■■■■■■□□□□■■□□□□■■□□■■■■■■■■■	MANU	MANU2	LAM
1523	■■■■■■■■■■■■■■■■■■■■□■■□■■■■■■■■□□■■■■■■■■■	MANU	MANU2	Unassignable
1524	■■■□□□□□□■■■■■■■■■■■■■□□■■■■□□□□□□■■■■■■■■■	U	U	Unassignable
1525	□■■■■■■■□□□■□■■■■■■■□□□□■■■■■■■■□□□□■■■■■■■	LAM	LAM3	LAM
1526	■■■□□□□□□□□□□■■■■■□□■■■■■■■■■■■■□□□□■■■■■■■	T	T1	T
1527	■■■■■■■■■■■■■■■■■□■■■■■■■□□□□□□■□□□□■■■■■■■	H	H1	X
1528	■■■■■■■□□□■■■■■■■■■■□□□□■■■■■■■■□□□□■■■■■■■	LAM	LAM9	LAM
1529	■■■■■■■■■■■■■■■■■■■■■■■■■■■■■■□■□□□□□□■■■■■	U	U	H
1530	■■■■■■■■■■■■■■■■■■■■□□□□■■■■■■■■□□□□■■■□□■■	LAM	LAM9	LAM
1531	■■■■■■■■■■■■■■■■■□■■■■■■■■■■■■■■□□□□□□□■■■■	U	U	X
1532	■■■■■■■■■■■■■■■■■□■■■■■■■□■■■■■■□□□□■■■■■■■	X	X1	X
1533	■■■■■■■■■■■■■■□□■■■■■■■■■■■■■■□■□□□□■■■■■■■	H	H3	H
1534	■■■■■■■■■■■■□■□□□■■■□□□□■■■■□■■■□□□□■■■□■■■	LAM	LAM5	LAM
1535	■■■■■■■■■■□■■■■■■■■■□□□□■■■■■■■■□□□□■■■■■■■	LAM	LAM9	LAM
1536	■■■■■■■■□□■■■■■■■■■■□□□□■■■■■■■■□□□□■■■■■■■	T	S	LAM
1537	■■■■■■■■□□□■□■■■■■■■□□□□■■■■■■■■□□□□■■■□■■■	LAM	LAM3	LAM
1538	■■■■■■■□□□□■□■■■■■■■□□□□□□□□□□□■□□□□■■■■■■■	H	H3	T
1539	■■■■■■□■■■■■■■■■■■■■■■■■■■■■■■□■□□□□■■■■■■■	H	H3	H
1540	□■■■■■■■■□■■■■■■■■■■□□□□■■■■■■■■□□□□■■■■■■■	LAM	LAM9	LAM
1541	□■■■■■■■□□■■■■■■■■■■□□□□■■■■■■■■□□□□■■■■■■■	LAM	LAM9	LAM
1542	□□■■■■■■■■■■■■■■■□■■■■■■■■■■■■■■□□□□■■□□□□■	X	X2	X
1543	□□□■■■■■□□□■■■□□□□□□□□□□□□□□□□□□□□□□□□□■■■■	U	U	Unassignable
1544	■■■■■■■■■■■■■■■■■■■■■■■■■■■■■■■■□□□□■□■□■■■	T	T2	T
1545	■■□□□□□□■■■■■■■■□■■■□□□□■■■■■■■■□□□□■■■■■■■	LAM	LAM9	LAM
1546	□□□■■■■■■■■■■■■■■■■■■■■■■■■■□□□□■□■■□□□□□■■	EAI	EAI5	EAI
1547	■■■■■■■■■■■■□■□■■■■■■■■■■■■■■■■■□□□□■■■■■■■	T	T3	T
1548	■■■■■■■■■■■■□□□□■■■□□□□□□□□□□□□□□□□□□■■■■□□	U	U	Unassignable
1549	■■■■■■■□■■■■■■■■■■■■□□□□■■□□□□■■□□□□■■■□■■■	LAM	LAM11	LAM
1550	□■■■■■■■■■■■■■■■■■■■■■■■■■■■■□■■□□□□■■■■■□□	T	T1	T
1551	■■■□□□□□■■■■■■■■■■■■■■□□□□□□□□□□□□■■■■■■■■■	CAS	CAS	CAS
1552	■■■■■■■■■■■■■■■■■■■■■■■■■□□□□□□■□□□□■■□□■■■	H	H1	T
1553	■■■■■■■□■■■■■■■■■■■□□□□□□□■■■■■■□□□□■■■■■■■	T	T1	T
1554	■□□□□□□■□■■■■■■■■■■■□□□□■■■■■■■■□□□□■■■□■■■	LAM	LAM4	LAM
1555	■■■□□□□□□□□□□□□□□■■□□□□□□■■■■■■■□□□□□■■■■■■	U	U	Unassignable
1556	■■■■■■■□■■■■■■■■■■■■■■■■■■■■■□■■□□□□■■■■■■■	T	T1	T
1557	■■■■■■■■■■■□■■■■■■■■■■■■■□□□□□□■□□□□■■■■■■■	H	H1	T
1558	■■■■■■■■■■■■■■■■■■■■■■■■■□■■■□■■□□□□■■■■■■■	T	T1	T
1559	■■■■■■■■■■■■■■■■■■■□□□□□□□□□□□□□□□□□□□■■■■■	U	U	Unassignable
1560	■■■□□□□■■■■■■■■■■■■■■■■■■■■■■■■■□□□□■■■■■■■	T	T1	T
1561	■■■■■■■■■■■■■■■■■■■■■■■■■□□□□□□■□□□□□■■■■■■	U	U	Unassignable
1562	■■■■■■■■■■■■■■■■■■□■■■■■■■■■■□□□□□□□□□□■■■■■	U	U	Unassignable
1563	■■■■■■■■■■■■■■■■■■■■□□■■■■□■■■■■□□□□■■■■■■■	T	T1	T
1564	■■■■■■■■■■■■■■■■■□■■■□■■■■■■■■■■□□□□■□■■■■■	X	X1	X
1565	■■■■■■■■■■■■■□□□□□□□□■■■■■■■■■■■□□□□■■■■■■■	T	T1	T
1566	■■■■□□■■■■■■■■■■■■■■■■■■■■■■■■■■□□□□■■■■■■■	T	T1	T

1567	■■■■■■■■■■■■■■■■■■■■■□□□■■■■□■■■□□□□■■■■■■■	T	T1	T
1568	■■■■■■■□□■■■■■■■■■□■■■■■■■■■□□□■□□□□■■■■■■■	H	H4	T
1569	■■■□□□□□□□□□□■■■■■□□□□□□□□□□□□□■□□□□■■■□□□□	H	H3	T
1570	■■■■■■■■■■■■■□□□□□□□□□□□□□□□□□□□□□□□□□□□□□□	U	U	Unassignable
1571	■■■■■■■■■■■■■■■■□■■■■■■■■□□■□□□□■□■■■■■■■■■	EAI	EAI4	EAI
1572	■■□■■■■■■■■■■■■■■■■■■■■■■■■■■■■□□□□■■■□■■□	T	T2	T
1573	■■■■■■■■■■■■■■■■■■□□■■■■■■■■□□□□■□■■□■■■■■■	EAI	EAI5	EAI
1574	■■■■■□□□□□□□□□□□□□□□□□□□□■■■■■■■■□□□□■■■■■■■	T	T1	T
1575	■■■■■■■■■■■■■■■■■■□■■■□□■■■■■■■■□□□□■□□□□□□	T	T4	T
1576	■■■■■■■■■■■■■■■■□□□□□■■□□■■■□■■■□□□□■■■■■■■	T	T1	T
1577	■■■■■■■■□□■■■■■■■□□□□□□□□■■■■■■■□□□□■■■■■■■	T	S	T
1578	■■■■■□■■■■■■■■■■■■■■■■■■■■■■■■■■□□□□■■■□■■■	T	T2	T
1579	■■□■■■■■■■■■■■■■■■■■■■■■■■■■■■■□□□□□■■■■■■■	U	U	animal
1580	■■■■■■■■■■■■■■■■■■■■■■□□■■■■■■■■□□□□■■■■■■■	T	T1	T
1581	■□■■■■■■■■□□■■■■■■■■■■■■■■■■□□□■□□□□■■■■■■■	H	H4	T
1582	■■■■■■■■■■■■■■■■■■□■■■■■■■■■■■■■□□□□■■■□□□□	T	T1	T
1583	■■■■■■■■■■■■■■■■■■■■■■■■■■■■■■■■□□□□■■■■□□□	T	T1	T
1584	■■■■□■■■■■■■■■■■■■■■□□■■□□□■■■■■□□□□■■■■■■■	T	T1	T
1585	■■■■■■■■■■■■■■■■■■■■■■■□■□□□□■□■□□□□■■■■■■■	H	H3	H
1586	□□■■■■■■■■■■■■■■■■■■■■■□■□□□□□□■□□□□■■■■■■■	H	H1	T
1587	■■□□■■■■□□■■■■■■■■■■■■■■□□□■■■■■□□□□■■■■■■■	T	S	T
1588	□□□□■■■■■■■□□■■■■■■■□□□□■■■■■■■■□□□□■■■■■■■	LAM	LAM9	LAM
1589	■■■■■■■■□□□□□□■■■■■□□□□□□■□□■■■■■□□□□■■■■■■■	LAM	LAM7	T
1590	■■■□□□□■■■■■■■■■■■□■■■□□□□□□□□□□□□■■■■■■■■■	CAS	CAS1	CAS
1591	■■■□□□□□□□■■■■■■■■■■■■□□□□□□□□□□□□■■□■■■■■■	CAS	CAS2	CAS
1592	■■■■■■■□□□□□■■■■■■■■□□□□□■□□□□□□□□□□□□□□■■■	U	U	AFRI
1593	■■■■□□□□□□□□□□□□□□□□□□□□□□□□□□□■□□□□■■■■■■■	H	H3	T
1594	■■■■■■■■■■□□□□□□□□□□□□□□■■■■■■■■□□□□■■■■■■■	T	T1	T
1595	■■□■■■■□□□■■■■□□■■■■□■■■■■■■■■■■■■■■■■□□□□□	animal	BOV	animal
1596	□■□□□□□□□□□□□□□□■■■■■■■■■■■□■■■■■■■■□■□□□□□	animal	CAP	animal
1597	■■■□□□■■■■■■■■■■■■■■■■■■■■■■■■■■□□□□■■■■■■■	T	T1	T
1598	□■□■■□□■□■■■■■■□■■■■■■■■■■■■■■■■■□■□■■□□□□□	animal	BOV1	animal
1599	□□□□□□□□□□□□□□□□■■■■■■■■■■■□■■■■■■■■■■□□□□□	animal	BOV	animal
1600	□■□□□□□□□□□□□□□□■■■■■■■■□□□□□□□□□□■■■■□□□□□	animal	CAP	animal
1601	■■□■■■■■□■■■■■■□■■■■□■■■■■■■■■■■■■■■■□□□□□□	animal	BOV	animal
1602	■■□■■■■□□□□□□■□□■■■■■■■■■■■■■■■■■■■■■■□□□□□	animal	BOV	animal
1603	■■□■■□■■□■■■■■■□■■■■■■■■■■■■■□■■■■■■■■□□□□□	animal	BOV1	animal
1604	□■□□□□□□□□□□□□□□■■□■■■■■■■■□■■■■■■■■■■□□□□□	animal	CAP	animal
1605	■□□□□■■■□■■■■■■□■■■■□■■■■■■■■■■■■■■■■■□□□□□	animal	BOV	animal
1606	■■□□□□□■■■■■■■■■■■■■■■□□□□□□□□□□□□□□■■■■■■■	CAS	CAS	Unassignable
1607	■■□□□□□□■■■■■■■■■■■■□□□□■■□□□□■■□□□□■■■■■■■	LAM	LAM11	LAM
1608	■■■■■■■■■■□□□□■■■■■■□□□□■■■■■■■■□□□□■■■□□□□	LAM	LAM9	LAM
1609	■■■■■■■■■■■□□■■■■■■■■■■■■■■■□□□□■□■■■■■■■■■	EAI	EAI5	EAI
1610	■■■■■□□■■■■■■■■■■■■■□□□□■■■■□■■■□□□□■■■■■■■	LAM	LAM6	LAM
1611	■■■■■■■■□■■■■■■■■■■■■■■■■■■■■□■□□□□■■■■■■■	H	H3	H
1612	□□□■■■■■■■□■■■■■■■■■■■□■■■■□□□□□□□■■□■■■■■■	U	U	Unassignable
1613	□□■■■■■■■■■■■■■■■■■■■■■■■■■■■■■■□□□□■■■□■■■	T	T2	T
1614	■■■■■■■■■■■■■■■■■□■■□□□□■■■■■■■■□□□□■■■□□□□	X	X1	Unassignable
1615	■■■■■■■■■■□□□□■■■■■■■■■■■■■■■■■■□□□□■■■□■■■	T	T2	T
1616	□□□□□□□■■■■■■■■■■■■■■■□□□□□□□□□□□□■■■■■■■■■	CAS	CAS	CAS
1617	■■■□□□□□□□□□□■■■■□■■■■■■■■□■■■■■□□□□■■■■■■■	X	X3	X
1618	■□■■■■■■■■■■■■■■■■□□□□□□□□□□□□□□□□□□□□□□□■■	U	U	Unassignable
1619	□□□□□□□□□□□□□□□□■□■■■■■■■■■■■■■■□□□□■■■□■■■	X	X1	X
1620	■■■■■■■■■■■■■■■■■■■■□□□□■□□□■■■■□□□□■■■■■■■	LAM	LAM9	LAM
1621	■■■□■■■■■■■■□■■■■■■■■■■■■■■■■■■■□□□□■■■□■■■	T	T2	T
1622	■■■■■□□□■■■■■■■■■■■■■■■■■■■■■■■■□□□□■■■□■■■	T	T2	T

1623	■■■■■■■■■■■■■■■■■■■■□■■■■■■■■■■■■□□□□■■■□□□□	T	T1	T
1624	■■■■■■■■□□□■■■■■■■■■■□□□□■■■■□■■■□□□□■■■■■■■	LAM	LAM3	LAM
1625	■■□■■□■□□□□□■■■□■■■■■■■■■■■■■■□□□□□□■■□□□□□	animal	BOV2	animal
1626	■■■■■■■■■■■■■■■■■■■■■■■■■■□■■■■■□□□□■■■■■■■	T	T1	T
1627	■■■■■■■■■□□■□■■■■■■■■■■■■■■■■■■■■□□□□■■■□■■■	T	T2	T
1628	□□□■■■■■■■■■□■■■■■■■■■■■■■■■□□□□■□■■■■■■■■■	EAI	EAI5	EAI
1629	■■■■■■■■■■■■■■■■■■■■■■■■■□□□□□□■□□□□■■■■□■■	H	H1	T
1630	■■■■■■■■■■■■■■■■■■■■□□□□□□■■■■■■□□□□■■■■■■■	T	T1	T
1631	■■■■□■■■■■■■■■■■■■■■■■■■■■■■■■■□□□□■□■■■■■	T	T1	T
1632	■□■■■■■■■■■■■■■■■■■■■■■■■■□□□□□□□□□□□■■■■■■	U	U	Unassignable
1633	■■□□■■■■■■□□□□□□■■■■□□□□■■■■■■■■□□□□■■□■■■■	LAM	LAM9	LAM
1634	■■■■■■■■■■■■■■■■■■■■■■■■■■■■■■□■□□■■■■■■■■■	MANU	MANU2	H
1635	□■□□□□□□□□□□□□□□■■■■■■■■■■■□■■■■□□■■■■□□□□□	MANU	MANU2	animal
1636	□■□□□□□□□□□□□□□□■■■■■■■■■□□□□□■■■■□■■■□□□□□	animal	CAP	animal
1637	■■□■■■■■□■■■■■■□■■■■■■■■■■■■□■■■■■■■■■□□□□□	animal	BOV1	animal
1638	■□■■■■■■■■■■■■□■■■■■■■■■■■■■■■■■□□■■■■■■■■■	MANU	MANU2	Unassignable
1639	■■■■□□□■■■■■■■■■■■■■□□□□■■■■■■■■□□□□■■■■■■■	LAM	LAM9	LAM
1640	■■■■■■■■■■■■■■■■■■■■■■■■■■■■■■□■□□□□■■■■□□□	H	H3	H
1641	■■■■■■■■■■■■□■■■■■□□■■■■■■■■■■■■□□□□■■■■■■■	T	T3	T
1642	■■■□□□□□□□□□□□■■■■■■■■■■■■■■■■■■□□□□■■■■■■■	T	T1	T
1643	■■■■■■■■■■■■■■■■■■■□□□■□□■■■□■■■□□□□■■■■■■■	T	T1	T
1644	■■■■■■■■■■■■■■■■■■■□□□□□■□□□□□□■□□□□■■■■■■■	H	H1	T
1645	□■■■■■■■■■■■■■□■■■■■■■■■■■■■■■■■□□□□■■■■■■■	T	T1	T
1646	■■■■■■■■■■■■□■■■■■■■□□□□■■■□□□□■□□□□■■■■■■■	H	H3	LAM
1647	■■■■■■■■■■■■■■■■■■■□□■■■■■■■■■■■□□□□■■■□■■■	T	H37Rv	T
1648	■□□□□□□□□□□□□□■■■■■■■■■■■■■■□□□□■□■■■■■■■■■	EAI	EAI5	EAI
1649	■■■■□■■■■■■□■■■■■■■■■■■■■■■■□□□□■□■■■■■□■■■	EAI	EAI1	EAI
1650	■■■□■□□□□□□□□□□□□■■□□□□□□□□□□□□□□□□□■■■■■■■	U	U	Unassignable
1651	□□□□□□□□□□□□□□□□□□□□□□□□□□□□□□□□□□■■■■□■■□■	BEIJ	BEIJ	BEIJ
1652	■□□■■■■■■■■■■■■■■■■■■■■■■□□□□□□■□□□□■■■■■■■	H	H1	T
1653	■■■□■■□□□□□□□□□□□□□□□□□□□□□□□□□□□□□□□□■■■■■	U	U	Unassignable
1654	■■■■■■■■■■■□■■■■□□□□□■■■■■■■□□□□■□■■■■□■■■■	EAI	EAI5	EAI
1655	■■■■■■■■■■■■□■□□■■■■■■■■■■■■■■■■□□□□■■■■■■■	T	T3	T
1656	■■■■■■■■■■■■■■□■■■■■■■■■■□□□□□□□□□□□□□□□□□□□	U	U	Unassignable
1657	■■■■■■■■■■■■■■■■■■□■■■■■■■■■□□□□□□□□□□■■■■■■	U	U	Unassignable
1658	■■■■■■■■■■■■■■■■■■■■■□□■■□□□□□□■□□□□■■■■■■■	H	H1	T
1659	■■■■■■■■■■■■□□□□■■■■■□□□□□□□□□□□□□□□□□□□□□□	U	U	Unassignable
1660	■■■■■■■■■■■■■■■■■■■■□□□□□□□□□□■■□□□□■■■□■■■	T	T2	T
1661	■■□■■■■■■■■■□■■■■■■■□□□□■■■■■■■■□□□□■■□■■■■	LAM	LAM2	LAM
1662	■■□□□□□□□□□□□□□□□□□□□□□□□□□□□□■■□□□□■■■□■■■	T	T2	T
1663	■■■■■■■■■■■■■■■■■■■■□□□□■■■■□■□■□□□□■■■■■■■	H	H3	Unassignable
1664	■■■■■■■■■■■■■■■■■■■■□□□□□□□■□■■■□□□□■■■□■■■	T	T2	T
1665	□■■■■■■■■■□□□□□□□□□□□□□□□□□□□□□□□□□□□■□■■■	U	U	Unassignable
1666	■■□■■□■□□□□□■■□□■■■■■■■■■■■■■■■■■■■□■■□□□□□	animal	BOV	animal
1667	■■□□□■■■□■■■■■■□■■■■■■■■■■■■■■■■■■■■□■□□□□□	animal	BOV1	animal
1668	■■□■■■■■□■■■■■□□■■■■□■■■■■■■■■■■■■■□□■□□□□□	animal	BOV	animal
1669	■■□■■■■■□■■■■■□□■■■■□■■■■■■■■■■■■■■□■■□□□□□	animal	BOV	animal
1670	□□□■■□■□□□□□■■■□■■■■■■■■■■■■■■■■■■■■■■□□□□□	animal	BOV	animal
1671	□■■■■■■■■■■■■■□■■■■■□□□□■■■■■■■■□□□□■■■■■■■	LAM	LAM9	LAM
1672	■■■■■■■■□□□■■■■■■■■■■■■■■□■■■■■■□□□□■■■■■■■	T	T1	T
1673	■■□□□■■□□□■■■■■□■■■■■■■■■■■■■■■■■■■■■■□□□□□	animal	BOV	animal
1674	□□□□□□□□□□□□□□□□□□□□□□□□□□□□□□□□□□■■■■■■■□■	BEIJ	BEIJ	BEIJ
1675	■■■□□□□■■□■■■■□■■■■□□□□□□□□□□□□□□□□■■■■■■■■	CAS	CAS1	CAS
1676	■□□■■■■■■■■■■■■■■■□■■■■■■■■■■□□□■□■■□□□■■■■	MANU	MANU1	MANU
1677	■■■□□□□■■□■■■■■■■■■□□□□□□□□□■□□□□□■■■■■■■■■	U	U	CAS
1678	■■■■■■■■■■■■■■■■□□□■■■■■■■■■■■□■□□□□■■■■■■■	H	H3	H

1679	■■■■■■■■■■■■■■□■■■■■■□■■■■■■■■■■□□□□■■■■■■■	T	T1	T
1680	■□□■■■■□■■■■■■■■■■■■■■■■■■■■□□□□■□■■□□□■■■■	EAI	EAI3	EAI
1681	□■■■■■■■■■■■■■■■■■■■■■■■■□□□□□□■□□□□■■■■■■■	H	H1	T
1682	■■□■■□□■□■■■■■■□■■■■□■■■■■■■■■■■■■■■■■□□□□□	animal	BOV1	animal
1683	■■□■■■■■■■■■■■■■■■■■■■■■■■■■■□■■□□□□■■■■■■■	T	T1	T
1684	■■■■■■■■□□□□□□□□□□□□□□□□□□□□□□□□□□□□□□□□□□□	U	U	Unassignable
1685	■■■■■■■■□□□□■■■■■■■■□□□□■■■■■■■■□□□□■■■■■■■	LAM	LAM9	LAM
1686	■■■■■■■■■■■■■□□□□□□□□□□□■□□□□□□■□□□□■■■■■■■	H	H1	T
1687	■■■■■■■■■■■■■■□■■■■■■■■■■□■■■■■■□□□□■■■■■■■	T	T1	T
1688	■■■■■■■■■■■■■■■■■■■□□□□□□■■■■■■■□□□□■■■■■■■	T	T1	T
1689	■■■■■■■■■■■■■■■■■■■■□□□□■■■■■□□□□□□□□□□□■■■	U	U	LAM
1690	■■■■■■■■■■■■■■■■■■■■■■■■■■■■■■■■□□■□■■■■■■■	MANU	MANU2	Unassignable
1691	■■□■■■■■■■■■□■□■■■■■□□□□■■■■■■■■□□□□■■■■■■■	LAM	LAM2	LAM
1692	■□□□□■■■■□□■□■■■■□■■■■■■■■■■■■■■□□□□■■■□■■■	X	X1	X
1693	■■■□■■■■■■■■□■■■■■■■□□□□■■■■■■■■□□□□■■■■■■■	LAM	LAM5	LAM
1694	□■□■■■■■■■■■□■■■■■■■□□□□■■■■■■■■□□□□■■■■■■■	LAM	LAM5	LAM
1695	■■□■■■■■■■■■□■■■■■■■□□□□■■■■■■■■□□□□■■■■■■□	LAM	LAM2	LAM
1696	■■■□□□■■■■■■□■■■■■■■□□□□■■■■■■■■□□□□■■■■■■■	LAM	LAM5	LAM
1697	■■■■■■■□■■■■■■□■■■■■□□□□■■■■■■■■□□□□■■□■■■■	LAM	LAM9	LAM
1698	■■■■■■■■■■■■□■□■■■■■□□□□■■■■■■□□□□□□□□□□■■■	U	U	LAM
1699	■■■■■■■■■□■■■□□■■■■■□□□□■■■■■■■■□□□□■■□■■■■	LAM	LAM9	LAM
1700	■■■■□■□■■■■■■■■■■■■■■■■■■■□■■■■■□□□□■■■■■■■	T	T1	T
1701	■■■■■■■■■□■■□□■■■■■■□□□□■■■■■■■■□□□□■■■■■■■	LAM	LAM9	LAM
1702	□□□□■■■■■■■■□■■■■■■■□□□□■■■■■■■■□□□□■■■■■■■	LAM	LAM5	LAM
1703	■■□■■■■■■■■■□■■■■■■■□□□□■■■■□■■■□□□□■■■■■■■	LAM	LAM2	LAM
1704	■□□■■■■■■■■■□■□■■■■■□□□□■■■■■■■■□□□□■■■■■■■	LAM	LAM5	LAM
1705	■■■■■■■■■■■■□□□□■■■■■■■■■■■■□□■■□□□□■■■■■■■	T	T1	T
1706	■■■□□■■■■■■■■■■■■■■■□□□□■■■■■■■■□□□□■■■■■■■	LAM	LAM9	LAM
1707	■■□■■■■■■■■■■■■■■■■□□□□□□□□■■■■■□□□□■■■■■■■	T	T1	T
1708	■■■■■■■■□■■■■■■■■■■■□□□□■■■■■■■■□□□□■■■■■■■	LAM	LAM9	LAM
1709	■■■■■■■■■■■■□■■■■■■■□□□□■■□■■■■■□□□□■■■■■■■	LAM	LAM5	LAM
1710	■■■■■■■■■■■■□■■■■■■■□□□□■■■■■■■■□□□□■■■□■■■	LAM	LAM4	LAM
1711	■■□■■■■■■□■■□■■■■■■■□□□□■■■■■■■■□□□□■■■■■■■	LAM	LAM2	LAM
1712	■■□■■■■■■□■■□□■■■■■■□□□□■■■■■■■■□□□□■■■■■■■	LAM	LAM1	LAM
1713	■■□■■□□■■■■■■■■■■■■■□□□□■■■■■■■■□□□□■■■■■■■	LAM	LAM1	LAM
1714	■■■■■■■■■■■■■□□□□□■■□□□□■■■■■■■■□□□□■■■■■■■	LAM	LAM9	LAM
1715	■□□□■■■■■■■■■■■■■■■■□□□□■■■■■■■■□□□□■■■□■■■	LAM	LAM4	LAM
1716	■■■■■□■■■■■■□■■■■■■■□■■■■□■■□■■■□□□□■■■■■■■	T	T3	T
1717	■■■■■■■■■■■■■■■■■□■■■■■■■■■■□□■■□□□□■■■■■■■	X	X1	X
1718	■■□■■■■■■■■■■■■■■□■■□□□□■■■■■■■■□□□□■■■■■■■	X	X1	Unassignable
1719	■■□■■■■■■□■■□■■■■■■■□□□□■■■■■■■■□□□□■■□■■■■	LAM	LAM2	LAM
1720	■■■■■■■■■■■■■■□■■■■■■■■■■□□■□□□□■□■■■■■■■■■	EAI	EAI4	EAI
1721	■■■■■■■■■■■■■□□■■□■■■■■■■■■■■■■■□□□□■■■■■■■	X	X1	X
1722	■■□■■□■□□□□□■■■□■■■■□■■■■■■■■■■■■■■□■□□□□□	animal	BOV2	animal
1723	■■□■■□■□□□□□■■■□■■■■■■■■□□□□□□□■■■■■■■□□□□□	animal	BOV2	animal
1724	■□□□■■■□■■■■■■■□■■□□□□■■■■■■■□□□□■□□■■■■■■■	U	U	animal
1725	■■■□□■■■■■■■■■■■■■■■■■□■■■■■□□□□■□■■■■■■■■■	EAI	EAI6	EAI
1726	■■■■■■■■■■□■■■■■■■■■■■■■■■■■□□□□■□■■□■■■■■■	EAI	EAI5	EAI
1727	■■■■■■■■□□■■■■■■■■■■■□□□□■■■■■■■□□□□■■■■■■■	T	S	T
1728	■■■■■■■■□□■■■■■■■■■■□□□□□■■■■■■■□□□□■■■■■■■	T	S	T
1729	■■■□□□□□□□□□□□□□□□□□□□□□■□□□□■■■■■■■■■■■■■■	U	U	Unassignable
1730	■■□■■■■■□■■■■■■□■■■■■■■■■■■■■■■□□■■■■■□□□□□	animal	BOV1	animal
1731	■□■■■■■■■■■■■■■■■■■■■■■■■□□■□□□□■□■■■■■■■■■	EAI	EAI4	EAI
1732	■□■■■■■■■■■■■■□■■■■■■■■■■■■■□□□□■□■■■■■□■■■	EAI	EAI1	EAI
1733	□■■■■■■■□■■■■■■■□□■■■□■■■■■■■■■■□□□□■■■■■■■	T	T1	T
1734	□□□■■■■■■■■■■■□■■■■■■■■■■■■■□□□□■□■■■■■□■■■	EAI	EAI1	EAI

1735	■■■■■■■■■■■■■■■■■■■■■■■■■□□□■■□■□□□□■■■■■■■	H	H3	H
1736	■■■■■■■■■■■■■■■■■■■■■□□□□□□□□□□□□□□□□■■■■■■	U	U	Unassignable
1737	■■■■■■■■■■■■■■□□□□□□□□□■■■■■■■■■□□□□■■■■■■■	T	T1	T
1738	■■■■■■■■■■■■■■□□□□□□□■■□□■■■■■■■□□□□■■■■■■■	T	T1	T
1739	■□■■■■■■■■■■■■■■■■□■■■■■■■■■■■■■□□□□■■■□□□□	T	T1	T
1740	■□□■■□■■■■■■■■■■■■■■■■■■■□□□□□□□□□□□□□□■■■■	U	U	Unassignable
1741	■■□□□□□□□□□□□□□□□□□□□■■■■■■■□□□□■□■■■■■■■■■	EAI	EAI5	EAI
1742	■■■■■■■□■■■■■■■■■■■■■■■■■□□□□□□■□□□□■■■■■■■	H	H1	T
1743	■■■■■□■■■■■■■■■■■■■■■■■■■■■■□■□□□□■■□□■■■	H	H3	H
1744	■□■■■■■■■■■■■■■■■□■■■■■■□□□□□□□□□□□□□□□□□□□	U	U	X
1745	■■■■■■□■■■■■□■■■■■■■■■■■■■■■■■■■■□□□□■■■■■■■	T	T3	T
1746	■□□■■■■■□□■■■■■■■■■■■□■■■■■■■■■■□□□□■■■■■■■	T	S	T
1747	■■■□□■■■■■■■■■■■■■■■■■□■■■■■■■■■□□□□■■■■■■■	T	T5	T
1748	■■■■■■■■■■■■■□■■■■■■■■■■■■■■■□□■□□□□■■■■■■■	H	H3	T
1749	■■■■■■■■■■■■■■■■■■■■■□■■■□□■■■□■□□□□■■■■■■■	H	H3	H
1750	■■■■■■■■■■■■■■□■■■■■□□□□■■■■■■■■□□□□■■■□■■■	LAM	LAM4	LAM
1751	■■■□□□□□□□□□■■□■■□■■■■■■■■■■■■■■■□□□□■■■■■■■	X	X3	X
1752	■■□■■■■■■■■■■■■■■■■■□□□□■■■■■■■□□□□□■■■■■■■	U	U	LAM
1753	■■■□□□□□□□□□□■■■■■■■■■■■■■■■■■■■□□□□■■■■■■■	T	T1	T
1754	■■■■■■■■■■■■■■□■■■■■■■■■■■■■■■■■□□□□■■□□□□■	T	T1	T
1755	■■□■■■■■■■■■■■■■■■■■□□□□■■■■□■■■□□□□■■■■■■■	LAM	LAM1	LAM
1756	■■■□□□□□□□□□□■□■■□■■■■■■■■■■■■■■□□□□■■■■■■■	X	X3	X
1757	■■■■□■■■■■■■■■■■■■■■□□□□□□□□□□□□□□□□□□□□□□□	U	U	Unassignable
1758	■■■■■■■■■■■■■■■■■■■■□□□□■■■■□■■□□□□□■■■■■■■	U	U	LAM
1759	■■■■■■□■□□□■■■■■■■■■□□□□■■□■■■■■□□□□■■■■■■■	LAM	LAM8	LAM
1760	■□□■■■■■□□□■■■■■■■■■□□□□■■■■■■■■□□□□■■■■■■■	LAM	LAM3	LAM
1761	■■□■■■■■■■■■■■□■■■■■■■■■■■■■■■■■□□□□■■■■■■■	T	T1	T
1762	■□□■■■■■■■■■■■■■■■■■□□■■■■■■□□□□■□■■□□□■■■■	EAI	EAI3	EAI
1763	■□□□■■■■■■■■■■■■■■■■■■■■■□□□■■□■□□□□■■■□■■■	H	H3	H
1764	■□□□□□□□□□□□□■■■■■■■■■■■■■■■□□□□□■□■■■■■■■■	U	U	Unassignable
1765	■■■□□□□■■□■■□■■■■■■□□□□□□□□□□□□□□□□■■■■■■■■	CAS	CAS1	CAS
1766	■■■■■□□□□■■■■■■■■■■■□□□□■■■■■■■■□□□□■■■■■■■	LAM	LAM9	LAM
1767	■■■■■■■■■■■■■■■□□□□□□□□□■■■■■■■■□□□□■■■■■■■	T	T1	T
1768	■■■■■■■■■■■■■■■■■■□■□□□□■■■■□■■■□□□□■■■■■■■	LAM	LAM6	LAM
1769	■■□■■■■■■■■■■■□□□■■■□□□□■■■■■■■■□□□□■■■■■■■	LAM	LAM1	LAM
1770	□□□□□□□□□□□□□□□□□□□□□□□□■□□□□□□■□□□□■□■■■■■	H	H2	T
1771	■■■■□■■■■■■■■■■■■■■■■■□□■□□□□□□■□□□□■■■■■■■	H	H1	T
1772	■■■■■■■■□□■■■■■■■■■■■■■■■□□□□□□■□□□□□□□■■■■	U	U	Unassignable
1773	■■■■■■■■■■■■■■■■■■■□■■□□■■■■■■■■□□□□■□□■■■■	T	T1	T
1774	■■■■■■■■■■■■■■■■■■□■■■■■■■■□■■■■■□□□□■■■■■■■	T	T4	T
1775	□■□■■■■■■■■■■■■■■■■□□■■■■■■■□□□□■□■■■■■■■■■	EAI	EAI2	EAI
1776	■■■■■■■■□□■■■■■■■■■■■■■□□□□□□□□□□□□□□□■■■■■	U	U	Unassignable
1777	■■■□□□□■■■■■■■■■■□□□□□□□□□□□□□□□□□□□□□□□□■■	CAS	CAS1	CAS
1778	■■■■■■■■■■■■■■■□□□□□□□□□□□□□□□□□□□□□□■■■■■■	U	U	Unassignable
1779	■■■■■■■■□■□□■■■■■■■■■■■■■■■■■■■■□□□□■■■■■■■	T	T1	T
1780	□□□□□□□□□□□□□□□□□□□□□□□□□□□□□□□□□■□□■■■■■■■	U	U	Unassignable
1781	■■□■■■■■■■■■■■■■■■■□□■■■■■■■□□□□■□■■■■■■■■□	EAI	EAI2	EAI
1782	■■■■■■□□□■■■■■■■■■■■□□□□■■■■■■■■■■■■■■□■■■■	AFRI	AFRI1	LAM
1783	■■■■■■■■□□■■■■■■■■■■■■□□□■■■■■■■□□□□■■■■■■■	LAM	LAM10	T
1784	■■□■■■■■□■■■■■□□□□■■■■■■■■■■■■■■■■■■■■□□□□□	animal	BOV	animal
1785	■■■■■■□□□□□□□□□□□□□□□□□□■□□■■■■■□□□□■■■■■■■	T	T1	T
1786	□□□□■■■■■■■□■□□■■■■■■■■■■■■■■■■■□□□□■■■■■■■	T	T1	T
1787	■■■□□□□■■■■■■■■■■■■■■■□□□□□□□□□□□□■□□□■■■■■	CAS	CAS1	CAS
1788	■■■■■■■■■■■■□□□□■■■□□□□□□□□□□□□□□□□□□■■■■□■	U	U	Unassignable
1789	■■■□□□□■■■■■■■■■■■■■■■□□□□□□□□□□□□□□□□■■■■■	CAS	CAS1	CAS
1790	■■■■■■■■■■■■■■■■■■□□□□□□□□□□■■■□□□□■■■■■■■	T	T1	T

1791	■■□■■■■■■■■■■■■■■■■■□□□□■■■□□■■■□□□□■■■■■■■	LAM	LAM1	LAM
1792	■□■■■■■■■■■■■■■■■■■■□□□□■■■■■■□■□□□□■■■■■■■	H	H3	Unassignable
1793	■■■■■■■■■■■■■■■■■■■■■■■■■■■■■■■■□□□□□□□□□□□	U	U	T
1794	□■□■■■■■■■■■■■■■■■■■■□■■■■■■■■■■□□□□■■■□■■■	T	T3	T
1795	■■■■■■■■■■■■■■■■■■■■■■□□□□□□□□□□□□□□□■■■■■■	U	U	Unassignable
1796	□□□□□□□□□□□□■■■■■■■■■■■■■■■■■■■■□□□□■■■■■■■	T	T1	T
1797	■■■■■■■■■■■■■■■■■■■■■■■■■■■■■□■■□□□□■■■□■■■	T	T2	T
1798	■□■■■■■■■■■■■■■■■■■■■■□□■□□□□□□■□□□□■■■■■■■	H	H1	T
1799	■■■■■■■■■■■■■■■■■■□■■■□□■■■■■■■■□□□□■□□□■■■	T	T4	T
1800	■■■■■■■■■■■■■■■■■■■□□□□□■■■■■■■■□□□□■■■■■■■	T	T1	T
1801	■■■■■■■■■■■■■■■■■■■■■■■■■■■■□□□□■□■■■■■□■■□	EAI	EAI1	EAI
1802	■■■■■■■■■■■■■■□■■■■■■■■■■■■■■■□■□□□□■■□□■■■	H	H3	H
1803	■■□□□■■■■■■■■■■■■■■■□□□□■■■■■■■■□□□□■■■■■■■	LAM	LAM9	LAM
1804	■■■■■■■■■■■■■■■■■■■□■■■■■■■■■■□■□□□□■■■■■■■	H	H3	H
1805	■■■■■■■■■■■■■■■■■■■■■■■■■■■■■■■□□□□□■■□■■■■	U	U	Unassignable
1806	■■□■■■■■■■■■■□□■■■■■■■□■■■■■■■■■□□□□■■■■■■■	T	T5	T
1807	■■■■■■■■■■■■■■■■■■■■■■■■■□□□□□□■□□□□■□■■■■■	H	H1	T
1808	■■□■■■■■■■■■■■■■■■■■■■□■■■■■■■■■□□□□■■■■■■■	T	T5	T
1809	■■■□□□■■■■■■■■■■■■□■■■□□■■■■■■■□□□□□■□□■■■■	U	U	Unassignable
1810	□■□□□□□□□□□□□□□□□■□■■■■■■■■□■■■■■■■□■■□□□□□	animal	CAP	animal
1811	■■□■■■■■■□■■□□□□□□□□□□□□■□□■■■■■□□□□■■■□■■■	T	T2	T
1812	■■■■■■■■■■■■■■■■■■■■□□■■■■■■■■□■□□□□■■■■■■■	H	H3	H
1813	■■■■□■■■■■■■■■□■■■■■■■■■■□□□□□□■□□□□■■■■■■■	H	H1	T
1814	■■■■■■■■■■■■■■■■■■■■□□□■■■■■□■■■□□□□■□■■■■■	T	T1	T
1815	■■□■■■■■■■■■■■■■■■■■□□□□■■□■■■■■□□□□■■■■■■■	LAM	LAM1	LAM
1816	■■□■■■■■□■■■■■■□■■■■□■■■■■■■■□□□□□■■■■□□□□□	animal	BOV1	animal
1817	■□□■■■■□□■□■■■□□■■■■■■■■■■■■■■■■■■■■■■□□□□□	animal	BOV	animal
1818	■■□■□□□□□□□□□□■□■■■■■■■■■■■■■■■■□□■■■■□□□□□	animal	BOV3	animal
1819	■□□■■■■■□■■■■■□□■■■■□■■■■■■■■■■■■■■■□■□□□□□	animal	BOV	animal
1820	■■□■■■■■□■■■■■■□■■□■□■■■■■■■■■■■■■■■□■□□□□□	animal	BOV1	animal
1821	■■■■■■■■■□■■■□□■■■■■■■■■■■■■■■■■□□□□■■■■■■■	T	T1	T
1822	■■■■■■■■■■■■■■■■■■■■■■■■■□□□□□□■□□□□■■■■■■□	H	H1	T
1823	■■■■■■■■■■■■■■■■■□■■■■■■■■■■■■■■□□□□■■■■■■□	X	X1	X
1824	■■■■■□□□□□■■■■■■■■■■■■■■■■■■■■■■□□□□■■■□□□■	T	T1	T
1825	□□□□□□□□□□□□□□□□□□□□□□□□■□□□■□□■□□□□■■■■■■■	H	H3	T
1826	■■■■■■■■■■■■□□■■■□■■■■■■■■■■■■■■□□□□■■■■■■■	X	X1	X
1827	■■■■■■■■■■■■□□□□■■■■□□□□□□□□□□□■□□□□■■■■■■■	H	H3	T
1828	■■■■■■■■■■■■■■■■■□□■□□□□■■■■■■■■□□□□■■■■■■■	LAM	LAM9	LAM
1829	■■■■■■■□■■■■■■□■■■■■■■■■■■■■■■■■□□□□■■□■■■■	T	T1	T
1830	■■■■■■■■□□□■■■■■■■□■□□□□■■■■■■■■□□□□■■■■■■■	LAM	LAM3	LAM
1831	■■■■■■■■■■■■■■■■■■■■□■■□□■■■■■■□□□□□■■■■■■■	U	U	Unassignable
1832	■■■■■■■■■■■■■■■□■■■■□□□□■■■■■■■■□□□□■■■■■■■	LAM	LAM9	LAM
1833	■■■■■■■■■■■■□□□■□■□□□■■■■■■■□■■■□□□□■□■■■■■	T	T1	T
1834	□■■■■■■■■■■■□■■■■■■■■■■■■■■■■■■■□□□□■■■■■■■	T	T3	T
1835	■■■■■■■■■■■■□□□□■■□■■■■■■■■■■■■■□□□□■■■□■■■	T	T2	T
1836	■■■■■■■■■■■■■■■■■■□□□□□□■■■■■□■■□□□□■■■■□■■	T	T1	T
1837	□□□□□□□□□□□□□□□□□□□□□□□□□□□□■□□□□□■■■■■■■■■	U	U	Unassignable
1838	■■■■■■■■■□■■■■□■■■■■□□□□■■■■■■■■□□□□■■■■■■■	LAM	LAM9	LAM
1839	■■■■■■■■■□■■□■□■■■■■□□□□■■■■■■■■□□□□■■■■■■□	LAM	LAM5	LAM
1840	■■■■■■■■□□■■□■□■■■■■■■■■■■■■■■■■□□□□■■■■■■■	T	S	T
1841	■■■■■■■■□□□■■■■■■■■■□□□□■■■■■■■□□□□□■■■■■■■	U	U	LAM
1842	■□□■■■■■□■■■■■□□■■■■■■■■■■■■■■■■■■■■■■□□□□□	animal	BOV	animal
1843	■■■■□□□□□■■■■■■■■■■■■■■■■■■■■■■■□□□□■■■□■■■	T	T2	T
1844	■■■■■■■■■■■■■■□□■■■■□□□□■■■■■■■■□□□□■■■■■■■	LAM	LAM9	LAM
1845	□□□□□□□□□□□■■■■□■■■■■■■■■■■■■■■■■■■■■■□□□□□	animal	BOV	animal
1846	■■□■□□□□□□□□□□■□■■■■■■■■■■□■■■■■■■■■■■□□□□□	animal	BOV3	animal

1847	■■□■■■■■□■■■■■■□■■■■□■■■■■■□■■■■■■■■■■□□□□□	animal	BOV1	animal
1848	■■□■■■■■□■■■■□□□□□■■□■■■■■■■■■■■■■■■■■□□□□□	animal	BOV	animal
1849	■■□■■■■□□■□■■■■□■■■■■■■■■■■■■■■■■■■■■■□□□□□	animal	BOV	animal
1850	■■□■■□■□□□□□■■■□■■■■■■■■■■□□□■■■■■■■■■□□□□□	animal	BOV2	animal
1851	■□□■■□■□□□□□■■■□■■■■■■■■■■■■■■■■■■■■■■□□□□□	animal	BOV	animal
1852	■■□■■■■■□■■■■■■□■■■■■■■■□□□□□□□□□□■■■■□□□□□	animal	BOV1	animal
1853	■■□■■■■□□■■■■■■□■■■■□■■■■□□□■■■■■■■■□■□□□□□	animal	BOV	animal
1854	■■□■■■■■□■■■■■■□■■■■■■■■□□□□□□□□□□□□■■□□□□□	animal	BOV1	animal
1855	■■□■■□■□□□□□■■■□■■■■■■■■■■■■■■■■■□■■■■□□□□□	animal	BOV2	animal
1856	■■□■■□■□□□□□■■■□■□■■■■■■■■■■■■■■■■■■■■□□□□□	animal	BOV2	animal
1857	■■□■■■■■□■□■■■■□■■■■■■■■■■■□□■■■■■■■■■□□□□□	animal	BOV1	animal
1858	□■□■■□■□□□□□■■■□□■■■■■■■■■■■■■■■■■■■■■□□□□□	animal	BOV2	animal
1859	■■□□■□■□□□□□■■■□■■■■■■■■■■■■■■□□□□□□■■□□□□□	animal	BOV	animal
1860	■■□■■□■□□□□□■■■□■■■■■■■■■■■■■■■□□□□■■■□□□□□	animal	BOV2	animal
1861	■■□■■□■□□□□□■■■□■■■■■■■■■■■■■■□□□■■■■■□□□□□	animal	BOV2	animal
1862	■■□■□□□□□□□□□□□□□□□□□□■■■■■■■■■■■■■■■■□□□□□	animal	BOV	animal
1863	■■□■■□■□□□□□■■■□■■■■■■■■■■■■□■■■■■■■■■□□□□□	animal	BOV2	animal
1864	■□□□□□□□□□□□□■□■■■■■■■■■■■■■□□□□■□■■■■□■■■■	EAI	EAI5	EAI
1865	■■■■□■■■■■■■■■□■■■■■■■■■■■■■□□□□■□■■■■□□■■■	EAI	EAI5	EAI
1866	■□□■■■■■■■■■■■■■■□□■■■■■■■■■□□□□■□■■■■■■■■■	EAI	EAI5	EAI
1867	■■■■■■□□□■■■■■■■■■■■■□□□■■■■■■■■■■■■■■□■□■■	AFRI	AFRI1	Unassignable
1868	■■□■■■■■■■■■■■■■■■■□□■■■■■■■□□□□■□■■■■■■■□■	EAI	EAI2	EAI
1869	■■■■■■■■■■■■□□□□□□□□□□□□□□□□□□□□□□□□□□□□□□□	U	U	Unassignable
1870	□□□■■■■■■■■■■■■■■■■■□□□□■■■■■■■■□□□□■■■□■■□	LAM	LAM4	LAM
1871	■■■□□□□□□□□□□■■■■■■■■■□■□□□□□□■■□□□□■■■■■■■	T	T5	T
1872	■■■■■■■■■■■■■■■■■■■■■■■■■□□□□□■■□□□□■■■■■■■	T	T1	T
1873	■■■■■■■■■■■■■■■■□□■■□□□□■■□□□□■■□□□□■■■□■■■	LAM	LAM11	LAM
1874	■■■■■■■■□□■■■■■□□□□□□□□□□□■■■■■■□□□□□■■□■■■	U	U	Unassignable
1875	■□□■■■■■■□□□□□□□□■■■■■■■■■■■□□□□■□■■■■■■■■■	EAI	EAI5	EAI
1876	■■■■□□□□□□□□■■■■■■■■■■■■■■■■■■■■■■■■■■□■■■■	AFRI	AFRI1	Unassignable
1877	■■■□■■■■■□■■■■■■■■■■■■■■■■■■■■■■□□□□■■■■■■■	T	T1	T
1878	■■■□□□□■■■■■■■■■■■■■■■□□□□□□□□□□□□■■■■□□□□□	CAS	CAS1	CAS
1879	■■■■■■■■■■■■□■■■■■■□□□□□□□□■□□□□■□■■■■■□■■■	EAI	EAI1	EAI
1880	□□□□■■■■■■■□□□□□■■■■□□□□■■■■□□□□□□□□□□□□□■■	U	U	LAM
1881	□□□■■■■■■■■■■■■■■■□■■■■■■■■■□□□□■□■■■■■■■■■	EAI	EAI5	EAI
1882	■■■□□□□■■■■■■■■■■□■■□□□□□□□□□□□□□□■■■■■■■■■	CAS	CAS1	Unassignable
1883	■■■□□□□■■■■■■■■■■■■■■■□□□□□□□□□□□□■■■■■■■□□	CAS	CAS1	CAS
1884	■■■■■■■■■■■■■■■■□□■■■■■■■■■□□□□□□□□□□□■■■■■	U	U	Unassignable
1885	□□□□■■■■■■■□□■■■■■□■■■■■■■■■■■■■□□□□■■■■■■■	T	T1	T
1886	■■□□■■■■■■■■■■■■■■■□□■■■■■■■□□□□■□■■■■■■■■■	EAI	EAI5	EAI
1887	■■■□□□□■■■■■■■■■■■■■■□□□■■■■■■■■■□□□□■■■■■■	U	U	CAS
1888	■■□■■■■■■■■■■■■■■■■■■□□□■■■■■■■■□□□□■■■■■■■	T	T1	T
1889	□□□□□□■■■■■■■■■■■■■■■□□□■■■■■■■■□□□□■■■■■■■	T	T1	T
1890	■■■■■■■■■■■■■■■■■■■■■□□□■■■■■■■■□□□□■■■□■■■	T	T2	T
1891	■■■■■■■■■■■■■■■■■■■■■■■■□■■■■■□■□□□□■■■■■■■	H	H3	H
1892	■■■■■■■■■■■■■■■■■■■■□■■□□□□□□□□□□□□□□□■■■■■	U	U	Unassignable
1893	■■■■■■■■■■■■■■■■■■■□■■■■■■■■■□□■□□□□■■■■■■■	H	H3	T
1894	■■■■□□■■■■■■■■■■■■■■□□□□■■■■■■■■□□□□■■■■■■■	LAM	LAM9	LAM
1895	■■■■■■■■■■■■■■■■■■■■□□□□■■■■□□■■□□□□■■■□■■■	LAM	LAM4	LAM
1896	■■■■■■■■■■■□□■■■■■■■■■■■■■■■■■■■□□□■■■□□■■	MANU	MANU3	Unassignable
1897	■■■■■■■■■■■■■■■■■■■■■■■■□□□□■□□□□□■■□■■■■■■	U	U	Unassignable
1898	■■■■■■■■■■■■■■■■■■■■■■■■□□□□□□□□□□■■□■■■■■■	EAI	EAI7	Unassignable
1899	■■□■■■■■■■■■□■□□□□□□□□□□□■■■■■■■■□□□□■■■■■■■	T	T1	T
1900	■□■■■■■■■■■■■■■■■■■■■■■■■■■■□□□□■□■■■■■■■■□	EAI	EAI5	EAI
1901	■■■■■■■■■■■■■■■■■■■■■□□■■□□■□□□□■□■■■■■■■■■	EAI	EAI4	EAI
1902	■■■□□□■■■■■■■■■■■■■■■■■■■□□■□□□□■□■■■■■□■■■	EAI	EAI4	EAI

1903	■■■□■■■■■■■■■■■■■■■■■■■■■□□■□□□□■□■■■■■■■■■	EAI	EAI4	EAI
1904	□□□□■■■■■■■□■■■■■□□□□□□□□□□□□□□□□□□□■■■■■■	U	U	Unassignable
1905	■■■■■■■■■■■■■■■■■■■■■■■■■■■■□□■■□□□□■■■■■■■	T	T1	T
1906	■■■■■■■■■■■■■■■■■■■■□□□□■□■■□■■■□□□□■■■■■■■	LAM	LAM6	LAM
1907	■■■■■■■■■■■■■■■■■■■■■■■■■■■■■■■■■■□□■■■■■■■	U	U	Unassignable
1908	■■■■■■■■■■■■■■■■■■■■■■■■■■□□□□□■□□□□■■■■■■■	H	H3	T
1909	■■■■■■■□■■■■■■■■■■■■■■■■■■■■■■■■□□□□■■■□□□□	T	T1	T
1910	■■□■■■■■■■■■□■■■■■■■■□□□■■■■■■■■□□□□■■■■■■■	T	T3	T
1911	■■□■■■■■■■■■□■■■■■■■■□□□■■■■■■■■□□□□■■■■■■□	T	T3	T
1912	■■■■■■■□■■■■■■□■■■■■■■□■■■■■■■■■□□□□■■■■■■■	T	T5	T
1913	■■■■■■■■■■■■■■□■■■■■■■□■■■■■■■■■□□□□■■■■■■■	T	T5	T
1914	□■■■■■■■■■■■■■■■■■■■□□□□■■■■□■■■□□□□■■■■■■■	LAM	LAM6	LAM
1915	■■■■■■■■□□■■■■■■■■■■■□■■■■■■■■■■□□□□■■■■■■■	T	S	T
1916	■■■■■■■■■■■■□□□□□■■■■■■■■■■■■■■■□□□□■■■■■■■	T	T1	T
1917	■□■■■■■■■■■■■■■■■■■■■■■■■■■■■■■■□□□□■■■■■■□	T	T1	T
1918	■■■■■■■■□□■■■■■■■■■■■■■■■■■■■■□□□□□□■■■■■■■	U	U	Unassignable
1919	■■■■■■■■■■■■■■■■■■□■■■■■■■■■■■□■□□□□■■■□■■■	H	H3	H
1920	■■■□□□□■■■■■■■■■■■■■■■■■□□□□□□□□□□□■■■■■■■■	U	U	CAS
1921	■□■■■■■■■■■■■■■■■■■□□□□□■■■■□■■■□□□□■■■□■■■	T	T2	T
1922	■■■■■■■■■■■■□□□□■□■■■■■■■■■■■■■■□□□□■■■■■■■	X	X1	X
1923	■■■■□■■■■■■■■■■■■■■■■■■■■■■■■■□□□□□□■■■■■■■	U	U	Unassignable
1924	■□■■■■■■■■■■■□□■■■■□□□□□■□□■■■■■□□□□■■□■■■■	LAM	LAM7	T
1925	■■■■□■■□□□□■□□□□□□■■■■■■■■■■■□■■■■■■■■■■■□■	U	U	Unassignable
1926	■■■■■■■■■■■■■■■■■■■■■■■■■■■■■■■■□□□□■■■□□□■	T	T1	T
1927	■■■■■■■■■■■■□■■■■■■■■■■□■□□□□□□■□□□□■■■■■■■	H	H1	T
1928	■■■■□■■■■■■■■■■■■■■■■■■■■■□□□□□□□□□□■■■■■■■	U	U	Unassignable
1929	■■■■■■■■■■■■■■■■■■■■■■■■■■■■■■■■□□□□□■■□□□□	U	U	Unassignable
1930	■□□■■■■■■■■■■■■■■■■■■□■■■■■■■■■■□□□□■■■■■■■	T	T1	T
1931	■■■■■□■■■□□□□□□□□□□□□□□□■■■■■■□■□□□□■■■□■■■	H	H3	H
1932	■■□■■■■■□■■■□■■□■■■■■■■■■■■■■■■■■■■■■■□□□□□	animal	BOV1	animal
1933	■■■■■■■■■■■■■■■■■■■■□□□□■■■■■■■■□□□□■■■■■■□	LAM	LAM9	LAM
1934	■■■■■■■■■■■■■■■■■■■■■□■□■□□□□□□■□□□□■■■■■■■	H	H1	T
1935	■■□■■■■■■■■■■■□■■■■■□□□□■■■■■■■■□□□□■■□■■■■	LAM	LAM1	LAM
1936	■□■■■■■■■■■■□■■■■■□□□■■■■■■■■■■■□□□□■■■■■■■	T	T1	T
1937	■■■■■■■■■■■■■■■■■■■□□□□□■□□■■■■■□□□□■■■■■□□	LAM	LAM7	T
1938	■■■■■■■■□□■■■■■■■■■■■■■■■■■■■■■■□□□□■■■■■■□	T	S	T
1939	■■■■■■■■■■■■■■■■■■■■■■■■■□■■■■□■□□□□■■■□■■■	H	H3	H
